# De novo design and evolution of an artificial metathase for cytoplasmic olefin metathesis

**DOI:** 10.1038/s41929-025-01436-0

**Published:** 2025-11-03

**Authors:** Zhi Zou, Indrek Kalvet, Boris Lozhkin, Elinor Morris, Kailin Zhang, Dongping Chen, Marco L. Ernst, Xiang Zhang, David Baker, Thomas R. Ward

**Affiliations:** 1 Department of Chemistry, University of Basel, Basel, Switzerland; 2 National Center of Competence in Research ‘Molecular Systems Engineering’, Basel, Switzerland; 3 Department of Biochemistry, University of Washington, Seattle, WA USA; 4 Institute for Protein Design, University of Washington, Seattle, WA USA; 5 Howard Hughes Medical Institute, University of Washington, Seattle, WA USA

**Keywords:** Biocatalysis, Protein design, Organic chemistry, Metals

## Abstract

Artificial metalloenzymes present a promising avenue for abiotic catalysis within living systems. However, their in vivo application is currently limited by critical challenges, particularly in selecting suitable protein scaffolds capable of binding abiotic cofactors and maintaining catalytic activity in complex media. Here we address these limitations by introducing an artificial metathase—an artificial metalloenzyme designed for ring-closing metathesis—for whole-cell biocatalysis. Our approach integrates a tailored metal cofactor into a hyper-stable, de novo-designed protein. By combining computational design with genetic optimization, a binding affinity (*K*_D_ ≤ 0.2 μM) between the protein scaffold and cofactor is achieved through supramolecular anchoring. Directed evolution of the artificial metathase yielded variants exhibiting excellent catalytic performance (turnover number ≥1,000) and biocompatibility. This work represents a pronounced leap in the de novo design and in cellulo engineering of artificial metalloenzymes, paving the way for abiological catalysis in living systems.

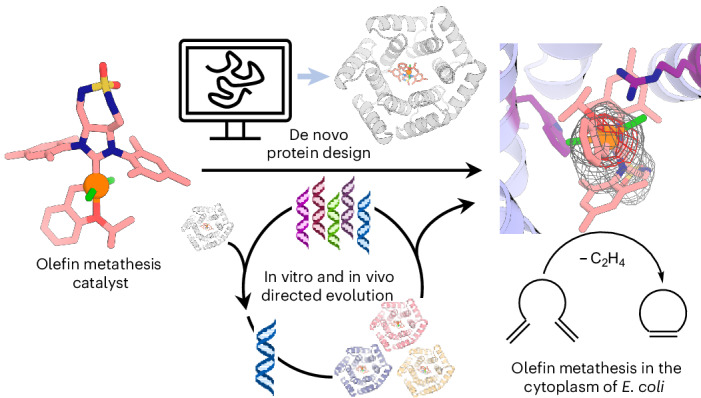

## Abstract (web summary)

The creation of artificial metalloenzymes compatible with complex biological settings could enable broad applications. Now a de novo-designed artificial metalloenzyme containing an abiological ruthenium cofactor is reported and optimized for ring-closing metathesis in the cytoplasm of whole cells.

## Main

Enzymes are gaining acceptance among the synthetic community, thanks to their catalytic benefits with regard to sustainability, step economy and exquisite selectivity^1,2^. Stimulated by these attractive features, efforts are underway to expand the catalytic repertoire of enzymes by designing artificial metalloenzymes (ArMs), which harbour a synthetic metal catalyst within a protein and catalyse new-to-nature reactions. Strategies for assembling ArMs rely on either substituting native metals/cofactors within native active sites^3–7^ or anchoring synthetic organometallic complexes into proteins. Such anchoring can be achieved either via covalent^8–12^, dative^13–16^ or supramolecular interactions^17–19^ between the cofactor and the protein. Although these strategies have proven fruitful both in homogenous^8,14,20^ and heterogenous systems^21,22^, the protein environment surrounding the cofactor—which substantially influences catalytic performance^23–25^—is by-and-large dictated by the anchoring moiety and thus may be incompatible with the ArM’s intended function. Accordingly, such ArMs often require further engineering efforts to improve their catalytic performance^15,26^. An additional challenge of ArMs is the modest compatibility of many synthetic cofactors with the complex whole-cell environment^27–30^. Accommodating and shielding these cofactors within a protein may offer a hospitable environment by minimizing (bimolecular) decomposition as well as inactivation by water and nucleophilic cell metabolites, such as glutathione (hereafter GSH)^19,25^. Over the past decade, notable progress has been achieved in expanding the scope of in cellulo biotransformations catalysed by ArMs, incorporating diverse metal cofactors including copper-^31^, gold-^20^, iridium-^32–36^, ruthenium-^37,38^ and rhodium-based cofactors^39^ (Supplementary Table 1). Despite these advances, most ArMs reported to date display only modest enhancements in catalytic performance—typically assessed by their turnover number (TON)—compared with their wild-type counterparts. Notable exceptions include a handful of highly active [Ir(Me)MPIX]-based systems, that catalyse carbene insertion^33^ or cyclopropanation^34^.

Olefin metathesis is a powerful and widely used transformation in organic synthesis and materials science, enabling the efficient and selective formation of carbon–carbon double bonds^40^. However, its application in chemical biology remains limited, as poor biocompatibility with cellular components often necessitates the use of (super-)stoichiometric amounts of catalyst to achieve acceptable conversions^19,41–45^. To overcome these limitations, we and others have explored the potential of artificial metathases—ArMs capable of catalysing olefin metathesis. These efforts have led to demonstrations of ArM activity in diverse biological environments, including body fluids^19,43^, the periplasm^37,46^, the cell surface^47^ and artificial membraneless organelles^45^ (Supplementary Table 1). Building on this, we hypothesized that a specifically tailored, de novo-designed host protein could provide enhanced tunability and stability, ultimately enabling the development of a best-in-class ArM for olefin metathesis in the cytoplasm of *Escherichia* *coli*.

**Fig. 1:**
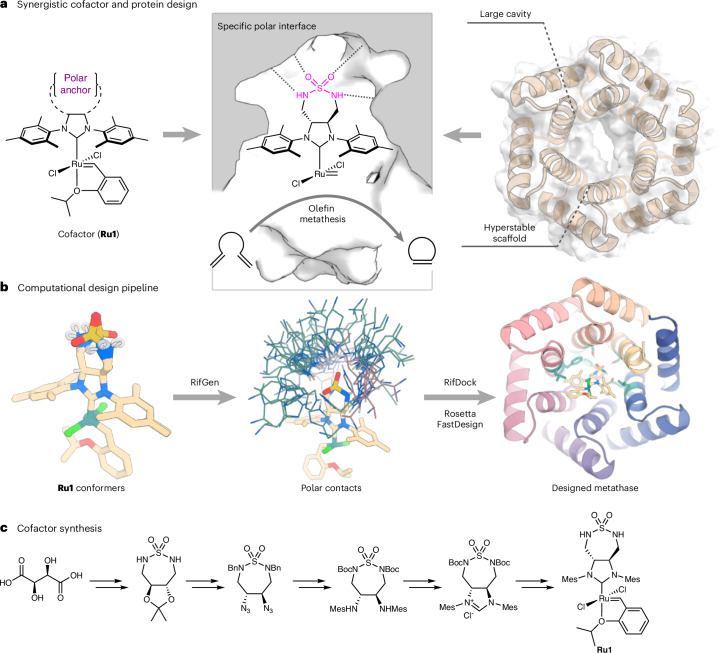
Creation of a de novo artificial metathase through synergistic cofactor and protein design. **a**, Modification of the Hoveyda–Grubbs second-generation olefin metathesis catalyst (**Ru1**) with a polar sulfamide anchoring group and a de novo-designed protein as binding partner. **b**, The computational design pipeline consists of generating polar contacts around the ligand using RifGen (displayed as a cloud of histidine rotamers), placement of the binding motif into the de novo protein with RifDock and sequence optimization with Rosetta FastDesign. **c**, The synthesis of **Ru1** from L-(+)-tartaric acid in sixteen steps (see Supplementary Methods for details).

We set out to design a Hoveyda–Grubbs olefin metathesis catalyst along with a de novo-designed protein that could house it in a manner optimal for catalysis (Fig. 1a). De novo protein design has matured to a stage where diverse protein scaffolds^48–50^ and tailored binding sites for various small molecules can be reliably designed^51–53^. We reasoned that these advances could enable us to design a hyper-stable protein that binds a catalytically competent cofactor exclusively via supramolecular interactions. From the catalyst perspective, we sought to address this challenge by designing a derivative of the Hoveyda–Grubbs catalyst (hereafter **Ru1**) that contains a polar motif, aimed at interacting via H-bonds with the protein, as well as improving the cofactor solubility in aqueous media (Fig. 1a). We reasoned that through computational protein design, the binding pocket could be tailored to provide complementary weak interactions with the cofactor **Ru1**. In addition, a hydrophobic pocket to interact with mesityl moieties of the cofactor and to harbour the catalytic event (Fig. 1b). We surmised that such synergistic design of abiotic cofactor and host protein could provide access to a greater variety of ArMs, unconstrained by the compatibility limits of existing systems.

In this study, we achieved this objective by creating an ArM that integrates the synthetic cofactor **Ru1** within a de novo-designed protein scaffold. The resulting artificial metathase catalysed ring-closing metathesis (RCM) of olefins in the cytoplasm of *E. coli*. Through directed evolution, its catalytic performance was substantially optimized (≥12-fold). Collectively, these findings demonstrate the feasibility of supramolecular anchoring of synthetic precious-metal cofactors within de novo-designed proteins. This strategy provides a versatile platform for creating and optimizing new-to-nature catalysis in cellulo.

## Results

### De novo design of host proteins to accommodate Ru1

With the **Ru1** catalyst at hand (Fig. 1c), we proceeded with designing proteins to bind to it. Since one of the key features of the catalyst is its polar sulfamide group, we sought to use this moiety as a guide for the computational design efforts. By using the RifGen/RifDock^50^ suite of programmes we enumerated the interacting amino acid rotamers around the cofactor and docked the ligand with a set of these residues into the cavities of de novo-designed proteins (Fig. 1b). The de novo-designed closed alpha-helical toroidal repeat proteins (such as Protein Data Bank (PDB) ID: 4YXX, hereafter dnTRP) were selected as the protein scaffold, owing to their high thermostability, engineerability and a suitably sized pocket for ligand-binding^52,54^. Docked structures containing the cofactor **Ru1** and the key interacting residues were then subjected to further protein sequence optimization (refining hydrophobic contacts with the ligand and stabilizing the key H-bonding residues) using Rosetta FastDesign^54^. The design models were subsequently evaluated for computational metrics describing the protein-cofactor interface and pre-organization of the binding pocket. This led us to select 21 designs (dnTRP, hereafter) for experimental testing (Supplementary Methods).

### Identification of the most promising dnTRP

Each of the 21 dnTRPs, featuring an N-terminal hexa-histidine tag and a TEV protease cleavage sequence, were expressed in *E. coli*. SDS–polyacrylamide gel electrophoresis analysis revealed that 17 of these were expressed mostly in the soluble fraction; these were purified by nickel-affinity chromatography (Supplementary Fig. 2).

**Fig. 2:**
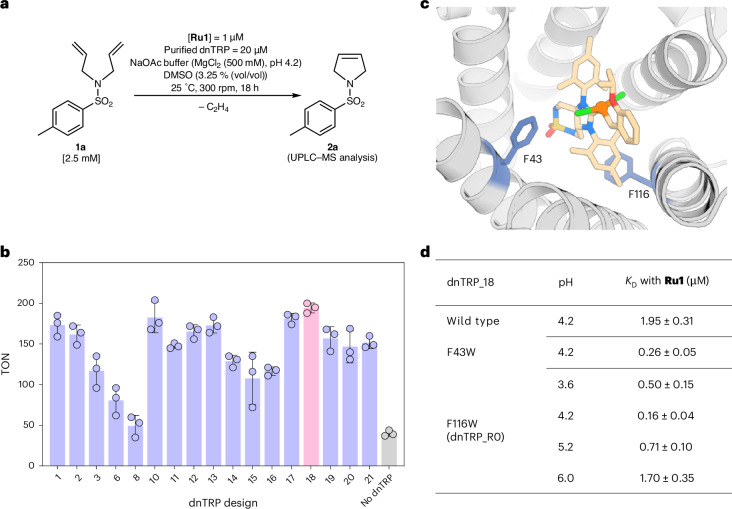
Selection and optimization of dnTRPs for assembly of **Ru1**·dnTRPs. **a**, Substrate **1a** and RCM reaction conditions using purified dnTRPs. **b**, The catalytic performance (TON) for the RCM of substrate **1a** in the presence of the **Ru1**·dnTRP using the 17 dnTRPs as host proteins. The data are displayed as mean values of three replicates with error bars indicating standard deviations (*n* = 3). **c**, A computed model of dnTRP_18 highlighting two residues F43 and F116 (blue sticks), which were individually mutated to tryptophan to increase hydrophobicity around the **Ru1** cofactor. The **Ru1** cofactor (colour-coded sticks) and the ruthenium atom (orange sphere) are displayed. **d**, A summary of the binding affinity (*K*_D_) of **Ru1** for dnTRP_18 and single mutants thereof at various pHs. The data are displayed as mean values of three replicates ± standard deviation (*n* = 3). The replicates for **b** and **d** were independently performed using the same stock of purified dnTRPs. The tryptophan fluorescence-quenching assay and the fitting procedure to derive the *K*_D_ are presented in the Supplementary Methods and Supplementary Fig. 4. Source data

To identify the most promising scaffold for RCM, we evaluated the 17 purified dnTRPs treated with **Ru1** (0.05 equivalents (equiv.) versus dnTRP) in the presence of the diallylsulfonamide **1a** (5,000 equiv. versus **Ru1**) as prototypical RCM substrate (Fig. 2a). Under standard RCM conditions (that is 18 h, pH 4.2) all artificial metathases (hereafter **Ru1**·dnTRPs) afforded higher TONs than the free cofactor **Ru1** (TON 40 ± 4), with dnTRP_10, dnTRP_17 and dnTRP_18 performing best (TON 183 ± 19, 181 ± 7 and 194 ± 6, respectively) (Fig. 2b). In light of its high expression level, we selected dnTRP_18 for the remainder of the study.

Stability studies of the *apo* dnTRP_18 revealed tolerance towards pH values ranging from 2.6 to 8.0 and a pronounced thermal stability, with a *T*_50_ > 98°C (*T*_50_: temperature at which 50% of the protein is denatured after 30-min incubation; Supplementary Fig. 3), in accordance with previous reports on structurally related dnTRPs^55^. Next, we determined the binding affinity of **Ru1** for dnTRP_18 using a tryptophan fluorescence-quenching assay (*K*_D_ = 1.95 ± 0.31 μM) (Supplementary Fig. 4). To further improve the affinity and ensure near quantitative binding at low micromolar concentrations of dnTRP_18, we set out to increase the hydrophobicity around the **Ru1** binding site. For this purpose, positions F43 and F116 were individually mutated to tryptophan (Fig. 2c). Both dnTRP_18_F43W and dnTRP_18_F116W (hereafter dnTRP_R0) displayed a nearly tenfold higher affinity with *K*_D_ = 0.26 ± 0.05 and 0.16 ± 0.04 μM at pH 4.2, respectively (Fig. 2d). Native mass spectrometry and size-exclusion chromatography further highlighted the binding between **Ru1** and dnTRP_R0 and the formation of the **Ru1**·dnTRP_R0 complex with a 1:1 stoichiometry (Supplementary Fig. 5).

### Directed evolution of Ru1·dnTRP

**Fig. 3:**
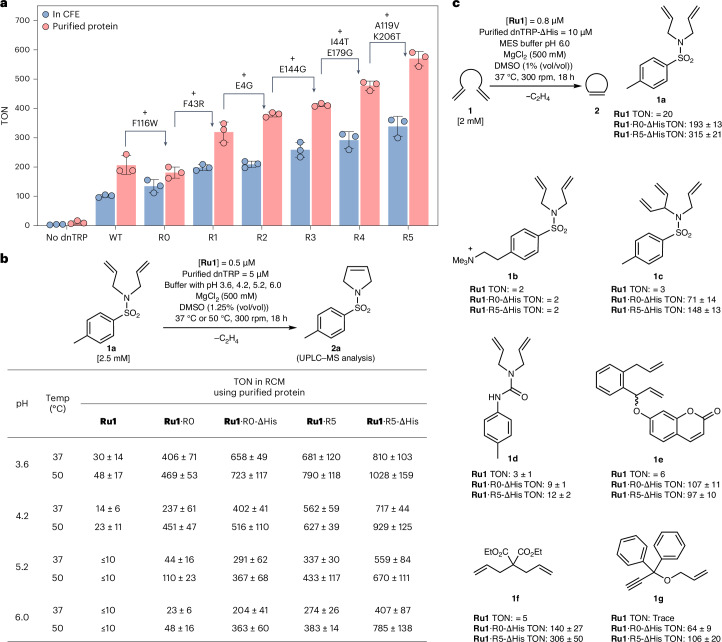
Improving metathase activity of **Ru1**·dnTRP by directed evolution. **a**, A summary of the TON (at pH 4.2, 18 h) of selected **Ru1**·dnTRPs along the evolutionary trajectory, using CFE (blue bars) or purified samples (salmon bars). The beneficial mutations identified during each evolutionary round are highlighted with an arrow. The data for TONs are presented as mean values of three replicates (*n* = 3), with the error bars representing standard deviation. For the CFE assay, biological replicates were performed. For purified proteins, replicates were independently performed using the same stock of purified dnTRPs. **b**, The effect of pH and temperature (Temp) on the TON for RCM of substrate **1a** using purified dnTRP and dnTRP-ΔHis proteins (the N-terminal hexa-histidine and TEV cleavage sequence were removed proteolytically) (Supplementary Fig. 9). **c**, Substrate scope of purified **Ru1**·dnTRP-ΔHis. The data in **b** and **c** are displayed as mean values ± standard deviation of three replicates (*n* = 3). The replicates were independently performed using the same stock of purified dnTRPs. WT, wild type. The details regarding the RCM conditions, sample processing and product quantification are summarized in the Methods, Supplementary Methods and Supplementary Fig. 12. Source data

Directed evolution is a preeminent methodology for engineering of natural enzymes and ArMs to improve their catalytic performance^56–58^. To facilitate streamlined engineering of artificial metathases, we sought suitable conditions for the RCM screening using *E. coli* cell-free extracts (CFE). Based on the **Ru1**·dnTRP_R0 pH-affinity profile (Fig. 2d), we prepared the CFE at pH 4.2 and supplemented the reaction mixture with bis(glycinato)copper(II) [Cu(Gly)_2_]—which had been shown previously to partially oxidize GSH present in cell lysates^35^—thus enabling screening **Ru1**·dnTRP in CFE (compare 197 ± 7 TON with [Cu(Gly)_2_] = 5 mM versus 152 ± 16 TON in untreated CFE) (Supplementary Fig. 6a). To reflect the typical dnTRP concentrations obtained in the CFE from a 1 ml culture in a 96-well plate, we set [**Ru1**] = 0.5 μM, thus ensuring its near-quantitative binding to dnTRPs (Supplementary Figs. 6b and 7). Relying on this protocol, we established a high-throughput endpoint screening assay in a 96-well plate format for the directed evolution of **Ru1**·dnTRP, starting from **Ru1**·dnTRP_R0 (hereafter **Ru1**·R0) (Supplementary Fig. 8a). The first three rounds of directed evolution involved screening iterative site-saturation mutagenesis (SSM) libraries by targeting amino acid residues in the proximity of the computed position of **Ru1**. Following the screening using CFE, the most promising variants were validated with purified dnTRPs. The most promising mutants for each round are abbreviated as **Ru1**·R1 (that is, **Ru1**·dnTRP_18_F43R/F116W, TON 319 ± 34), **Ru1**·R2 (that is, **Ru1**·dnTRP_18_E4G/F43R/F116W, TON 379 ± 8) and **Ru1**·R3 (that is **Ru1**·dnTRP_18_E4G/F43R/F116W/E144G, TON 412 ± 6) (Supplementary Fig. 8b–e). For the fourth round, we screened an error-prone PCR (epPCR) library (1,800 colonies) relying on **Ru1**·R3 and identified **Ru1**·R4 (that is, **Ru1**·dnTRP_18_E4G/F43R/I44T/F116W/E144G/E179G) with 2.6-fold and 43-fold increased TON over **Ru1**·R0 and **Ru1**, respectively (Supplementary Fig. 8f,g). For the fifth round, we screened a fragment shuffling library (540 colonies) by randomly recombining the beneficial mutations from third and fourth rounds (Supplementary Fig. 8h). This led to the identification of variant **Ru1**·R5 (that is, **Ru1**·dnTRP_18_E4G/F43R/I44T/F116W/A119V/E144G/E179G/K206T) that afforded TON = 339 ± 34 and 570 ± 25 in CFE and purified form, respectively (Fig. 3a).

With this evolved variant at hand, we evaluated the metathase’s performance at higher pH values, with the ultimate goal of performing RCM in *E. coli* whole cells. The performance of Ru-based metathesis catalysts in aqueous solution is negatively impacted by basic media^27,59^. To evaluate the effect of the directed evolution on the pH-dependent metathase activity of **Ru1**·dnTRPs, we compared the performance of **Ru1**·R5 with that of **Ru1** and **Ru1**·R0 at various pHs (Fig. 3b). Gratifyingly, the pH-tolerance along the evolutionary trajectory closely follows the TON trends: **Ru1**·R5 > **Ru1**·R0 > **Ru1**, highlighting the beneficial effect of the dnTRP scaffold and the directed evolution trajectory. The evolved metathase **Ru1**·R5 maintained nearly half of its activity at pH 6.0 (versus pH 4.2), whereas **Ru1**·R0 lost nearly ninety percent of its activity. Only traces of the product **2a** (for example, TON ≤ 10) were detected at pH ≥5.2 in the presence of the free cofactor **Ru1** (Fig. 3b).

### Evaluation of the catalytic performance of the Ru1·dnTRPs with purified samples

To evaluate the effect of the N-terminal his-tag on both catalytic performance and cofactor affinity, we removed it via TEV protease cleavage (hereafter dnTRP-ΔHis). Removal of His-tag resulted in lower *K*_D_ value at pH 6.0 (Fig. 2d versus Supplementary Fig. 10a). We surmise that removal of His-tag may minimize undesirable interactions with **Ru1** (pH 6.0) and thus contributes to lower the *K*_D_. The corresponding dnTRP-ΔHis variants proved more active than the variants containing the Lewis-basic affinity tag, especially at a higher pH (Fig. 3b). We then evaluated the effect of both temperature and pH on the RCM’s activity. The highest TON was achieved at 50 °C. At pH 3.6 and 50 °C, **Ru1**·R5-ΔHis afforded a TON of 1,028 ± 159 (Fig. 3b). Notably, **Ru1**·dnTRPs retain ≥40% activity at 90 °C at both pH 4.2 and 6.0 (Supplementary Fig. 11a). Unfortunately, all attempts to express dnTRP-ΔHis in *E. coli* lead to markedly lower yields, thus challenging its use in *E. coli* whole-cell studies (Supplementary Fig. 10b).

To assess the substrate scope of the **Ru1**·dnTRPs, five dienes, one triene and one enyne substrate were tested in the presence of **Ru1**, **Ru1**·R0-ΔHis and **Ru1**·R5-ΔHis (Fig. 3c and Supplementary Fig. 12). Comparison of the RCM performance highlights that the **Ru1**·dnTRPs accept various substrates, leading to substantially improved TONs compared with the free cofactor **Ru1**. Except for substrate **1e**, the fifth generation variant **Ru1**·R5-ΔHis lead to improved TONs compared to **Ru1**·R0-ΔHis. The presence of an ammonium group on the diene **1b**, nearly completely shuts down RCM activity, both for the free cofactor **Ru1** and for the **Ru1**·dnTRPs. No enantioselectivity was observed for the RCM of the prochiral triene **1c**.

### Structural characterization of Ru1·dnTRPs

**Fig. 4:**
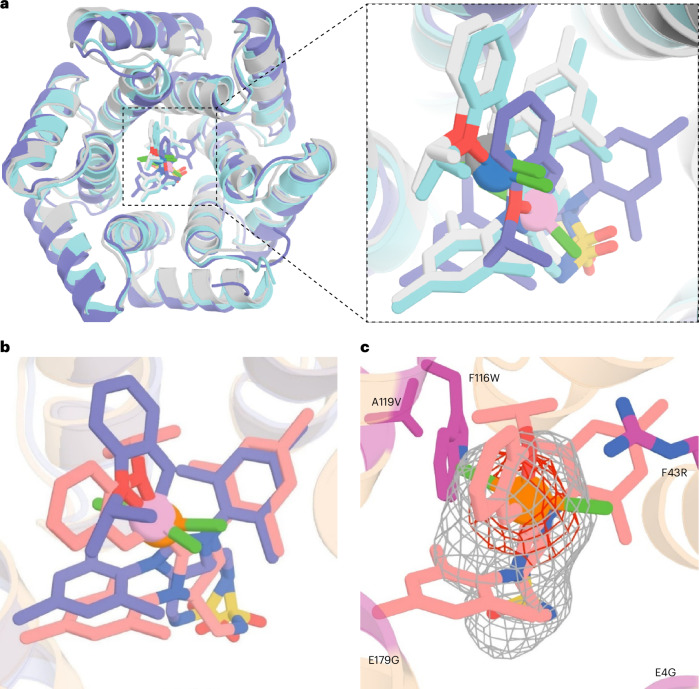
Structural analysis of **Ru1**·dnTRPs. **a**, An overlay of the design models of **Ru1**·dnTRP_18 (grey, ruthenium: dark grey sphere), **Ru1**·R0-ΔHis (cyan, ruthenium: blue sphere) and the X-ray structure of **Ru1**·R0-ΔHis (purple, ruthenium: pink sphere, PDB: 8S6P). **b**, Expanded overlay view around the **Ru1** cofactor for **Ru1**·R0-ΔHis (purple, ruthenium: pink sphere) and **Ru1**·R5-ΔHis (wheat, ruthenium: orange sphere, PDB: 9H3C). **c**, Expanded view of the inner cavity **Ru1**·R5-ΔHis. The ruthenium cofactor (colour-coded sticks, Ru: orange sphere) and the mutated residues (magenta) identified in the directed evolution are highlighted. *Fo*–*F*c omit map contoured at 1σ (grey) highlighting the approximate cofactor position, and anomalous electron density map contoured at 1.0σ (red) highlighting the position of the ruthenium. All the protein scaffolds are displayed as a cartoon representation.

We obtained X-ray crystal structures of *apo* dnTRP_R0-ΔHis (resolved to 1.6 Å, PDB: 9GVF), *holo* **Ru1**·R0-ΔHis (resolved to 2.9 Å, PDB: 8S6P) and **Ru1**·R5-ΔHis (resolved to 2.9 Å, PDB: 9H3C). A comparison of *apo* and **Ru1**·dnTRP_R0-ΔHis X-ray structures with computational models reveals an overall agreement with the toroidal shape but notable deviations with regards to the shape of the inner cavity, as well as the position the **Ru1** cofactor. Specifically, the X-ray structure of **Ru1**·R0-ΔHis reveals a cylindrical pocket, in contrast to the conical shape predicted for **Ru1**·R0-ΔHis and **Ru1**·dnTRP_18 with AlphaFold2 (AF2)^60^. The Cα root mean square deviation values between the AF2 predicted models and the X-ray structures range from 1.59 to 1.63 Å (Fig. 4a and Supplementary Fig. 13a,b). The ruthenium’s position in the **Ru1**·R0-ΔHis X-ray structure (as judged from the ruthenium’s anomalous density) is shifted by 3.4 Å compared with the **Ru1**·dnTRP_18 model (Supplementary Fig. 13c). Consequently, compared with the AF2 model, the TRP amino acid side chains that interact with the cofactor **Ru1** differ. The residues predicted by AF2 to interact with **Ru1** include S11, S46, Y50 and E186 as primary contributors. Instead, the X-ray structure reveals the closest contacts between residues Y50 and K190 with sulfamide anchor (Supplementary Fig. 13d,e). Surprisingly, alanine substitution at Y50A and K190A in dnTRP_R5-ΔHis—residues initially designed to interact with the sulfamide moiety of **Ru1** via hydrogen bonding—led to only a modest decrease in affinity (that is, ≤2.3-fold increase in *K*_D_), suggesting that hydrophobic interactions may play a more prominent role in cofactor binding than previously anticipated (Supplementary Fig. 10a). The X-ray structure of **Ru1**·R5-ΔHis displays close structural similarity to **Ru1**·R0-ΔHis, with a backbone ΔHis of 0.6 Å and a Ru atom deviation of 1.0 Å between the two structures (Fig. 4b and Supplementary Fig. 13f). Notably, compared with **Ru1**·R0-ΔHis, the evolved variant **Ru1**·R5-ΔHis features an expanded and less hydrophilic channel leading to the active site, which results from the three critical E4G, E144G and E179G mutations (Fig. 4c and Supplementary Fig. 13g,h). These probably contribute to the increased affinity of dnTRP_R5-ΔHis (versus dnTRP_R0-ΔHis; Supplementary Fig. 9c) and hinder the approach of hydrophilic species (including GSH, OH^−^ and so on) that lead to cofactor inhibition.

In developing a computational model of **Ru1**·R5-ΔHis, the structure was predicted using AlphaFold2 and further refined using Rosetta FastRelax in the presence of **Ru1**. Yet, the resulting models did not accurately reflect the deeper placement of the **Ru1** cofactor as observed in the crystal structure. Attempts to correct the ligand placement with both physics-based Rosetta GALigandDock^61^ and deep-learning-based tools like AlphaFold3 (ref. ^62^), Chai-1 (ref. ^63^), Boltz-1 (ref. ^64^) and PLACER^65^ were relatively unsuccessful, with the deep-learning-based methods notably struggling with predicting the precise geometry of **Ru1** possibly due to lack of training examples with similar structures (Supplementary Fig. 14d,e). Chai-1 (ref. ^63^) showed improved placement of **Ru1** in the expected orientation, and Ru atom within 1 Å of X-ray, albeit still struggling with the exact geometry of **Ru1** (Supplementary Fig. 14a–c). These discrepancies highlight the challenges faced by current computational models in accurately predicting the complex interplay between protein folds and the unique nature of organometallic cofactors, emphasizing the urgent need for improved modelling techniques capable of handling such chemically diverse cofactors.

### Whole-cell RCM catalysed by Ru1·dnTRP-ΔHis

**Fig. 5:**
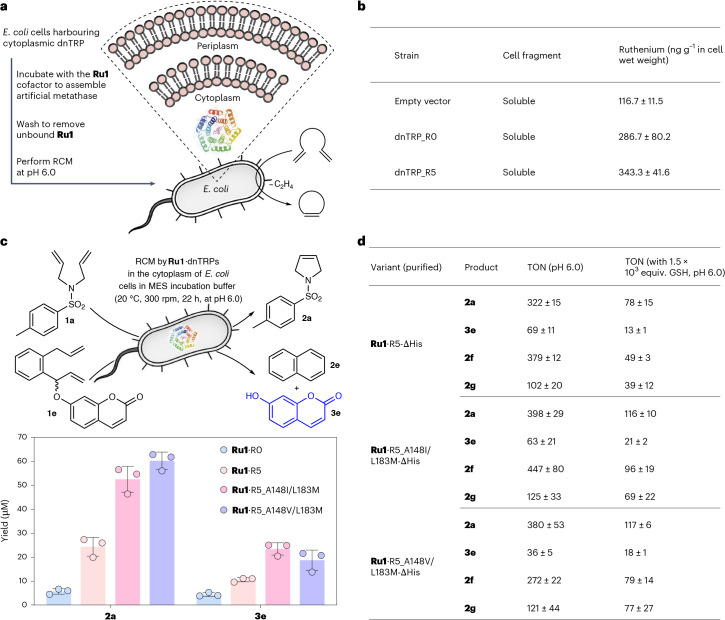
RCM in the cytoplasm of *E. coli.* **a**, A schematic representation of the protocol applied for *E. coli* whole-cell RCM by **Ru1**·dnTRPs. **b**, Ruthenium content in the soluble fragment of *E. coli*, determined by ICP-MS. **c**, RCM of substrates **1a** and **1e** by the evolved variants **Ru1**·R5_A148I/L183M and **Ru1**·R5_A148I/L183M in the cytoplasm of *E. coli*. For the RCM of **1e**, the TON was determined by quantifying the product **3e** (by fluorescence) (Supplementary Fig. 18). The results represent the mean of three biological replicates with the error bars indicating standard deviations (*n* = 3). MES incubation buffer: 50 mM, MgCl_2_ (100 mM), glycerol (5% (vol/vol)), 0.02 % (wt/vol) poloxamer 188, pH 6.0. **d**, A summary of the TONs obtained for the evolved **Ru1**·dnTRP-ΔHis variants, using purified samples for the RCM of substrates **1a**, **1e**, **1f** and **1g** (yielding the products **2a**, **3e**, **2f** and **2g**, respectively), both in the absence and the presence of glutathione (GSH). The data in **d** are displayed as mean values ± standard deviations of three replicates (*n* = 3). The replicates were independently performed using the same stock of the purified dnTRP-ΔHis proteins. Source data

In light of the remarkable improvement in catalytic performance of **Ru1**·R5, we set out to evaluate its RCM activity in the presence of GSH. For this purpose, we spiked purified samples of **Ru1**·R5-ΔHis with increasing concentrations of GSH (Supplementary Fig. 11b). In contrast to the free cofactor **Ru1**, the dnTRP-ΔHis host protein protects the thiophilic **Ru1** cofactor from poisoning by GSH: at [GSH] = 1.28 mM, **Ru1**·R5-ΔHis maintains > 20% of its RCM activity, whereas **Ru1** is completely inactivated at [GSH] ≥ 40 μM. Encouraged by these findings, we tested whether RCM activity could be detected in the cytoplasm of *E. coli*. To safeguard *E. coli*’s viability, whole-cell metathesis experiments were performed at pH 6.0. For this purpose, we treated *E. coli* cells expressing cytoplasmic dnTRP_R0 and dnTRP_R5 with varying concentrations of **Ru1** (that is 1 ≤ [**Ru1**] ≤ 10 μM). We used cytoplasmic dnTRR instead of dnTRR-ΔHis, as the former exhibited a markedly higher expression level and facilitated its subsequent purification (Supplementary Fig. 15a). Following incubation and thorough washing, the substrate **1a** was added to the cell suspension and RCM activity (at pH 6.0) was quantified by ultra-performance liquid chromatography. At [**Ru1**] ≤ 2 μM, the cells expressing cytoplasmic dnTRP_R5 exhibited notably higher yields of product **2a**, compared to dnTRP_R0 (Supplementary Fig. 15b,c). Cell viability after RCM was evaluated relying on a colony forming assay, confirming the whole-cell compatibility of RCM catalysed by **Ru1**·R5 ( > 50 % colonies remaining after whole-cell RCM, Supplementary Fig. 15d). Inductively coupled plasma mass spectrometry (ICP-MS) analysis revealed that both dnTRP-expressing strains accumulated more than 2.5-fold higher Ru levels compared with *E. coli* harbouring the empty plasmid. Although the mean Ru concentration in cells expressing dnTRP_R5 (343.3 ± 41.6 ng g^−1^ wet cell weight) was higher than in those expressing dnTRP_R0 (286.7 ± 80.2 ng g^−1^), the difference was not statistically significant (*P* > 0.05, unpaired two-tailed *t*-test, *n* = 3) (Fig. 5b and Supplementary Fig. 16).

With this activity screen, we set out to further evolve **Ru1**·R5 for enhanced RCM activity in *E. coli* whole cells (Supplementary Fig. 17a). Guided by the X-ray structure of **Ru1**·R5-ΔHis, we selected four residues (L8, L113, A148 and L183) (Supplementary Fig. 17b) located in the proximity of the ruthenium for randomization with 17 amino acid residues (except Cys and Pro). The activity of these 68 variants was evaluated using *E. coli* whole cells for RCM of substrate **1a**. All beneficial mutations resulted from the introduction of hydrophobic residues (Supplementary Fig. 17c). The most active variants **Ru1**·R5_A148I and **Ru1**·R5_L183M were further recombined with hydrophobic residues of Ala, Phe, Gly, Ile, Met and Val at position 183 and Phe, Gly, Ile, Leu, Met and Val at position 148, respectively. The variant **Ru1**·R5_A148V/L183M exhibited the highest cytoplasmic RCM activity (2.5- and 10.6-fold versus **Ru1**·R5 and **Ru1**·R0, respectively) (Fig. 5c). Although reduced GSH is a major intracellular inhibitor of precious-metal-based catalysis, it is probably not the only cytoplasmic metabolite that compromises ArM activity. Notably, the evolved artificial metathase exhibited markedly higher activity than its parent in whole-cell experiments, suggesting that directed evolution has minimized the impact of intracellular deactivating factors. This improved biocompatibility enables effective RCM in the cytoplasm of *E. coli*, as further illustrated by the in situ release of umbelliferone **3e**, an RCM reaction with potential utility for intracellular signalling or prodrug activation^19,46^ (Fig. 5c).

Next, the binding affinity and catalytic performance of these evolved variants were investigated using purified dnTRP-ΔHis (Supplementary Fig. 19a,b). **Ru1**·R5_A148I/L183M-ΔHis and **Ru1**·R5_A148V/L183M-ΔHis exhibited comparable binding affinity to **Ru1**·R5-ΔHis (both at pH 3.6 and 6.0). To validate the biocompatibility, activity profiles of purified **Ru1**·dnTRP variants were evaluated across a range of GSH concentrations (0.25 ≤ [GSH] ≤ 4 mM, corresponding to 250–4,000 equiv. versus **Ru1**), reflecting the physiological concentrations in the cytoplasm^66,67^. Gratifyingly, all variants exhibited improved TON in the RCM of **1a**, with up to a 2.1-fold increase (versus **Ru1**·R5-ΔHis at pH 6.0) (Supplementary Fig. 19c). Interestingly, the purified variant **Ru1**·R5_A148I-ΔHis afforded superior TONs compared with variants **Ru1**·R5_A148I/L183M-ΔHis and **Ru1**·R5_A148V/L183M-ΔHis in the presence of GSH, despite the fact that the latter two variants exhibited higher yields of product **2a** in the cytoplasmic assay. In addition, to highlight the improved shielding ability of the dnTRP variants, we evaluated the RCM performance of purified **Ru1**·dnTRP-ΔHis variants with substrates **1a**, **1e**, **1f** and **1g** in the presence of [GSH] = 1.5 mM. Gratifyingly, these variants afforded markedly higher TONs than with **Ru1**·R5-ΔHis for RCM products **2a**, **3e**, **2f** and **2g** (Fig. 5 and Supplementary Fig. 20). Collectively, these results highlight the adaptation of the evolved artificial metathases to the deleterious effects of thiols in the cytoplasm of *E. coli* and demonstrate their robustness and feasibility for performing RCM in living systems.

## Conclusion

This study presents an example of combining an artificial precious-metal cofactor **Ru1** with a de novo-designed tandem-repeat protein dnTRP. The resulting ArM catalyses ring-closing olefin metathesis, a new-to-nature reaction. The remarkable stability of the dnTRP markedly simplified the directed evolution protocol, enabling the screening of CFEs. By relying on an endpoint assay, this screening protocol led to the identification of an evolved octuple mutant **Ru1**·R5 exhibiting ≥12- and 40-fold increase in TON compared with the parent enzyme **Ru1**·R0 and the free cofactor **Ru1**, respectively. The evolved variant **Ru1**·R5 proved active in the cytoplasm of *E. coli*, thus enabling the further evolution of metathase activity in whole cells. By achieving over a 5.4-fold increase in TON in *E. coli*’s cytoplasm for the bioorthogonal uncaging of fluorophore **3e**, the evolved artificial metathase highlights its potential for in cellulo applications, including real-time bioimaging and targeted prodrug activation. The X-ray crystal structure of both starting and evolved variants revealed discrepancies with the computed design, highlighting the challenges in the computational modelling of protein and organometallic systems simultaneously and suggesting possible avenues for further improvements in docking and prediction algorithms. To complement previously reported de novo ArMs, our system features a **Ru1** cofactor anchored exclusively through weak, non-covalent interactions—rather than via dative or covalent bonds with amino acid side chains^52,68,69^. This distinctive feature, combined with the modularity of synthetic strong-field ligands coordinated to platinum-group metals, paves the way for expanding the synthetic biology repertoire towards abiotic transformations within whole-cell enzyme cascades. Collectively, these findings represent a major leap in the de novo design and evolution of ArMs for cytoplasmic catalysis. These underscore the potential of integrating computational design and directed evolution for creating and optimizing ArMs, paving the methodology for building in cellulo new-to-nature catalysis beyond natural or repurposed enzymes.

## Methods

### Generation of mutational libraries of dnTRP

The SSM libraries of dnTRP_R0 at positions (Q5, E39, F43, E74, L78, L113, E144, A148 and L183) in the first round of screening were generated using dnTRP_18 _F116W (dnTRP_R0) as the template. The SSM libraries for the second round of screening were generated at positions (E4, Q5, E39, E109, E144 and E179) using dnTRP_18_ F43R/F116W (dnTRP_R1) as the template. The SSM libraries for the third round of screening were generated at positions (Q5, E39, E144 and E179) using dnTRP_18_E4G/F43R/F116W (dnTRP_R2) as the template (Supplementary Fig. 8b). The primers used for PCRs are listed in Supplementary Table 2. The PCR products were digested with DpnI (37 °C, 20 h), cleaned and intramolecularly cyclized using the Golden Gate assembly or Gibson assembly kit. The cyclized products were individually transformed into *E. coli* Top10 Chemically Competent Cells, plated on lysogeny broth (LB) agar plate (supplemented with 50 μg ml^−1^ kanamycin) and cultivated (37 °C, 20 h). The colonies from each library were pooled and the plasmids of the colonies were isolated by miniprep. The resulting plasmids were then individually transformed into *E. coli* LEMO21 chemically competent cells.

The epPCR library was generated using the dnTRP_R3 (dnTRP_18_E4G/F43R/F116W/E144G) as a template. In brief, epPCR was conducted using Taq polymerase 2X Master Mix supplemented with varying concentrations (from 0.1 to 0. 5 mM) of MnCl_2_. The PCR products were digested with DpnI (37 °C, 16 h), cleaned and assembled into the pET-29b vector using the Golden Gate assembly kit. The assembled products were transformed into *E. coli* Top10 Chemically Competent Cells, plated on LB agar plates (supplemented with 50 μg ml^−1^ kanamycin) and cultivated (37 °C, 20 h). Mutational frequencies were assessed by sequencing 16 random colonies from each MnCl_2_ concentration, and the results are summarized in Supplementary Fig. 8f. The selected library with a mutational frequency of 4.1 (at 0.15 mM MnCl_2_) underwent further processing and transformation into *E. coli* LEMO21.

The fragment shuffling library was generated using Gibson Assembly. In brief, the DNA sequence of dnTRP was separated into five fragments (with sequence lengths varying from 160 to 190 bp) and individually amplified (Supplementary Table 3). Plasmids of selected variants from rounds 3 and 4 were used as the templates for the PCR amplification of the fragments (Supplementary Fig. 8). After DpnI digestion (37 °C, 16 h) and cleanup, the fragments were assembled with the pET-29b vector backbone using the Gibson assembly. The assembled products were transformed into *E. coli* DH5a electro-competent cells, plated on LB agar plates (supplemented with 50 μg ml^−1^ kanamycin) and cultivated (37 °C, 20 h). The colonies were pooled and their plasmid DNA was extracted, followed by transformation into *E. coli* LEMO21. The colonies were inoculated in the culture (1 ml, ZYP auto-induction medium) in the 96-well plate to express dnTRP, as described in the Supplementary Methods.

### Development of the high-throughput screening assay in the 96-well plate

The stock solution of **Ru1** for RCM with the substrate **1a** in the screening assay was prepared as follows. A stock solution of **Ru1** (1 mM in dimethyl sulfoxide (DMSO)) was first prepared. An aliquot (10 μl) of this solution was transferred into a glass vial (2 ml, clear robo vial, 9 mm thread, item no. VT009-1232) and chilled on ice (1 min). The ice-chilled NaOAc buffer (990 μl, 100 mM, MgCl_2_ (500 mM), pH 4.2) was then added to the vial and gently mixed on ice. The resulting **Ru1** solution ([**Ru1**] = 10 μM) was used for the RCM reaction.

The freshly prepared CFE (95 μl, Supplementary Methods) of the libraries was transferred into a new assay plate (MASTERBLOCK, 96 well, polypropylene (PP), 0.5 ml, V-bottom) using the Liquidator 96-channel benchtop pipette (volume range of 5–200 μl). After chilling the plate on ice for 15 min, the **Ru1** cofactor (5 μl, 10 μM stock in NaOAc buffer (100 mM, MgCl_2_ (500 mM), pH 4.2)) was added in the wells using a multichannel pipette. The plate was then covered with a thick aluminium sealing film (AlumaSeal 96 film) and incubated (30 °C, 250 rpm, 2 h). Then, the aluminium film was lifted and the substrate **1a** (1 μl, 250 μM stock in DMSO, final concentration 2.5 mM) was added using multichannel pipette. The plate was resealed and incubated (37 °C, 300 rpm, 18 h) for the RCM reaction. After incubation, the plate was chilled (10 min on ice) and methanol was added (400 μl, supplemented with benzyltriethyl-ammonium bromide (200 μM) as internal standard). The plate was resealed and incubated (37 °C, 300 rpm, 30 min) to quench the reaction. The plate was centrifuged (4 °C, 4,400*g*, 30 min) and the clear supernatant (350 μl) was transferred to a new analysis plate (MASTERBLOCK, 96 well, PP, 0.5 ml, V-bottom) and subjected to ultra performance liquid chromatography–mass spectrometry (UPLC–MS) analysis. The schematic presentation of the screening protocol, the step-to-step rounds of evolutionary campaigns and the identified variants from each round are displayed in Supplementary Fig. 8.

### RCM of different olefin substrates using purified Ru1·dnTRPs

RCM of substrates **1b**, **1c**, **1d**, **1e**, **1f** and **1g** to afford the corresponding cyclized products **2b**, **2c**, **2d**, **2e**, **2f**, **2g** and **3e**—corresponding to the product cogenerated with **2e** in RCM of **1e**—was performed using a modified protocol that was used for cyclized product **2a**. In brief, **Ru1** (5 μl from a freshly prepared stock (16 μM in ice-chilled 2-morpholinoethanesulfonic acid (MES) buffer (100 mM, MgCl_2_ (500 mM), pH 6.0, final concentration is 0.8 μM))) was added to the dnTRP_R0/R5-ΔHis protein sample (95 μl, MES buffer (100 mM, MgCl_2_ (500 mM), purified dnTRP_R0/R5-ΔHis (10.5 μM), pH 6.0)) in a glass vial (2 ml, clear robo vial, 9 mm thread, item no. VT009-1232). The vials were tightly sealed and incubated (30 °C, 250 rpm, 2 h). After chilling (5 min, on ice), the substrate **1b**, **1c**, **1d**, **1e**, **1f** or **1g** (1 μl, 200 mM stock in DMSO, [substrate]_final_ = 2.0 mM) was added. The vials were resealed and incubated (37 °C, 300 rpm, 18 h). To prepare samples for UPLC–MS analysis in the RCM reactions of **1a**, **1b**, **1c** and **1d**, methanol (900 μl, containing benzyltriethyl-ammonium bromide (200 μM) as the internal standard) was added. To prepare samples for gas chromatography–mass spectrometry analysis, EtOAc (500 μl, containing 1 mM biphenyl as the internal standard) was added to RCM samples of **1e**, EtOAc (500 μl, supplemented with [naphthalene] = 1 mM) as the internal standard) for RCM samples of **1f** and **1g**. After adding methanol or EtOAc, all vials were sealed, incubated (37 °C, 300 rpm, 30 min) and centrifuged (4 °C, 4,400*g*, 30 min). The clear supernatant (800 μl: RCM of **1a**, **1b**, **1c** and **1d**) was subjected to UPLC–MS analysis. The upper EtOAc phase (300 μl: RCM of **1e**, **1f** and **1g**) was subjected to gas chromatography–mass spectrometry analysis. Calibration curves for determining the yield/TON of cyclized products **2a**, **2b**, **2c**, **2d**, **2e**, **3e**, **2f** or **2g** are displayed in Supplementary Fig. 12.

### In cellulo RCM by Ru1·dnTRPs

To assemble the cytoplasmic **Ru1**·dnTRPs (cells harbouring an empty vector were used as negative control), freshly collected cells were gently resuspended in MES working buffer (50 mM, MgCl_2_ (100 mM), glycerol (5% (vol/vol)), pH 6.0) to a cell density around 25 g l^−1^ (wet cell weight). The cell samples (1 ml) were individually transferred into a round-bottom 24-well plate, supplemented with the cofactor **Ru1** (1, 2, 5 or 10 μl from a freshly prepared stock (1 mM in DMSO)) and incubated (15 °C, 800 rpm, 1 h) for in cellulo assembly of artificial metathase. After incubation, cells were isolated by centrifugation (4 °C, 2,600*g*, 3 min). The resulting cells were then subjected to five washing cycles, which involved cell resuspension in MES working buffer (1 ml), incubation (15 °C, 1,000 rpm, 15 min) and cell collection by centrifugation (20 °C, 2,600*g*, 3 min). The cells then were resuspended in MES working buffer (0.33 ml) at a cell density around 75 g l^−1^ (wet cell weight). The resuspended cell sample (100 μl) was aliquoted into a 96-well plate (MASTERBLOCK, 96 well, PP, 0.5 ml, V-bottom), supplemented with substrate **1a** (2.5 mM, 1 μl from a stock (250 mM in DMSO and incubated (20 °C, 300 rpm, 22 h) under sealed conditions. The subsequent steps concerning reaction quenching, sample preparation and UPLC–MS analysis were carried out as the protocol described in the Supplementary Methods. The results are summarized in Supplementary Fig. 15b.

### Engineering of dnTRP_R5 in cytoplasm of *E. coli*

The plasmid of pET-29b dnTRP_R5 was used as template for constructing of dnTRP_R5 L8X, L113X, A148X and L183X (where X represents any amino acid except cysteine and proline). The PCR amplifications were conducted in a 96-well PCR plate using the corresponding primers, Supplementary Table 4. The PCR products were first digested with DpnI (37 °C, 20 h) and then individually transformed (3 μl) into *E. coli* Top10 Chemically Competent Cells (15 μl) in a new 96-well PCR plate. The transformed cells were individually plated on LB agar (supplemented with 50 μg ml^−1^ kanamycin) and cultivated (37 °C, 20 h). The colonies with the correct sequence were cultivated in LB medium (3 ml, supplemented with 50 μg ml^−1^ kanamycin), and the plasmids were isolated by miniprep. The plasmids were then individually transformed into *E. coli* LEMO21 chemically competent cells in a 96-well PCR plate. The transformed cells were plated on LB agar plate (with 50 μg ml^−1^ kanamycin) and incubated (37 °C, 14 h). The colonies were picked and inoculated into a main culture (ZYP auto-induction medium (30 ml), kanamycin (400 μg ml^−1^), in a 250 ml baffled shaking flask) to express the corresponding TRPs. The culture was initially incubated (37 °C, 180 rpm) to an OD_600_ = 0.3–0.4, followed by further incubation (20 °C, 180 rpm, ≥18 h) to an OD_600_ ≥ 14. After expression, the cells were collected by centrifugation (4 °C, 2,600*g*, 10 min).

To perform screening of the 68 dnTRP_R5 variant library at positions L8, L113, A148 and L183 for cytoplasmic RCM, a simplified protocol was applied. In brief, the collected cells were immediately resuspended in the MES incubation buffer (50 mM, MgCl_2_ (100 mM), glycerol (5% (vol/vol)), 0.02% (wt/vol) poloxamer 188, pH 6.0) to a cell density at 25 g l^−1^ (wet cell weight). The cell samples (0.3 ml) were transferred into a 96-well plate (MASTERBLOCK, 96 well, PP, 0.5 ml, V-bottom), supplemented with the cofactor **Ru1** (0.5 μl from a freshly prepared stock (0.9 mM in DMSO), final concentration 1.5 μM) and incubated (30 °C, 1,000 rpm, 1.5 h). After incubation, the cells were obtained by centrifugation (25 °C, 2,600*g*, 5 min). The obtained cell samples were then subjected to two consecutive washing step, which consisted of cell resuspension in MES washing buffer (0.3 ml, 50 mM, MgCl_2_ (100 mM), glycerol (5% (vol/vol)), 0.02% (wt/vol) poloxamer 188, 0.0075% (vol/vol) Triton X-100, pH 6.0), incubation (30 °C, 1,000 rpm, 30 min) and cell collection by centrifugation (25 °C, 2,600*g*, 3 min). The cells were then resuspended in the MES incubation buffer at a cell density around 75 g (wet cell weight) per litre. To perform the whole-cell RCM at pH 4.2 or 5.2, cells were resuspended in NaOAc incubation buffer (0.1 ml, 50 mM, MgCl_2_ (100 mM), glycerol (5% (vol/vol)), 0.02% (wt/vol) poloxamer 188) with pH at 4.2 or 5.2. The subsequent steps of cytoplasmic RCM (1a) were conducted as described above the ‘In cellulo RCM by **Ru1**·dnTRPs’ section. A schematic representation of the screening protocol for the directed evolution of **Ru1**·dnTRP in the cytoplasm of *E. coli* is presented Supplementary Fig. 17.

For the cytoplasmic RCM using substrate **1e**, the resuspended cell sample (100 μl) was transferred into a 96-well plate (Nunc MicroWell, Nunclon Delta-Treated, flat bottom), supplemented with substrate **1e** (1 mM, 1 μl of a 100 mM stock in DMSO)), sealed with a transparent polystyrene lid and subjected to continuous fluorescence recording (excitation: 325 nm, emission: 450 nm, room temperature, Tecan Infinite M1000 PRO). The calibration curve for fluorogenic quantification was generated by supplementing and recording the fluorescence of a gradient of concentrations (5–80 μM) of the fluorescent product **3e** (Supplementary Fig. 12a) in MES buffer (100 mM, MgCl2 (500 mM), pH 6.0) or *E. coli* cell suspensions (*E. coli* cells harbour empty vector, 75 g l^−1^ wet cell weight, in MES incubation buffer) (Supplementary Fig. 18b,e).

### ICP-MS

Freshly collected cells expressing dnTRP_R0 and dnTRP_R5 (cells harbouring an empty vector were used as controls) were immediately resuspended in MES working buffer (50 mM, MgCl_2_ (100 mM), glycerol (5% (vol/vol)), pH 6.0) at a cell density of 25 g l^−1^ (wet cell weight). The resuspended cell samples (20 ml) were transferred into a Falcon tube (50 ml, polypropylene Conical Tube, 30 mm × 115 mm style) and supplemented with the cofactor **Ru1** (40 μl from a freshly prepared stock (1 mM in DMSO), final concentration is 2 μM). The samples were incubated (20 °C, 300 rpm, 1 h), after which the cells were collected by centrifugation (4 °C, 2,600*g*, 10 min). The resulting cells were then subjected to five washing step cycles, which involved cell resuspension in MES working buffer (20 ml), incubation (20 °C, 300 rpm, 15 min) and cell collection by centrifugation (4 °C, 2,600*g*, 8 min). The cells were then frozen (−20 °C, 22 h), thawed (37 °C, 300 rpm, 30 min) and resuspended in a modified MES working buffer (5 ml, 50 mM, MgCl_2_ (500 mM), glycerol (5% (vol/vol)), pH 6.0) for cell fragmentation. A schematic representation of the steps for the preparation of cell fragments is summarized in Supplementary Fig. 16a. In brief, the cells were lysed on ice by sonication (1 s on–off, 60% amplitude, 5 min). The clear supernatant A (hereafter refers to as clear supernatant obtained by cell lysis of sonication) and cell pellet A (referred to as cell debris) were obtained by centrifugation (4 °C, 12,000*g*, 10 min). The clear supernatant A was further processed with an ultracentrifugation (4 °C, 87,000*g*, 2.5 h) to afford the clear supernatant B (hereafter referred to cytoplasmic fragment) and cell pellet B (hereafter referred to membranous fragments). Cell pellet A and cell pellet B were fully resuspended in MES working buffer (5 ml). The contents of dnTRP_R5 in the prepared fragments were analysed by SDS–polyacrylamide gel electrophoresis, Supplementary Fig. 16b. For ICP-MS, the samples of clear supernatant B from three independently performed experiments were pooled, aliquoted and subjected to ICP-MS analysis.

### Reporting summary

Further information on research design is available in the Nature Portfolio Reporting Summary linked to this article.

## Supplementary information

Supplementary Information Supplementary Figs. 1–20, Tables 1–5 and methods.

Reporting Summary

Supplementary Fig. 3 Statistical source data.

Supplementary Fig. 4 Statistical source data.

Supplementary Fig. 5 Statistical source data.

Supplementary Fig. 6 Statistical source data.

Supplementary Fig. 7 Statistical source data.

Supplementary Fig. 8 Statistical source data.

Supplementary Fig. 10 Statistical source data.

Supplementary Fig. 11 Statistical source data.

Supplementary Fig. 12 Statistical source data.

Supplementary Fig. 15 Statistical source data.

Supplementary Fig. 17 Statistical source data.

Supplementary Fig. 18 Statistical source data.

Supplementary Fig. 19 Statistical source data.

Supplementary Fig. 20 Statistical source data.

## Source data

Source Data Fig. 2 Statistical source data.

Source Data Fig. 3 Statistical source data.

Source Data Fig. 5 Statistical source data.

## Data Availability

The original materials, methods and data underlying the findings of this study are available within the Article and its Supplementary Information. The PDB accession codes of apo dnTRP_R0-ΔHis, **Ru1**·R0-ΔHis and **Ru1**·R5-ΔHis are 9GVF, 8S6P and 9H3C, respectively. All other data are available from the authors upon request. Source data are provided with this paper.

## Code availability

The source code for the de novo scaffold design is available via GitHub at https://github.com/ikalvet/denovo_metathase_design.
