# Supplementary material for: De novo design and evolution of an artificial metathase for cytoplasmic olefin metathesis
Source: Nat Catal. 2025 Nov 3;8(11):1208–19. doi: 10.1038/s41929-025-01436-0 (PMC12638248; doi:10.1038/s41929-025-01436-0)
Supplement: Supplementary file 1 — Supplementary Figs. 1–20, Tables 1–5 and methods. [file 41929_2025_1436_MOESM1_ESM.pdf]

nature catalysis

Supplementary information https://doi.org/10.1038/s41929-025-01436-0

De novo design and evolution of an artificial metathase for cytoplasmic olefin metathesis

In the format provided by the authors and unedited

Supplementary Information

Table of Contents

Supplementary Methods **4**

Chemicals and Materials 4

Chromatographic methods (LC-MS and GC-MS) 4

Synthesis of the metathesis cofactor **Ru1**... 5

Synthesis of ring-closing metathesis substrates and products 12

Computational design of **Ru1**·dnTRP 14

Expression of dnTRP in shake flasks and 96-well microtiter plates..16

Circular dichroism (CD) spectroscopy of dnTRP..17

Determination of the binding affinity (K_D) of **Ru1** to dnTRPs 17

Native mass spectrometry of **Ru1**·R0...17

Size-exclusion chromatography of **Ru1**·R0 18

Optimization **Ru1**·dnTRPs-based RCM in cell-free extract (CFE)..18

Characterizations of evolved **Ru1**·dnTRP variants 18

X-ray study of **Ru1**·dnTRPs...19

Cell viability: colony formation assay 20

Supplementary Figures **21**

Supplementary Fig. 1. Procedure adopted for the design of the cofactor **Ru1** binding site with Rosetta FastDesign. 21

Supplementary Fig. 2. Expression and purification of dnTRP designs. 22

Supplementary Fig. 3. Circular dichroism (CD) spectra of dnTRP_18 at different pHs (a) and following incubation at 98 °C (b). 23

Supplementary Fig. 4. Tryptophan fluorescence-quenching assay for the determination of the binding affinity of **Ru1**·to dnTRPs. 24

Supplementary Fig. 5. Native mass spectrometry and size-exclusion chromatography of **Ru1**·R0. 25

Supplementary Fig. 6. Optimization of ring-closing metathesis in cell-free extract (CFE).......26

Supplementary Fig. 7. Expression and determination of the concentration of dnTRP_18 in the cell-free extract in a 96-well plate format. 27

Supplementary Fig. 8. Directed evolution of **Ru1**·dnTRP using cell-free extracts at pH 4.2. ..28

Supplementary Fig. 9. Removal of N-terminal His-tag from dnTRPs and determination of the corresponding K_D of various Ru1·dnTRPs.. 29

Supplementary Information

Supplementary Fig. 10. Binding affinity (to **Ru1**) and expression of dnTRP-Δhis in cytoplasm of *E. coli*. 30
Supplementary Fig. 11. Activity of **Ru1**·dnTRPs at different temperatures and in presence of varying concentrations of glutathione (GSH)....31
Supplementary Fig. 12. Comparison of RCM performance for different substrates catalyzed by **Ru1** and **Ru1**·dnTRPs-ΔHis ArMs....32
Supplementary Fig. 13. Structural characterization of **Ru1**·dnTRPs....33
Supplementary Fig. 14. Structure prediction analysis of **Ru1**·dnTRPs....35
Supplementary Fig. 15. Expression of dnTRPs and optimization of [**Ru1**] concentration for the assembly of cytoplasmic **Ru1**·dnTRPs and corresponding cell viability determination....36
Supplementary Fig. 16. Fragmentation of *E. coli* cells for inductively coupled plasma mass spectrometry (ICP-MS) determination of [Ru] concentration....37
Supplementary Fig. 17. Screening dnTRP_R5 L8X, L113X, A148X and L183X variants at pH 6.0 for improved RCM activity in whole cells with the substrate 1a....38
Supplementary Fig. 18. Ring-closing metathesis of diene 1e using **Ru1**·dnTRPs....39
Supplementary Fig. 19. Binding affinity and turnover numbers of variants **Ru1**·R5_A148I/L183M-ΔHis and **Ru1**·R5_A148V/L183M-ΔHis....40
Supplementary Fig. 20. Turnover numbers of **Ru1**·dnTRP-Δhis (using purified protein) in RCM of **1a**, **1e**, **1f**, and **1g** in absence or presence of thiols....41
Supplementary Tables....42
Supplementary Table 1 Publication reporting *in (or on)-cellulo* new-to-nature reactions catalyzed by artificial metalloenzymes....42
Supplementary Table 2 Primers used for site-directed (SDM) and site-saturation mutagenesis (SSM) PCRs....43
Supplementary Table 3 Primers used for error-prone PCR and fragment shuffling....45
Supplementary Table 4 Primers used for generation of L8X, L113X, A148X, L183X and their corresponding recombined variants (X represents any amino acid residues except cysteine and proline). 45
Supplementary Table 5. Statistical data for the X-ray crystal structures of apo dnTRP_R0-Δhis, **Ru1**·R0-Δhis and **Ru1**·R5-Δhis. The data have been deposited under PDB: 9GVF, 8S6P, and 9H3C, respectively....48
Supplementary Appendix....49

Supplementary Information

DNA and corresponding protein sequence of the dnTRP designs. 49
Uncropped scans of all blots and gels 53
Supplementary References 58

Supplementary Information

Supplementary Methods

Chemicals and Materials

All chemicals were purchased from commercial sources and used without further purification. Compound **1d** (98%), **2e** (99%), **3e** (98%), methanol (99%), trifluoroacetic acid (TFA, 99%), and benzyltriethyl-ammonium bromide were purchased from Sigma Aldrich. Dimethyl sulfoxide (DMSO, 99%), $MgCl_2$, $MnCl_2$, KH_2PO_4, K_2HPO_4, Na_2HPO_4, and NaH_2PO_4 were purchased from Acros Organics. The substrates **1a**, **1b**, **1e**, **1f**, and **1g** their corresponding cyclized product **2a**, **2b**, **2f**, and **2g** were synthesized as previously described[1-3]. The synthesis of **1c**, **1e**, **2c**, and **2d** is described below in this Supplementary Information.

Phusion high fidelity DNA polymerase, Taq polymerase 2X Master Mix, NEBuilder® HiFi DNA Assembly Master Mix, NEBridge® Golden Gate Assembly Kit (*BsaI-HF® v2*), *Dpn*I restriction enzyme, the T4 DNA ligase, gel extraction-kit, plasmid miniprep-kit, and *E. coli* LEMO21 chemically-competent cells were purchased from New England BioLabs (NEB). The oligonucleotides for polymerase chain reaction (PCR) were ordered from Microsynth AG. The DNA sequences for the 21 dnTRPs (in pET-29b vector) were obtained as sub-cloned plasmids from Integrated DNA Technologies (IDT).

Chromatographic methods (LC-MS and GC-MS)

The cyclized products **2a**, **2b**, **2c**, **2d** and **3e** were quantified by UPLC-MS. After ring-closing metathesis (RCM), samples were diluted with four or nine reaction volumes of methanol (with [benzyltriethyl-ammonium bromide] = 200 µM used as the internal standard). Analyses were performed on a Waters Acquity UPLC system using an Acquity UPLC CSH C18 column (2.1 x 50mm, 1.7 µm particles). The flow rate was maintained at 0.5 mL min^{-1}. The elution program began with 75% water (0.1% FA) and 25% acetonitrile (ACN, 0.1% FA), held from 0-0.5 min, followed by a linear gradient reaching 98% ACN (0.1% FA) and 2% water (0.1% FA) from 0.5 to 3.2 min. This composition was held until 3.5 min, then gradually returned to 75% water and 25% ACN by 3.8 min, and remained constant until 4 min.

GC–MS analysis was performed to quantify the cyclized products **2e**, **2f**, and **2g**. The products were extracted from the reaction mixture with ethyl acetate (EtOAc) and analyzed using a Shimadzu GC-2010 Plus system coupled with a GC-MS-QP2020 detector (column: Agilent HP-5 30 m × 0.25 mm × 0.25 µm). The temperature program was initiated at 40 °C, held for 0.5 min, then increased to 300 °C at 40 $°C \cdot min^{-1}$, and maintained at 300 °C for 3 min. Cyclized products **2e**, **2f**, and **2g** were detected in the total ion chromatogram (TIC) and quantified using the selective ion monitoring mode (SIM) at m/z = 128. Internal standards ([Biphenyl] = 1 mM for cyclized product **2e**) and ([**2e**] = 1 mM for cyclized products **2f** and **2g**) were used for the calibration curves and to determine the yield of the reaction, Supplementary Fig. 12.

Synthesis of the metathesis cofactor Ru1

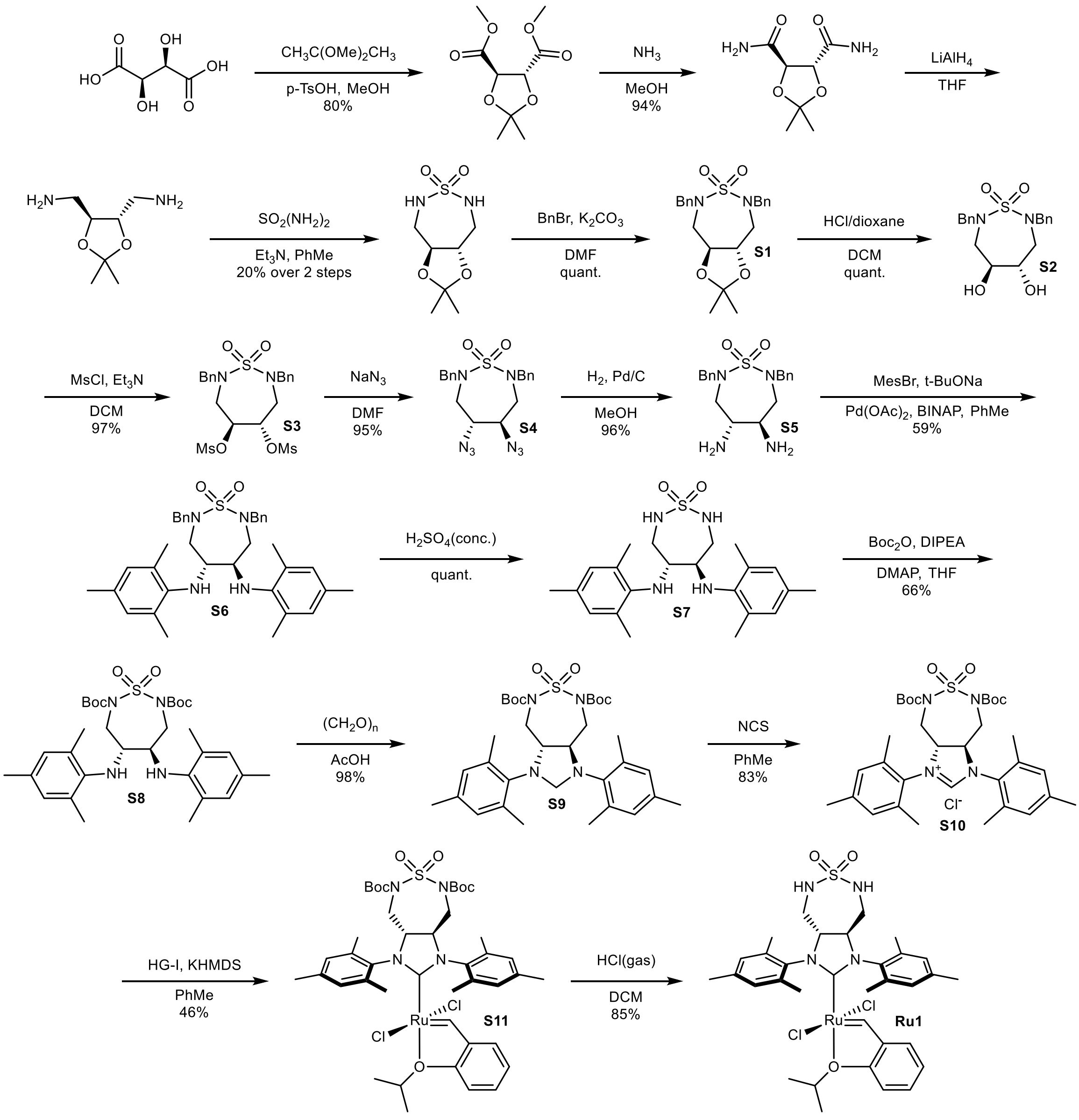

(3a*S*,8a*S*)-5,7-Dibenzyl-2,2-dimethylhexahydro-[1,3]dioxolo[4,5-d] [1,2,7]thiadiazepine 6,6-dioxide (S1)

(3a*S*,8a*S*)-5,7-Dibenzyl-2,2-dimethylhexahydro-[1,3]dioxolo[4,5-*d*][1,2,7]thiadiazepine 6,6-dioxide **S1** was synthesized according to a described protocol[4].

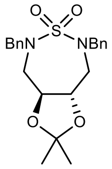

S1

^{1}H NMR (500 MHz, $CDCl_3$) δ 7.40 – 7.28 (m, 10H), 4.47 (d, *J* = 14.5 Hz, 2H), 4.36 (d, *J* = 14.5 Hz, 2H), 4.25 – 4.12 (m, 2H), 3.44 (dd, *J* = 12.9, 4.2 Hz, 2H), 3.05 – 2.94 (m, 2H), 1.35 (s, 6H).

HRMS (ESI positive mode, m/z): calculated for $C_{21}H_{27}N_2O_4S$ $[M+H]^+$ 403.1683; found 403.1683.

(4*S*,5*S*)-2,7-Dibenzyl-4,5-dihydroxy-1,2,7-thiadiazepane 1,1-dioxide (S2)

(4*S*,5*S*)-2,7-Dibenzyl-4,5-dihydroxy-1,2,7-thiadiazepane 1,1-dioxide **S2** was synthesized according to a described protocol[4].

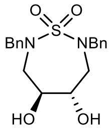

S2

^{1}H NMR (500 MHz, $CDCl_3$) δ 7.47 – 7.28 (m, 10H), 4.65 (d, *J* = 15.3 Hz, 2H), 4.46 (d, *J* = 15.3 Hz, 2H), 3.58 – 3.45 (m, 2H), 3.38 (dd, *J* = 15.1, 8.8 Hz, 2H), 3.16 (dd, *J* = 15.1, 2.6 Hz, 2H), 2.08 (d, *J* = 3.7 Hz, 2H).

(4*S*,5*S*)-2,7-Dibenzyl-1,1-dioxido-1,2,7-thiadiazepane-4,5-diyl dimethanesulfonate (S3)

The crude diol **S2** (7.25 g, 20 mmol) was dissolved in anhydrous dichloromethane (150 mL). Triethylamine (11.05 mL, 80 mmol) was added dropwise, followed by methanesulfonyl chloride (3.5 mL, 45 mmol) which was also added dropwise to the reaction mixture at 0 °C. After completion of the addition, the mixture was stirred at room temperature for 12 h. Then, the mixture was washed with water, saturated sodium bicarbonate and saturated brine, dried over anhydrous sodium sulfate, and filtered. The volatiles were removed under reduced pressure to give (4*S*,5*S*)-2,7-dibenzyl-1,1-dioxido-1,2,7-thiadiazepane-4,5-diyl dimethanesulfonate **S3** as a yellow foam (10.05 g, 97%).

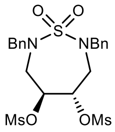

S3

^{1}H NMR (500 MHz, $CDCl_3$) δ 7.47 – 7.33 (m, 10H), 4.59 (d, *J* = 15.1 Hz, 2H), 4.52 (d, *J* = 15.1 Hz, 2H), 4.48 – 4.39 (m, 2H), 3.61 (dd, *J* = 15.5, 9.4 Hz, 2H), 3.43 (dd, *J* = 15.6, 3.0 Hz, 2H), 2.92 (s, 6H).

^{13}C NMR (126 MHz, $CDCl_3$) δ 135.53, 129.24, 129.00, 128.70, 76.22, 52.85, 46.38, 38.65.
HRMS (ESI positive mode, m/z): calculated for $C_{20}H_{27}N_2O_8S_3$ $[M+H]^+$ 519.0918; found 519.0924.

(4*R*,5*R*)-4,5-Diazido-2,7-dibenzyl-1,2,7-thiadiazepane 1,1-dioxide (S4)

The bis-mesylate **S3** (5 g, 9.6 mmol) was dissolved in N,N-dimethylformamide (80 mL), then sodium azide (2.5 g, 38.5 mmol) was added, the reaction mixture was heated to 80 °C and stirred for 16 h. Then, water (200 mL) was added, the mixture was extracted with ethyl acetate (150 mL×3), and the combined organic phase was washed with water (200 mL×2) and saturated brine (200 mL) successively. It was then dried over anhydrous sodium sulfate, filtered, and the organic solvents were removed under reduced pressure. The crude product was purified by silica gel column chromatography (3-5% EtOAc in cyclohexane) to afford (4*R*,5*R*)-4,5-diazido-2,7-dibenzyl-1,2,7-thiadiazepane 1,1-dioxide **S4** as a pale-yellow oil (3.76 g, 95%).

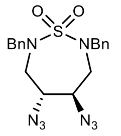

S4

^{1}H NMR (500 MHz, $CDCl_3$) δ 7.45 – 7.33 (m, 10H), 4.56 (d, *J* = 15.0 Hz, 2H), 4.51 (d, *J* = 15.0 Hz, 2H), 3.47 (dt, *J* = 16.3, 8.2 Hz, 2H), 3.10 (dd, *J* = 15.5, 2.7 Hz, 2H), 3.00 – 2.92 (m, 2H).
^{13}C NMR (126 MHz, $CDCl_3$) δ 135.73, 129.32, 128.74, 128.72, 62.76, 52.68, 46.95.
HRMS (ESI positive mode, m/z): calculated for $C_{18}H_{20}N_8NaO_2S$ $[M+Na]^+$ 435.1315; found 435.1322.

(4*R*,5*R*)-4,5-Diamino-2,7-dibenzyl-1,2,7-thiadiazepane 1,1-dioxide (S5)

A solution containing the diazide **S4** (4.5 g, 10.9 mmol) in methanol (50 ml) was added to a stirred suspension of 10% Pd on charcoal (1.65 g, 0.14 eq.) in methanol (100 mL). The mixture was hydrogenated at atmospheric pressure and room temperature for 6 h. The catalyst was removed by filtration and washed with methanol. The solvent was removed under reduced pressure to afford (4*R*,5*R*)-4,5-diamino-2,7-dibenzyl-1,2,7-thiadiazepane 1,1-dioxide **S5** as a colourless oil (3.76 g, 96%) and used without further purification for the next step.

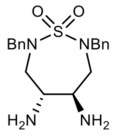

S5

^{1}H NMR (500 MHz, $CDCl_3$) δ 7.44 – 7.29 (m, 10H), 4.73 (d, *J* = 15.5 Hz, 2H), 4.37 (d, *J* = 15.5 Hz, 2H), 3.45 – 3.23 (m, 2H), 2.87 (dd, *J* = 15.3, 2.6 Hz, 2H), 2.52 – 2.31 (m, 2H).

^{13}C NMR (126 MHz, $CDCl_3$) δ 136.77, 128.98, 128.29, 128.07, 55.41, 52.50, 50.88.
HRMS (ESI positive mode, m/z): calculated for $C_{18}H_{25}N_4O_2S$ $[M+H]^+$ 361.1689; found 361.1693.

(4*R*,5*R*)-2,7-Dibenzyl-4,5-bis(mesitylamino)-1,2,7-thiadiazepane 1,1-dioxide (S6)

Under an inert atmosphere, tris(dibenzylideneacetone) dipalladium (0) (1.40 g, 1.5 mmol), BINAP (1.86 g, 3 mmol), and sodium *t*-butoxide (4.20 g, 43.7 mmol) were added to toluene (300 mL) and stirred for 20 min. The diamine **S5** (3.61 g, 10 mmol) and 2-bromomesitylene (7.96 g, 40 mmol) were then added and the solution was heated at 100 °C for 16 h. The solution was then cooled to room temperature and concentrated under reduced pressure. The residue was purified by silica gel column chromatography (10% EtOAc in cyclohexane) to afford (4*R*,5*R*)-2,7-dibenzyl-4,5-bis(mesitylamino)-1,2,7-thiadiazepane 1,1-dioxide **S6** as a white foam (3.53 g, 59%).

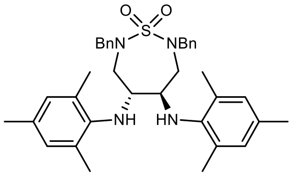

S6

^{1}H NMR (500 MHz, $CDCl_3$) δ 7.28 – 7.24 (m, 2H), 7.23 – 7.17 (m, 4H), 7.02 – 6.96 (m, 4H), 6.80 (s, 4H), 4.33 (d, *J* = 14.6 Hz, 2H), 4.24 (d, *J* = 14.6 Hz, 2H), 3.38 (dd, *J* = 14.9, 9.7 Hz, 2H), 3.03 (s, 2H), 2.88 – 2.74 (m, 4H), 2.29 (s, 6H), 2.07 (s, 12H).
^{13}C NMR (126 MHz, $CDCl_3$) δ 139.73, 136.41, 133.12, 132.42, 129.80, 129.03, 128.77, 127.97, 60.22, 52.43, 47.26, 27.05, 20.79, 18.80.
HRMS (ESI positive mode, m/z): calculated for $C_{36}H_{45}N_4O_2S$ $[M+H]^+$ 597.3269; found 597.3258.

(4*R*,5*R*)-4,5-Bis(mesitylamino)-1,2,7-thiadiazepane 1,1-dioxide (S7)

The benzyl-protected sulfamide **S6** (3.53 g, 5.9 mmol) was crushed and mixed with concentrated H_2SO_4 (40 mL). The reaction mixture was vigorously stirred for 1 h and then crushed ice (200 g) was added at 0 °C. Then, [NaOH] = 2 mM was added dropwise to reach pH=8. The mixture was extracted with DCM (100 mL×6), and the organic phase was washed with water (100 mL) and saturated brine (200 mL) successively, dried over anhydrous sodium sulfate, and filtered. The organic solvent was removed under reduced pressure to afford (4*R*,5*R*)-4,5-bis(mesitylamino)-1,2,7-thiadiazepane 1,1-dioxide **S7** as an off-white solid (2.43 g, quant.). The crude product was used in the next step without further purification.

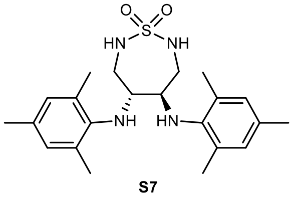

S7

^{1}H NMR (500 MHz, Acetone-d_6) δ 6.75 (s, 4H), 6.30 (t, *J* = 5.2 Hz, 2H), 3.88 (d, *J* = 8.0 Hz, 2H), 3.44 (dd, *J* = 12.8, 6.0 Hz, 2H), 3.31 – 3.18 (m, 4H), 2.17 (s, 6H), 2.12 (s, 12H).

^{13}C NMR (126 MHz, Acetone-d_6) δ 141.97, 141.93, 131.95, 131.28, 131.19, 130.30, 60.87, 60.77, 43.06, 42.97, 20.60, 19.03, 19.02.
HRMS (ESI positive mode, m/z): calculated for $C_{22}H_{33}N_4O_2S$ $[M+H]^+$ 417.2326; found 417.2319.

Di-tert-butyl (4*R*,5*R*)-4,5-bis(mesitylamino)-1,2,7-thiadiazepane-2,7-dicarboxylate 1,1-dioxide (S8)

The deprotected sulfamide **S7** (2.43 g, 5.9 mmol), Et_3N (5.0 mL, 36 mmol), and DMAP (216 mg, 1.77 mmol) were dissolved in chloroform (250 mL) and cooled to 0 °C. Then, Boc_2O (3.86 g, 17.7 mmol) was added portionwise, and the resulting mixture was stirred at 0 °C for 30 min, and then at room temperature for 3 h. The reaction mixture was concentrated under reduced pressure and purified by silica gel column chromatography (15% EtOAc in cyclohexane) to afford di-*tert*-butyl (4*R*,5*R*)-4,5-bis(mesitylamino)-1,2,7-thiadiazepane-2,7-dicarboxylate 1,1-dioxide **S8** as a white foam (2.4 g, 66%).

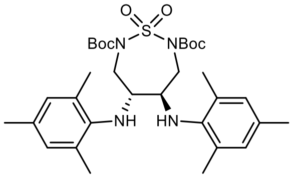

S8

^1H NMR (500 MHz, $CDCl_3$) δ 6.82 (s, 4H), 4.00 (dd, J = 15.1, 1.9 Hz, 2H), 3.73 – 3.67 (m, 2H), 3.48 (s, 2H), 3.42 – 3.31 (m, 2H), 2.23 (s, 18H), 1.39 (s, 18H).
^{13}C NMR (126 MHz, $CDCl_3$) δ 150.32, 139.68, 132.99, 131.84, 129.95, 85.28, 60.40, 48.24, 27.85, 20.69, 19.05.
HRMS (ESI positive mode, m/z): calculated for $C_{32}H_{49}N_4O_6S$ $[M+H]^+$ 617.3370; found 617.3367.

Di-*tert*-butyl (3a*R*,8a*R*)-1,3-dimesitylhexahydro-1*H*-imidazo[4,5-*d*][1,2,7]thiadiazepine-5,7-dicarboxylate 6,6-dioxide (S9)

The diamine **S8** (2.4 g, 3.9 mmol) was dissolved in acetic acid (100 mL), and paraformaldehyde (0.40 g, 13.3 mmol) was added. The reaction was stirred until the paraformaldehyde had dissolved. Then, the acetic acid was removed under reduced pressure and the resulting crude material was purified by column chromatography (10% EtOAc in cyclohexane) to give di-*tert*-butyl (3a*R*,8a*R*)-1,3-dimesitylhexahydro-1*H*-imidazo[4,5-*d*][1,2,7]thiadiazepine-5,7-dicarboxylate 6,6-dioxide **S9** as a white foam (2.41 g, 98%).

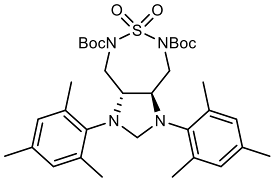

S9

^{1}H NMR (500 MHz, $CDCl_3$) δ 6.88 (s, 2H), 6.84 (s, 2H), 4.38 (s, 2H), 4.11 – 4.03 (m, 2H), 3.82 (dd, *J* = 14.0, 2.8 Hz, 2H), 3.61 (dt, *J* = 14.0, 7.3 Hz, 2H), 2.40 (s, 6H), 2.33 (s, 6H), 2.25 (s, 6H), 1.43 (s, 18H).
^{13}C NMR (126 MHz, $CDCl_3$) δ 150.78, 139.84, 137.35, 137.24, 136.48, 130.98, 129.32, 84.77, 70.94, 66.32, 48.69, 27.92, 27.06, 20.92, 19.16, 19.16.
HRMS (ESI positive mode, m/z): calculated for $C_{33}H_{49}N_4O_6S$ $[M+H]^+$ 629.3358; found 629.3367.

(3a*R*,8a*R*)-5,7-Bis(*tert*-butoxycarbonyl)-1,3-dimesityl-3a,4,5,7,8,8a-hexahydro-1*H*-imidazo[4,5-*d*][1,2,7]thiadiazepin-3-ium 6,6-dioxide chloride (S10).

The imidazolidine **S9** (200 mg, 0.32 mmol) was dissolved in toluene (20 mL). N-chlorosuccinimide (45 mg, 0.34 mmol) was added, and the reaction mixture was stirred at room temperature for 5 h (depending on the quality of NCS, the stirring may be extended to ensure full conversion). After completion, toluene was removed under reduced pressure and the resulting foam was dissolved in a minimal amount of DCM. This solution was added dropwise to vigorously a stirred mixture of hexane (20 mL) and diethyl ether (20 mL) to separate product from succinimide. The solids were collected by vacuum filtration, washed with an hexane-ether mixture, and dried under vacuum to afford (3a*R*,8a*R*)-5,7-bis(*tert*-butoxycarbonyl)-1,3-dimesityl-3a,4,5,7,8,8a-hexahydro-1*H*-imidazo[4,5-*d*][1,2,7]thiadiazepin-3-ium 6,6-dioxide chloride **S10** as a white powder (176 mg, 83%).

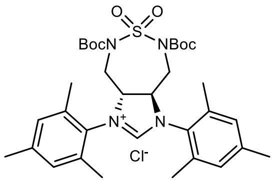

S10

^{1}H NMR (500 MHz, $CDCl_3$) δ 10.92 (s, 1H), 7.01 (s, 2H), 6.98 (s, 2H), 4.81 (s, 2H), 4.01 (d, *J* = 14.1 Hz, 2H), 3.97 – 3.89 (m, 2H), 2.46 (s, 6H), 2.37 (s, 6H), 2.29 (s, 6H), 1.46 (s, 18H).
^{13}C NMR (126 MHz, $CDCl_3$) δ 162.07, 149.87, 141.45, 135.98, 134.72, 130.95, 130.68, 127.82, 87.02, 67.73, 45.09, 27.84, 21.20, 19.42, 18.60.
HRMS (ESI positive mode, m/z): calculated for $C_{33}H_{47}N_4O_6S$ $[M-Cl]^+$ 627.3218; found 627.3211.

Dichloro((3a*R*,8a*R*)-5,7-bis(*tert*-butoxycarbonyl)-1,3-dimesityl-6,6-dioxidooctahydro-2*H*-imidazo[4,5-*d*][1,2,7]thiadiazepin-2-ylidene)(2-isopropoxybenzylidene)ruthenium(II) (S11).

The dihydroimidazolium salt **S10** (40 mg, 0.06 mmol), KHMDS (24 mg, 0.12 mmol), and the Hoveyda-Grubbs 1st generation catalyst (72 mg, 0.12 mmol) were suspended in dry and degassed toluene (10 mL). The reaction mixture was heated to 80 °C for 5 h. The solvent was evaporated and the crude material was purified by column chromatography (10% EtOAc in cyclohexane) to afford dichloro((3a*R*,8a*R*)-5,7-bis(*tert*-butoxycarbonyl)-1,3-dimesityl-6,6-dioxidooctahydro-2*H*-imidazo[4,5-*d*][1,2,7]thiadiazepin-2-ylidene)(2-isopropoxybenzylidene)ruthenium(II) **S11** as a green solid (26 mg, 46%).

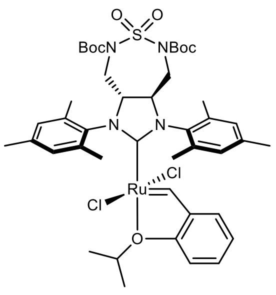

S11

^1H NMR (500 MHz, CD_2Cl_2) δ 16.20 (d, *J* = 0.8 Hz, 1H), 7.56 (dt, *J* = 8.4, 4.5 Hz, 1H), 7.13 (s, 2H), 7.08 (s, 2H), 6.92 (d, *J* = 4.3 Hz, 2H), 6.85 (d, *J* = 8.3 Hz, 1H), 4.89 (hept, *J* = 6.1 Hz, 1H), 4.71 (brs, 2H), 4.09 – 3.77 (m, 4H), 2.42 (brs, 18H), 1.43 (s, 18H), 1.20 (dd, *J* = 6.1, 3.0 Hz, 6H).

HRMS (ESI positive mode, m/z): calculated for $C_{43}H_{58}N_4O_7RuS$ $[M-2Cl]^{2+}$ 438.1536; found 438.1535.

Dichloro((3a*R*,8a*R*)-1,3-dimesityl-6,6-dioxidooctahydro-2*H*-imidazo[4,5-*d*][1,2,7]thiadiazepin-2-ylidene)(2-isopropoxybenzylidene)ruthenium(II) (Ru1).

The boc-protected sulfamide **S11** (26 mg, 0.027 mmol) was dissolved in CH_2Cl_2 (5 mL) in a Schlenk tube, and HCl gas was purged through the solution for 3 h at room temperature. Gaseous HCl was generated by dropwise addition of concentrated H_2SO_4 to NH_4Cl. The solution was stirred for an additional 1 h at room temperature. Upon completion of the reaction as revealed by TLC (30% EtOAc in cyclohexane), the solvent was evaporated under reduced pressure, and the crude material was purified by column chromatography (30% EtOAc in cyclohexane) to afford dichloro((3a*R*,8a*R*)-1,3-dimesityl-6,6-dioxidooctahydro-2*H*-imidazo[4,5-*d*][1,2,7]thiadiazepin-2-ylidene)(2-isopropoxybenzylidene)ruthenium(II) **Ru1** as a green solid (17.4 mg, 85%).

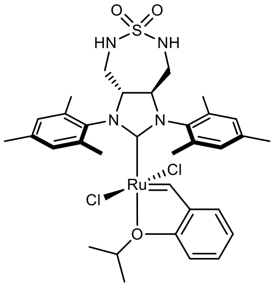

Ru1

^1H NMR (500 MHz, CD_2Cl_2) δ 16.23 (d, *J* = 0.8 Hz, 1H), 7.56 (ddd, *J* = 8.4, 5.4, 3.6 Hz, 1H), 7.11 (s, 2H), 7.08 (s, 2H), 6.95 – 6.87 (m, 2H), 6.84 (d, *J* = 8.3 Hz, 1H), 4.94 – 4.76 (m, 3H), 4.60 (t, *J* = 5.6 Hz, 2H), 3.32 (s, 4H), 2.41 (s, 18H), 1.19 (dd, *J* = 6.1, 3.5 Hz, 6H).
^{13}C NMR (126 MHz, CD_2Cl_2) δ 218.50, 152.54, 145.46, 139.98, 130.45, 130.07, 122.86, 122.73, 113.43, 75.80, 43.96, 21.25, 21.21, 18.03.
HRMS (ESI positive mode, m/z): calculated for $C_{33}H_{42}N_4O_3RuS$ $[M-2Cl]^{2+}$ 338.1009; found 338.1009. for $C_{33}H_{42}ClN_4O_3RuS$ [M-Cl]+ 711.1716; found 711.1709.

Synthesis of ring-closing metathesis substrates and products

Substrates **1a**[5], **1c**[6-8], **1f**[2], and **1g**[2] were synthesized following reported procedures. The diene **1d** was purchased from Sigma Aldrich.

Diene **1a** was prepared according to the general procedure[5]. 94% yield. ^{1}H NMR (500 MHz, $CDCl_3$) δ 7.70 (d, J = 8.2 Hz, 2H), 7.29 (d, J = 8.0 Hz, 2H), 5.61 (ddt, J = 16.2, 9.7, 6.3 Hz, 2H), 5.14 (ddt, J = 13.8, 2.7, 1.5 Hz, 4H), 3.80 (dd, J = 6.3, 1.5 Hz, 4H), 2.43 (s, 3H).

Diene **1c** was synthesized according to the reported procedure[6-8]. 32% yield over three steps. ^{1}H NMR (500 MHz, $CDCl_3$) δ 7.70 (d, *J* = 7.5 Hz, 2H), 7.26 (d, *J* = 7.5 Hz, 1H), 5.77 (dddt, *J* = 17.5, 10.2, 6.2, 1.3 Hz, 2H), 5.23 – 5.00 (m, 3H), 4.96 (ddt, *J* = 6.0, 4.5, 1.4 Hz, 0H), 3.79 (dq, *J* = 6.3, 1.3 Hz, 1H), 2.41 (s, 2H).

Diene **1f** was prepared following the reported procedure[2]. 94% yield. ^{1}H NMR (500 MHz, $CDCl_3$) δ 5.66 (ddt, *J* = 17.0, 10.3, 7.4 Hz, 2H), 5.15 – 5.03 (m, 4H), 4.18 (q, *J* = 7.1 Hz, 4H), 2.64 (dt, *J* = 7.3, 1.2 Hz, 4H), 1.24 (t, *J* = 7.1 Hz, 6H).

Substrate **1g** was prepared following the reported procedure[2]. 60% yield. ^{1}H NMR (500 MHz, $CDCl_3$) δ 7.62 – 7.55 (m, 4H), 7.35 – 7.29 (m, 4H), 7.28 – 7.23 (m, 2H), 6.00 (ddt, *J* = 17.2, 10.4, 5.2 Hz, 1H), 5.37 (dq, *J* = 17.2, 1.8 Hz, 1H), 5.17 (dq, *J* = 10.5, 1.5 Hz, 1H), 4.04 (dt, *J* = 5.2, 1.6 Hz, 2H), 2.89 (s, 1H).

Substrate **1e** was prepared according to the following scheme:

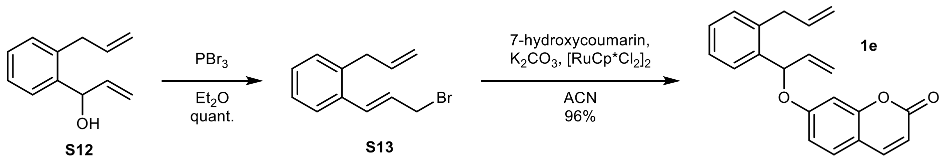

1-(2-Allylphenyl)prop-2-en-1-ol **S12** was obtained according to a reported procedure[3]. ^{1}H NMR (500 MHz, $CDCl_3$) δ 7.56 – 7.43 (m, 1H), 7.33 – 7.21 (m, 2H), 7.21 – 7.13 (m, 1H), 6.17 – 5.92 (m, 2H), 5.47 (td, *J* = 5.4, 4.7, 1.8 Hz, 1H), 5.35 (dt, *J* = 17.2, 1.5 Hz, 1H), 5.22 (dt, *J* = 10.4, 1.5 Hz, 1H), 5.09 (dq, *J* = 10.1, 1.6 Hz, 1H), 5.00 (dq, *J* = 17.1, 1.8 Hz, 1H), 3.50 (d, *J* = 6.2 Hz, 2H), 1.88 (d, *J* = 4.0 Hz, 1H).

To a stirred solution of the alcohol **S12** (174 mg, 1 mmol) in diethyl ether (2.5 mL), a solution of PBr_3 (135 mg, 0.5 mmol) in diethyl ether (1 mL) was added dropwise at 0 °C. The reaction mixture was stirred for 1 h, after which, a saturated solution of $NaHCO_3$ (1 mL) was added. The organic phase was separated, washed with brine (2 mL), dried over anhydrous sodium sulfate, and filtered. The organic solvent was removed under reduced pressure to afford (*E*)-1-allyl-2-(3-bromoprop-1-en-1-yl)benzene **S13** as a pale-yellow oil (237 mg, quant.). ^{1}H NMR (500 MHz, $CDCl_3$) δ 7.53 – 7.43 (m, 1H), 7.24 – 7.12 (m, 3H), 6.88 (d, *J* = 15.4 Hz, 1H), 6.29 (dt, *J* = 15.4, 7.8 Hz, 1H), 5.95 (ddt, *J* = 16.3, 10.1, 6.2 Hz, 1H), 5.08 (dt, *J* = 10.1, 1.6 Hz, 1H), 4.97 (dq, *J* = 17.1, 1.7 Hz, 1H), 4.17 (dd, *J* = 7.8, 0.9 Hz, 2H), 3.45 (d, *J* = 6.1 Hz, 2H).

The bromide **S13** (237 mg, 1 mmol), 7-hydroxy-2*H*-chromen-2-one (243 mg, 1.5 mmol), $[RuCp^*Cl_2]_2$ (60 mg, 0.1 mmol), and K_2CO_3 (350 mg, 2.5 mmol) were suspended in dry and degassed ACN (10 mL) and stirred for 3 h. The reaction mixture was centrifuged, and the supernatant was concentrated under reduced pressure. The residue was purified by silica gel column chromatography (10% EtOAc in cyclohexane) to afford 7-((1-(2-allylphenyl)allyl)oxy)-2H-chromen-2-one (**1e**) as a white solid (306 mg, 96%). ^1H NMR (500 MHz, $CDCl_3$) δ 7.60 (d, *J* = 9.5 Hz, 1H), 7.44 (d, *J* = 7.8 Hz, 1H), 7.32 (d, *J* = 8.6 Hz, 1H), 7.30 – 7.21 (m, 3H), 6.87 (dd, *J* = 8.6, 2.4 Hz, 1H), 6.79 (d, *J* = 2.3 Hz, 1H), 6.23 (d, *J* = 9.5 Hz, 1H), 6.11 (ddd, *J* = 16.4, 10.5, 5.5 Hz, 1H), 5.99 (ddt, *J* = 16.6, 10.1, 6.3 Hz, 1H), 5.93 (d, *J* = 5.5 Hz, 1H), 5.38 – 5.26 (m, 2H), 5.21 – 5.13 (m, 1H), 5.06 (dd, *J* = 17.1, 1.6 Hz, 1H), 3.69 – 3.37 (m, 2H).

The products for **1a**, **1c**, **1d**, **1f,** and **1g** were synthesized following a reported procedure[9].

The Hoveyda-Grubbs II catalyst (1 mol %) was added at room temperature. to a solution of diene substrates **1a-1f**, or enyne **1g** (1.0 equiv.) in CH_2Cl_2 (3 mL). The reaction mixture was stirred for 1 h at 40 °C and then concentrated under reduced pressure. The residue was purified by flash column chromatography to afford the RCM product.

Metathesis product of **1a**: 99% yield. ^1H NMR (500 MHz, $CDCl_3$) δ 7.72 (d, *J* = 8.3 Hz, 2H), 7.32 (d, *J* = 8.3 Hz, 2H), 5.65 (s, 2H), 4.12 (s, 4H), 2.43 (s, 3H).

Metathesis product of **1c**: 98% yield.^1H NMR (500 MHz, $CDCl_3$) δ 7.74 (d, *J* = 8.3 Hz, 2H), 7.32 (d, *J* = 8.3 Hz, 2H), 5.82 (ddd, *J* = 17.1, 10.1, 7.0 Hz, 1H), 5.72 – 5.68 (m, 1H), 5.59 – 5.50 (m, 1H), 5.31 (dt, *J* = 17.1, 1.2 Hz, 1H), 5.16 (dt, *J* = 10.1, 1.1 Hz, 1H), 4.19 (q, *J* = 2.3 Hz, 2H), 2.44 (s, 3H).

Metathesis product of **1d**: Quant. ^1H NMR (500 MHz, CDCl3) δ 7.31 (d, *J* = 8.5 Hz, 2H), 7.09 (dd, *J* = 8.5, 0.8 Hz, 2H), 6.07 (s, 1H), 5.97 (s, 2H), 4.27 (s, 4H), 2.30 (s, 3H).

Metathesis product of **1f**: Quant. ^1H NMR (500 MHz, $CDCl_3$) δ 5.61 (s, 2H) 4.20 (q, *J* = 7.1 Hz, 4H), 3.01(s, 4H), 1.25 (t, *J* = 7.1 Hz, 6H).

Metathesis product of **1g**: Quant. ^1H NMR (500 MHz, $CDCl_3$) δ 7.42 – 7.25 (m, 10H), 6.31 – 6.03 (m, 2H), 5.41 – 5.27 (m, 1H), 5.11 (dq, *J* = 11.1, 1.0 Hz, 1H), 4.80 (dq, *J* = 2.0, 1.0 Hz, 2H).

Computational design of Ru1·dnTRP

Binding site motif generation and docking

Three-dimensional structures of **Ru1**, used for binding site design, were computed by DFT. All calculations were performed using Gaussian 16 software[10]. Structural optimization and frequency calculations were performed with the B3LYP-D3 method along with 6-31G(d) basis set and the SDD ECP on the Ru atom. D3 dispersion correction was applied using the Becke-Johnson damping function[11]. The solvent effect of water was included using the CPCM solvation model during geometry optimization. Vibration frequency calculations were performed to confirm that the computed structure indeed corresponds to a minimum.

We considered 8 conformers of the 7-membered sulfonamide ring, based on the orientation of the SO_2 moiety and the NH groups (Supplementary Scheme 1). Additional conformational freedom was considered by sampling the rotation of the *i*Pr-O bond in three steps.

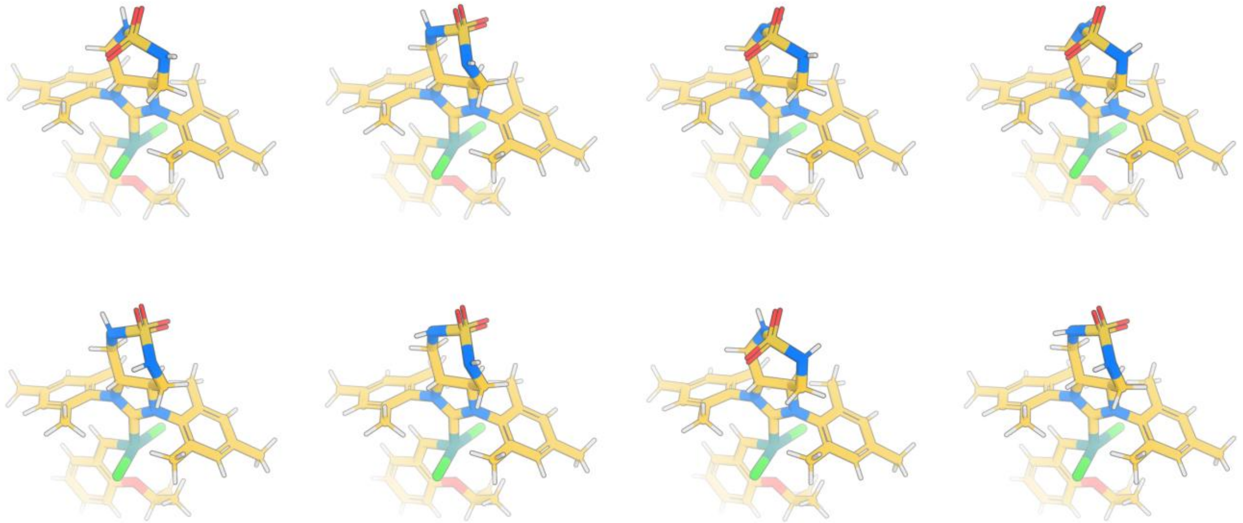

Supplementary Scheme 1. Conformers of **Ru1** reflecting the flexibility of the sulfamide ring.

A Rosetta ligand parameters file was created from the computed conformers by first converting the Gaussian outputs to mol2 files using Open Babel[12], and thereafter converting the mol2 file to Rosetta ligand parameters file using the `$ROSETTA_MAIN/source/scripts/python/public/molfile_to_params.py` script provided in Rosetta. Separate parameter files were further prepared for each sulfamide conformer to use them as unique inputs in the RifGen and RifDock steps.

We used RifGen[13] to identify beneficial interaction geometries between the ligand and amino acid side chains. In its essence, RifGen samples the placement of amino acid side chains against different parts of the molecule, and stores information about beneficial and valid inverse rotamers (backbone positions if side chain is fixed). We used RifGen to produce individual sets of inverse rotamers for each of the conformers of **Ru1**.

Supplementary Information

RifGen command: `14/rifdock/latest/rifgen @rifgen.flag`

The RifGen control file `rifgen.flag` is available for download on Github[15].

Next, we used RifDock[13] to identify combinations of inverse rotamers that would yield the best interaction energies and could be placed onto the provided protein backbones. We used parametrically-generated toroidal repeat protein (TRP) backbones as input scaffolds for RifDock, and to increase the diversity, relaxed the crystal structure of published TRP (PDB id: 4YXX)[16] using Rosetta FastRelax in multiple trajectories.

For each input scaffold, allowed pocket positions of TRP were enumerated (in file `positions.pos`): `4, 7, 8, 11, 12, 15, 39, 42, 43, 46, 47, 50, 74, 77, 78, 81, 82, 85, 109, 112, 113, 116, 117, 120, 144, 147, 148, 151, 152, 155, 179, 182, 183, 186, 187, 190.`

The fifty best-scoring docks were saved for each scaffold and **Ru1** conformer combination.

RifDock command:

```
{RIF_PATH}/rifdock/latest/rif_dock_test @/ rifdock.flag -rif_dock:scaffolds scaffold.pdb -rif_dock:scaffold_res positions.pos -rif_dock:outdir {outdir}
```

The RifDock control file rifdock.flag is available for download on Github[15].

Ligand binding site design

After having placed the cofactor **Ru1** and its interacting sidechains into the TRP scaffold with RifDock, the rest of the protein sequence was optimized to create additional interactions with the ligand and to further stabilize the interacting residues, Supplementary Fig. 1. Rosetta FastDesign[17] was used to perform the sequence design, and it was applied in two iterations. In iteration, design was performed while applying distance constraints between any hydrogen bond donors and acceptors of the ligand and sidechains that got introduced by RifDock. In the second stage, these constraints were relaxed and the sequence was further refined with FastDesign. The designed structures were thereafter scored for metrics describing the protein-ligand interactions (interface energy, ligand SASA, shape complementarity, number of polar contacts, substrate SASA, orientation of the cofactor **Ru1**) and pocket features (no-ligand-repack RMSD of interacting residues).

```
python/scripts/post_rifdock_FastDesign.py --pdb input.pdb --nstruct 5 --repack --min_polar 2 --outdir {outdir} --params {PATH}/ligand/HGS.params --ramp_cst_weights 10.0 0.0 --no_ala_design
```

The designs were selected based on the following metrics:

Metric	Threshold
Ligand – H-bond partner constraint score	<= 8.0
Mean no-ligand-repack RMSD of RIF residues	<= 0.6
Max no-ligand-repack RMSD of RIF residues	<= 1.0
Shape complementarity	>= 0.6
Ligand relative SASA (SASA_bound / SASA_free)	<= 0.2
Number of polar contacts to the ligand	>= 3.0

Number of H-bond acceptors near ligand NH	>= 1.0
Rosetta total score per residue	<= -3.0
Ligand interface energy	< 50% (below median)
Substrate SASA (of the *i*PrO-Ph moiety)	>= 30.0
Angle between ligand long axis and the toroid pore (ligand orientation)	>= 30.0 & <= 40.0

The designs passing the above filters were manually inspected and 23 were selected for experimental evaluation.

Expression of dnTRP in shake flasks and 96-well microtiter plates

The pET-29b plasmids carrying the DNA of dnTRPs were transformed into *E. coli* LEMO21 (DE3). The transformed cells were spread on LB agar plate (with 50 µg/mL kanamycin) and incubated (37 °C, 14h), individually.

For expression in flasks, colonies of each transformation were picked and inoculated into a main culture (ZYP auto-induction medium (200 mL), kanamycin (400 µg/mL), 1L baffled shake flask) and further incubated (25 °C, 180 rpm) until $OD_{600} \geq 12$. After expression, the cells were harvested by centrifugation (4 °C, 5000 g, 15 min) and then stored at -20 °C.

To purify the dnTRP, the cell pellets were resuspended and lysed (37°C, 300 rpm for 1 h) with Tris/HCl buffer (50 mL, 25 mM, NaCl (300 mM), lysozyme (1 mg/mL), DNase1 (0.025 mg/mL), pH 7.8). Samples were sonicated on ice (60 % amplitude, 2 seconds on/off, 3 min). The cell lysate was then centrifuged (4 °C, 20,000 g, for 30 min) to separate the clear supernatant from the pellet. The resulting clear supernatant was mixed with imidazole (final concentration = 25 mM) and loaded onto a precast Ni-IDA column (Protino Ni-IDA 2000 kit, MACHEREY-NAGEL). Purified dnTRP was eluted with Tris/HCl buffer (25 mM, NaCl (300 mM), imidazole (250 mM), pH 7.8). Analysis of the clear supernatant of cell lysate, the pellet cell lysate, and the purified protein was performed by sodium dodecyl sulfate–polyacrylamide gel electrophoresis (SDS-PAGE), Supplementary Fig. 2. Purified proteins were dialyzed three times in Tris/HCl buffer (15 L, 25 mM, NaCl (300 mM), pH 7.8) to remove the imidazole. The dialyzed samples were collected and concentrated to 3-6 mg/mL (Amicon® Ultra Centrifugal Filter, 10 kDa MWCO).

For expression in microtiter plates, a single colony was inoculated into a culture (ZYP auto-induction medium (1 mL), kanamycin (400 µg/mL), round-bottom 2 mL 96-well plate) and further incubated (25 °C, 300 rpm, 36 h). After expression, cells were harvested by centrifugation (4 °C, 4400 g, 30 min) and stored (at -20 °C) until use.

To prepare the dnTRP cell-free lysate for library screening in 96-well microtiter plates, cell pellets were lysed (37 °C, 300 rpm, 2 h) in NaOAc buffer (250 µL, 100 mM, $MgCl_2$ (500 mM), $[Cu(Gly)_2]$ = 5 mM, lysozyme (1 mg/mL), *DNase*1 (0.025 mg/mL), pH 4.2). The lysed samples were frozen (-20 °C, 16 h), thawed, and further incubated (37 °C, 300 rpm, 1 h). The cell-free lysate (clear supernatant of the cell lysate, hereafter CFE) was obtained by centrifugation (4 °C, 4400 g, 1 h). For the screening assay, the CFE (95 µL) was carefully transferred into a new assay 96-well plate using the Liquidator™ 96-channel benchtop pipettor (volume range 5-200 µL). Control samples

using *E. coli* LEMO21(DE3) transformed with an empty pET-29b vector were prepared similarly for assay evaluation and optimization.

Circular dichroism (CD) spectroscopy of dnTRP

CD spectra of dnTRPs were recorded by CHIRASCAN V100 (Applied Photophysics, United Kingdom). For CD spectra at different pHs, dnTRP_18 was first buffer-exchanged by ultrafiltration into citric-phosphate buffer (50 mM, NaCl (500 mM), pH 2.6), NaOAc buffer (100 mM, $MgCl_2$ (500 mM), pH 3.5), NaOAc buffer (100 mM, $MgCl_2$ (500 mM), pH 4.2), or Tris/HCl buffer (50 mM, NaCl (500 mM), pH 8.0). The dnTRP_18 (40 µM) in the corresponding buffer was then diluted 20-fold with Milli-Q water to a final concentration of 2 µM. The diluted sample (400 µL) was transferred in a quartz cuvette (light path 2 mm) and the spectrum was recorded from 260 to 190 nm. For CD spectra to determine thermal stability, dnTRP_18 (2 µM) in Tris/HCl buffer (1 mL, 2.5 mM, NaCl (25 mM), pH 8.0) was incubated (98 °C, from 6 min to 120 min) and immediately chilled on dry ice. The sample (400 µL) was then transferred into a quartz cuvette (light path 2 mm) and the CD spectrum was recorded from 260 to 190 nm. The CD spectra are displayed in Supplementary Fig. 3.

Determination of the binding affinity (K_D) of Ru1 to dnTRPs

The binding affinity of **Ru1** to dnTRP was determined using a tryptophan fluorescence-quenching assay. In brief, samples of dnTRP (198 µL, 1.01 µM in corresponding buffer) were pipetted into a 96-well plate (flat bottom, black). The cofactor **Ru1** (2 µL, 0.5 µM ≤ [**Ru1**] ≤ 4 mM in DMSO) was added to the wells and the fluorescence was recorded (excitation: 280 nm, emission: 300 to 400 nm (scanning mode) or 335 nm (kinetics mode)) using a TECAN 1000pro plate reader. The K_D was calculated by fitting the data to Supplementary Equation (1).

$$\boldsymbol{Supplementary\ Equation\ (1)}: \quad [PL] = \frac{P_{tot} + L_{tot} + K_D - \sqrt{(P_{tot} + L_{tot} + K_D)^2 - 4P_{tot}L_{tot}}}{2}$$

Where P_{tot} is the total dnTRP concentration (1 µM), L_{tot} is the total **Ru1** concentration titrated. *[PL]* is the concentration of the **Ru1**·dnTRP complex which is correlated to the fluorescence signal.

Native mass spectrometry of Ru1·R0

Purified dnTRP_R0 (153 µM) and [**Ru1**] = 53 µM were co-incubated in MES buffer (20 mM, NaCl (150 mM), DMSO (5.2% (vol/vol)), pH 5.1). The buffer was exchanged with ammonium acetate (50 mM, pH 6.8) by repeated cycles of ultrafiltration to a dilution factor of 100,000 (Amicon, MWCO 10 kDa). The sample was then injected into a Bruker maXisII ESI-QTOF using a syringe pump at a flow rate of 5 µL/min. The sample was analyzed in ESI+ mode with 140 µs transfer time, 40 µs PrePulse Storage, 10 eV collision energy, and an isCID ranging from 0-150 eV, adjusted according to the thickness of the charge envelope observed. Data analysis was carried out using Compass Data Analysis software (Bruker). Spectra were baseline subtracted, smoothed and de-convoluted using a high-resolution maximum entropy set-up. All samples were internally calibrated using a low concentration tuning mix (Agilent) and a linear calibration mode.

Size-exclusion chromatography of Ru1·R0

Purified dnTRP_R0 (1 mg/mL, MES (50 mM), NaCl (150 mM), pH 5.2) was incubated with [**Ru1**] = 40 µM (4 °C, 1 h). The mixture was then injected into an analytical Superdex 200 10/300 GL size-exclusion column and eluted with a MES buffer (50 mM, NaCl (150 mM), pH 5.2). Fractions corresponding to monomeric **Ru1**·R0 were collected.

Optimization Ru1·dnTRPs-based RCM in cell-free extract (CFE)

To investigate the RCM activity in CFE, **Ru1** (3 µM, 2 µL from a freshly prepared stock (150 µM in DMSO)) was added to the CEF of empty vector (97 µL, spiked with or without dnTRP_18 (20 µM)) in a 96-well plate (MASTERBLOCK®, 96 WELL, PP, 0.5 ML, V-BOTTOM, GREINER BIO-ONE). The samples were incubated (30 °C, 300 rpm, 1 h), followed by addition of the substrate **1a** (1.25 µL, 200 mM stock in DMSO, final concentration is 2.5 mM) to initiate the RCM reaction. RCM reactions were performed by incubation (25 °C, 300 rpm, 18 h). To investigate the effect of DMSO, $Cu(Gly)_2$, or diamide on the RCM activity, the CFE from empty vector was prepared in three different buffer conditions: NaOAc buffer (100 mM, $MgCl_2$ (500 mM), lysozyme (1 mg/mL), *DNase*1 (0.025 mg/mL), DMSO (12.75 % (vol/vol), pH 4.2), containing DMSO (12.75% (vol/vol)), [$Cu(Gly)_2$] = 5 mM, or [diamide] = 5 mM, respectively. The results are summarized in Supplementary Fig. 6a.

To investigate the effect of dnTRP_18's concentration on the TON of RCM in CFE, **Ru1** (0.5 µM) was added to the CEF of empty vector (95 µL, spiked with varying 0 ≤ [dnTRP_18] ≤ 25 µM (5µL)) in the 96-well plate (MASTERBLOCK®, 96 WELL, PP, 0.5 ML, V-BOTTOM). The samples were incubated (30 °C, 300 rpm, 2 h), followed by addition of the substrate **1a** (1.25 µL, 200 mM stock in DMSO, final concentration is 2.5 mM) to initiate the RCM reaction. RCM reactions were performed by incubation (25 °C, 300 rpm, 18 h). The results are summarized in Supplementary Fig. 6b.

Characterization of evolved Ru1·dnTRP variants

Validation of evolved variants

The validation of high-performing dnTRP_18 variants was performed using purified protein samples. Briefly, **Ru1** (5 µL from a freshly prepared stock in DMSO, final concentration is 0.5 µM) was added to the protein sample (95 µL, NaOAc buffer (100 mM), $MgCl_2$ (500 mM), purified dnTRP_18 variant, (21.1 µM), pH 4.2) in a glass vial (2 mL, Clear Robo Vial, 9mm Thread, Item No. VT009-1232). After sealing, the vial was incubated (30 °C, 250 rpm, 2 h), then chilled (5 min on ice), opened, and supplemented with substrate **1a** (1 µL, 250 mM stock in DMSO, final concentration is 2.5 mM). The vial was re-sealed, incubated (37 °C, 300 rpm, 18 h), chilled (5 min on ice), opened, and mixed with methanol (900 µL, containing benzyltriethyl-ammonium bromide (200 µM) as internal standard). The vial was re-sealed and incubated (37 °C, 300 rpm, 30 min) to quench the RCM. The vial was then placed in a 24-well plate and centrifuged (4 °C, 4400 g, 30 min), and the clear supernatant (800 µL) was transferred to a new vial for UPLC-MS analysis.

RCM by Ru1·dnTRPs at different pHs and temperatures

RCM reactions at different pHs and temperatures were performed similarly to the method described above for the validation experiments with purified dnTRP samples. For RCM reactions at different pHs, NaOAc buffer (100 mM, $MgCl_2$ (500 mM), pH 3.6, 4.2 or 5.2) and MES buffer (100 mM, $MgCl_2$ (500 mM), pH 6.0) supplemented with dnTRP (5 µM) were used. For RCM reactions at different temperatures, the sealed vials were incubated at various temperatures (37, 50, 70, or 90 °C, 300 rpm, 18 h).

RCM by Ru1·dnTRPs in the presence of glutathione (GSH)

RCM reactions in the presence of GSH were performed similarly as described above for the validation experiments with purified dnTRP samples. Purified dnTRP (10 µM) in NaOAc buffer (100 mM, $MgCl_2$ (500 mM), pH 4.2) or MES buffer (100 mM, $MgCl_2$ (500 mM), pH 6.0) was mixed with the cofactor **Ru1** (1 µM, 5 µL from a freshly prepared stock (20 µM in ice chilled NaOAc buffer (100 mM, $MgCl_2$ (500 mM), pH 4.2). A gradient concentration of GSH (0.1 mM ≤ [GSH] ≤ 4 mM), or a fixed concentration of GSH (1.5 mM) was added to the sample. The rest of the steps were carried out as described above.

X-ray structural analysis of Ru1·dnTRPs

Preparation of dnTRP-Δhis variants

The purified dnTRPs (1 mg/L, 10 mL, Tris/HCl, 25 mM, NaCl (300 mM), pH 7.8) featuring an N-terminal hexa-histidine and a TEV protease cleave site were supplemented with TEV protease (0.02 mg/L). Digestion was achieved by incubation (4 °C, 48h, no shaking). The sample was then loaded onto a precast Ni-IDA column and the flow-through containing cleaved dnTRP sample was collected. The size and purity of cleaved dnTRP samples were analysis with SDS-PAGE, Supplementary Fig. 9a. The pure dnTRP cleaved protein was then concentrated and buffer-changed by ultrafiltration (Amicon® Ultra Centrifugal Filter, 10 kDa MWCO). In the case of dnTRP_R0 (*apo*/*holo*), the protein was further purified by size-exclusion chromatography.

X-ray structures of apo dnTRP_R0-Δhis, Ru1·R0-Δhis and Ru1·R5-Δhis

Freshly purified dnTRP_R0-ΔHis (6.4 mg/mL, MES (50 mM), NaCl (150 mM), pH 5.2) was pipetted into MRC 3 well plates (reservoir volume (45 µL), drop volume (0.4 µL), SWISSCI, Switzerland) in a 1:1 ratio of sample solution to mother liquor. Crystals were harvested after 8 days of growth in sodium malonate (0.2 M, 20% (wt/vol) PEG 3350 (E8 PEG-Ion HT), pH 7.0).

Freshly purified and dnTRP_R0-ΔHis (5 mg/mL, MES (50 mM), NaCl (150 mM), pH 5.2) was incubated with **Ru1** (1:1 molar ratio) overnight at 4 ˚C. The sample was centrifuged (4400 g, 4 ˚C, 10 min) and pipetted into MRC 3 well plates (reservoir volume (45 µL), drop volume (0.4 µL)) in a 1:1 ratio of sample solution to mother liquor. Crystallography data were collected using the same protocol as described for the *apo* dnTRP_R0-Δhis.

Freshly purified dnTRP_R5-ΔHis (13 mg/mL, Tris/HCl (25 mM), NaCl (300 mM), pH 8.0) was set up for 96-well crystallization screens by mixing precipitation buffer (0.15 µL) using a robot (Crystal

Gryphon, Art Robins Instruments, USA; MRC 3-well plates), equilibrated against precipitation buffer (32 µL), and stored at 20 °C. Apo crystals were grown in sodium formate (2.0 M) and sodium acetate (0.1 M), pH 5.0 (Proplex™, Molecular Dimensions), and soaked with **Ru1**. **Ru1** (0.4 µL, 5 mM in DMSO) was mixed with the precipitation buffer (4.6 µL), and this mixed solution (2.5 µL) was added to the drop containing crystals. After soaking (48 h, 20 °C), the crystals were cryo-protected with ethylene glycol (25 % (wt/vol)) and flash-frozen in liquid nitrogen prior to data collection.

The data collection was carried out at the Diamond Light Source beam line I03 at wavelengths of 0.9763-0.9999 Å. XDS[18] was used for crystal indexing, integration, and AIMLESS[19] for scaling, within the graphical interface CCP4i2[20] of the CCP4 suite. The structures were solved by molecular replacement using PHASER/MOLREP[21] and an AlphaFold[14] search model. Iterative cycles of REFMAC5[22] and manual refinement in COOT[23] were used to improve the models. Ligand restraints were generated using eLBOW[24] and modified using REEL[25]. Modeling of the cofactor **Ru1** was based on residual electron density in the F_o-F_c map and anomalous dispersion density of Ru atom. Solvent molecules were not modelled because of low resolution. All figures were generated with PyMOL (PyMOL Molecular Graphics System, Version 2.5.0, Schrödinger, LLC). The PDB codes for the finalized structures of *apo* dnTRP_R0-Δhis, **Ru1**·R0-Δhis and **Ru1**·R5-Δhis are 9GVF, 8S6P, and 9H3C, respectively. Data collection and refinement statistics are listed in Supplementary Table 5**.**

Cell viability: colony formation assay

The viability of *E. coli* LEMO21 cells that harbor the cytoplasmic dnTRP R5 before and after the RCM was determined*.* To determine the cell viability prior to ring-closing metathesis (RCM), after expression, the cell density (OD_{600}) was immediately determined by aliquoting the cell culture (50 µL) into ZYP-5052 medium (950 µL). The cell culture was aliquoted (100 µL) into a sterile 1.5 mL microcentrifuge tube and subjected to a serial of dilution in ZYP-5052 to a final $OD_{600} = 3 \times 10^{-5}$. The diluted cell culture was aliquoted (100 µL) and plated onto three LB agar plates (supplemented with 50 µg/mL kanamycin). The plates were incubated (37 °C, 24 h) and the number of colonies on each plate was counted. A similar protocol was used to process the cells following the whole cell RCM reaction (as described in the Methods in the main text). The cell viability rate (%) after RCM is defined as the ratio (percentage) of colony number after RCM divided by the number of colonies before RCM. The whole-cell harboring **Ru1**·dnTRP_R5 retained 50 % cell viability after RCM (Supplementary Fig. 15d).

Supplementary Figures

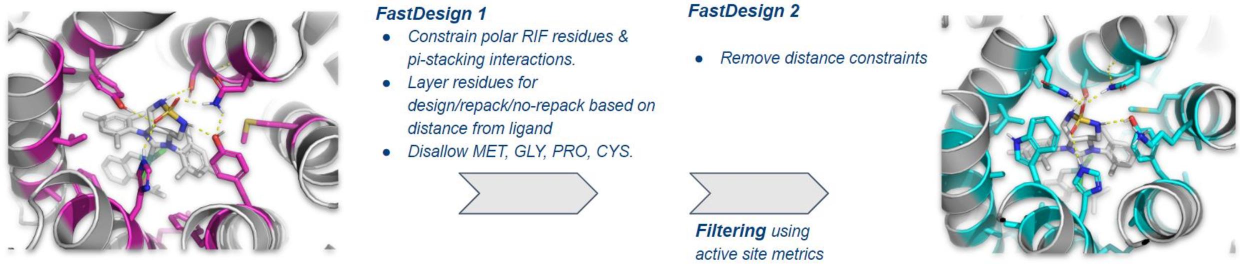

Supplementary Fig. 1. Procedure adopted for the design of the cofactor Ru1 binding site with Rosetta FastDesign.

In the first step, the sequence around the ligand was designed while the identities of RifDock-placed residues (shown in magenta) were fixed, and distances of polar interactions were constrained. In the second stage, the distance constraints were omitted and the sequence was re-optimized.

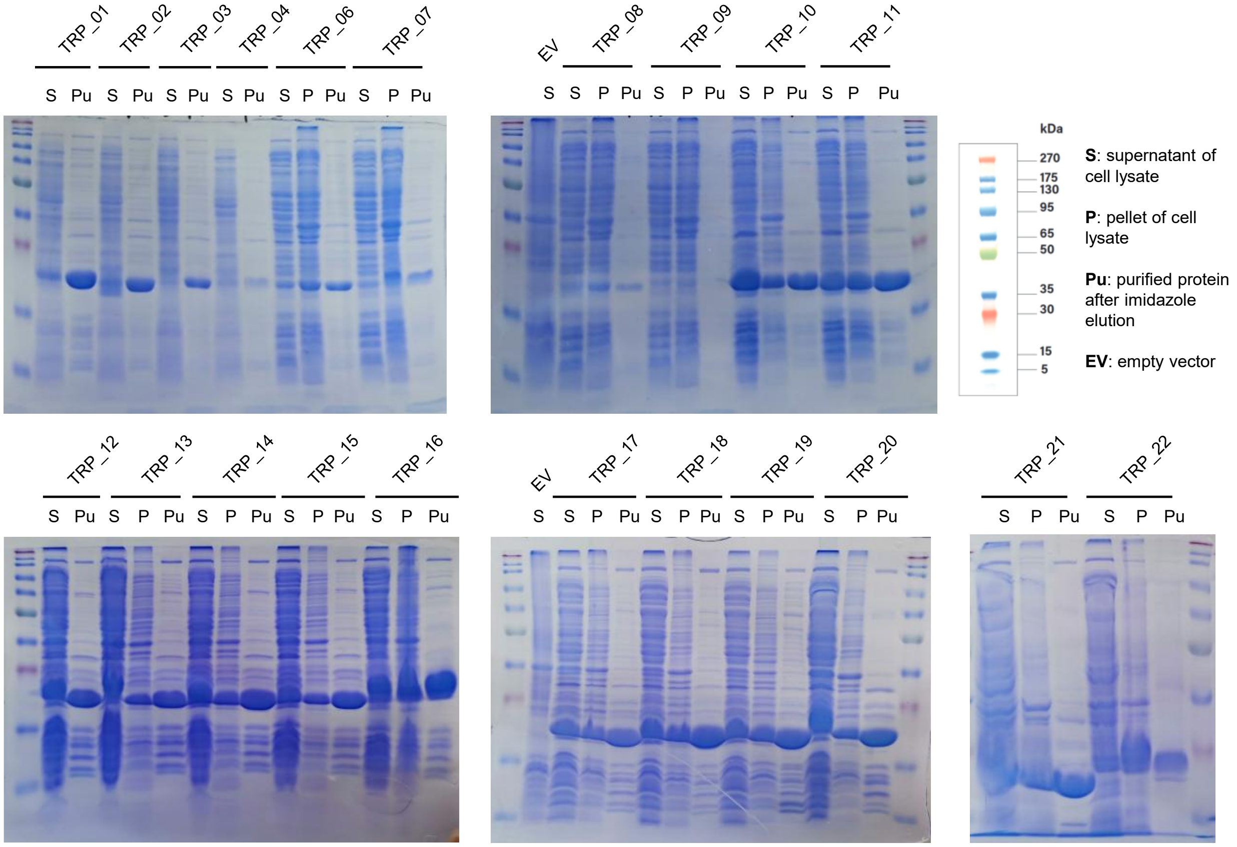

Supplementary Fig. 2. Expression and purification of dnTRP designs.

Colonies of transformed *E. coli* LEMO21 (DE3) were directly inoculated into ZYP auto-induction medium (supplemented with 400 µg/mL kanamycin) and incubated (25 °C, 180 rpm, 24 h). The harvested cell pellets were stored (-20 °C) before use. Cells were lysed (37 °C, 300 rpm for 1 h) with Tris/HCl buffer (25 mM, NaCl (300 mM), lysozyme (1 mg/mL), *DNase*1 (0.025 mg/mL)). The clear supernatant (**S**) and pellet (**P**) of the cell lysate were obtained by centrifugation (4 °C, 20,000 g, for 30 min). To purify the dnTRPs, the clear supernatant was mixed with imidazole (25 mM) and loaded into a precast Ni-IDA column. Purified dnTRPs (**Pu**) were obtained by eluting with Tris/HCl buffer (25 mM, NaCl (300 mM), imidazole (250 mM)). Analysis of the clear supernatant of cell lysate (**S**), the pellet cell lysate (**P**), and the purified elution fragment (**Pu**) was performed by SDS-PAGE.

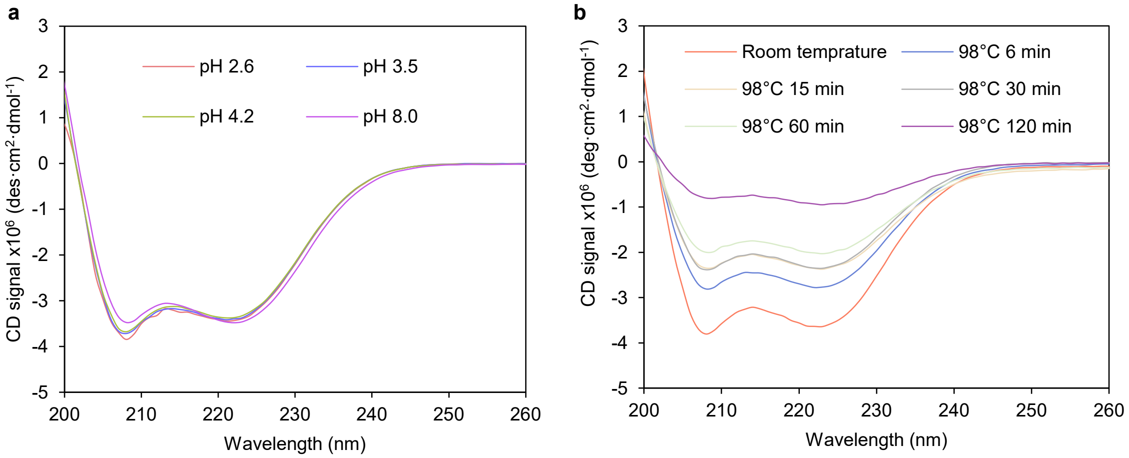

Supplementary Fig. 3. Circular dichroism (CD) spectra of dnTRP_18 at different pHs (a) and following incubation at 98 °C (b).

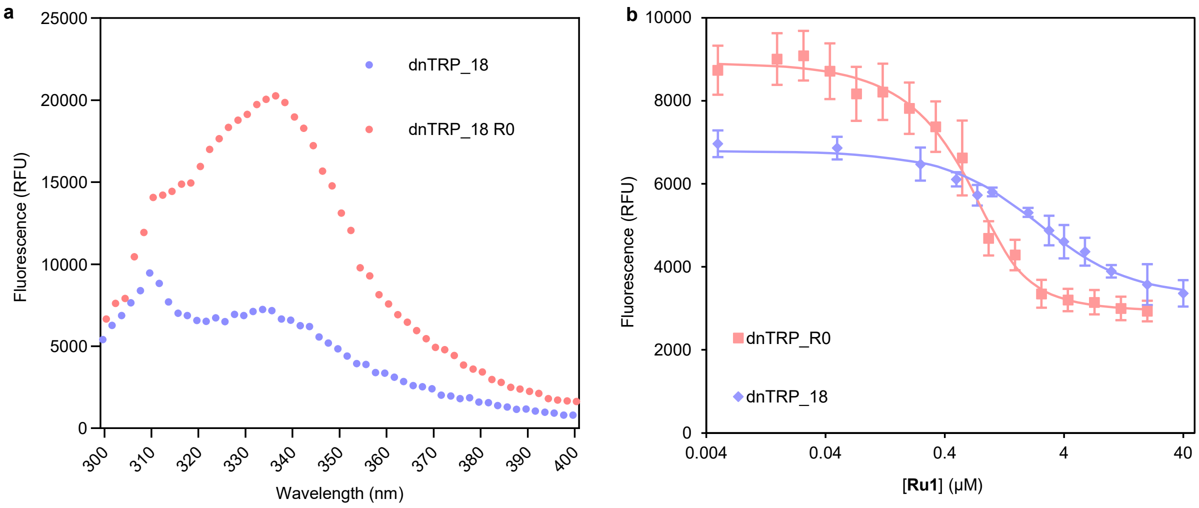

Supplementary Fig. 4. Tryptophan fluorescence-quenching assay for the determination of the binding affinity of Ru1·to dnTRPs.

a. Fluorescence scanning of dnTRP_18 and dnTRP_18_F116W (dnTRP_R0) in NaOAc buffer (100 mM, $MgCl_2$ (500 mM), pH 4.2). The sample was excited at 280 nm and emission was recorded between 300 - 400 nm. The emission at 335 nm was selected for further studies. **b.** Fitted curve of dnTRP_18 and dnTRP_R0 after incubation with cofactor **Ru1** in NaOAc buffer (100 mM, $MgCl_2$ (500 mM), pH 4.2) at room temperature for 2 h. The fitting procedure affords K_D = 1.95 ± 0.31 μM and 0.16 ± 0.04 μM for dnTRP_18 and dnTRP_R0, respectively. Data in **b** are displayed as mean values ± standard deviations of three replicates (n = 3). The replicates were independently performed using the same stock of each purified dnTRP.

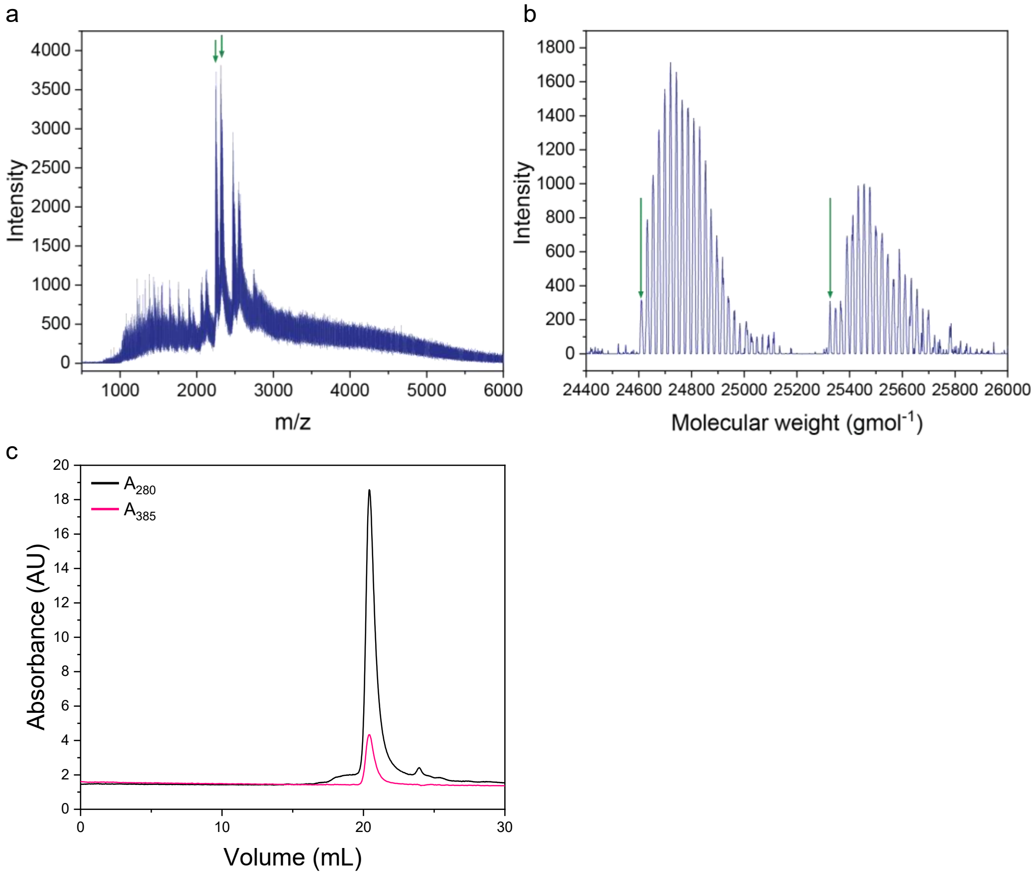

Supplementary Fig. 5. Native mass spectrometry and size-exclusion chromatography of Ru1·R0.

Untreated m/z spectra (shown in **a**) and the de-convoluted spectrum (shown in **b)** show one charge envelope from the apo dnTRP_R0 (monitored molecular weight: 24611.3 g·mol^{-1}, calculated molecular weight: 24610.08 g·mol^{-1}) and the holo **Ru1**·R0 (25325.8 g·mol^{-1}). The peaks of apo and holo spectra were highlighted by the green arrows. The average molecular weight difference between ten sets of apo/holo peaks is 712.63 ± 1.38 g·mol^{-1}, which corresponds with the calculated molecular weight of the cofactor (with the chlorides displaced by waters: 712.22 g·mol^{-1}). **c**. Size-exclusion chromatography of **Ru1**·R0. The protein and cofactor co-elute, with maximum absorption peaks at 280 nm and 385 nm, respectively.

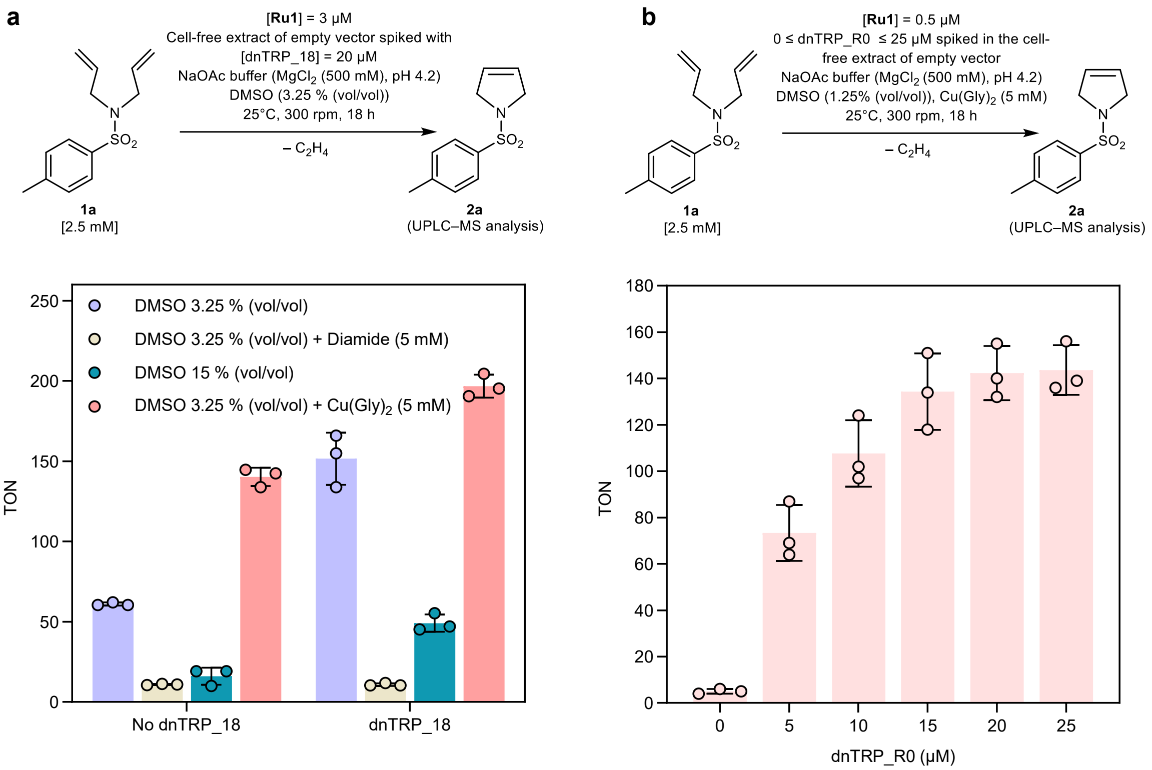

Supplementary Fig. 6. Optimization of ring-closing metathesis in cell-free extract (CFE).

a Evaluation of the effect of GSH-oxidizing agents on RCM activity: the CFE, spiked with cofactor [**Ru1**] = 3 µM was supplemented: DMSO (3.25 % or 15% (vol/vol)), or DMSO (3.25 % (vol/vol)) + Cu(Gly)$_2$ (5 mM), or DMSO (3.25 % (vol/vol)) + diamide (5 mM). **b** RCM of substrate **1a** in the presence of varying concentrations of dnTRP_R0. The CFE was prepared by lysis of empty vector cells with NaOAc buffer (100 mM, MgCl$_2$ (500 mM), lysozyme (1 mg/mL), *DNase*1 (0.03 mg/mL)). Data in panels **a** and **b** is displayed as mean values ± standard deviations of three replicates (n = 3). The replicates were independently performed using the same stocks of cell-free extract of empty vector, purified dnTRP_18, and purified dnTRP_R0.

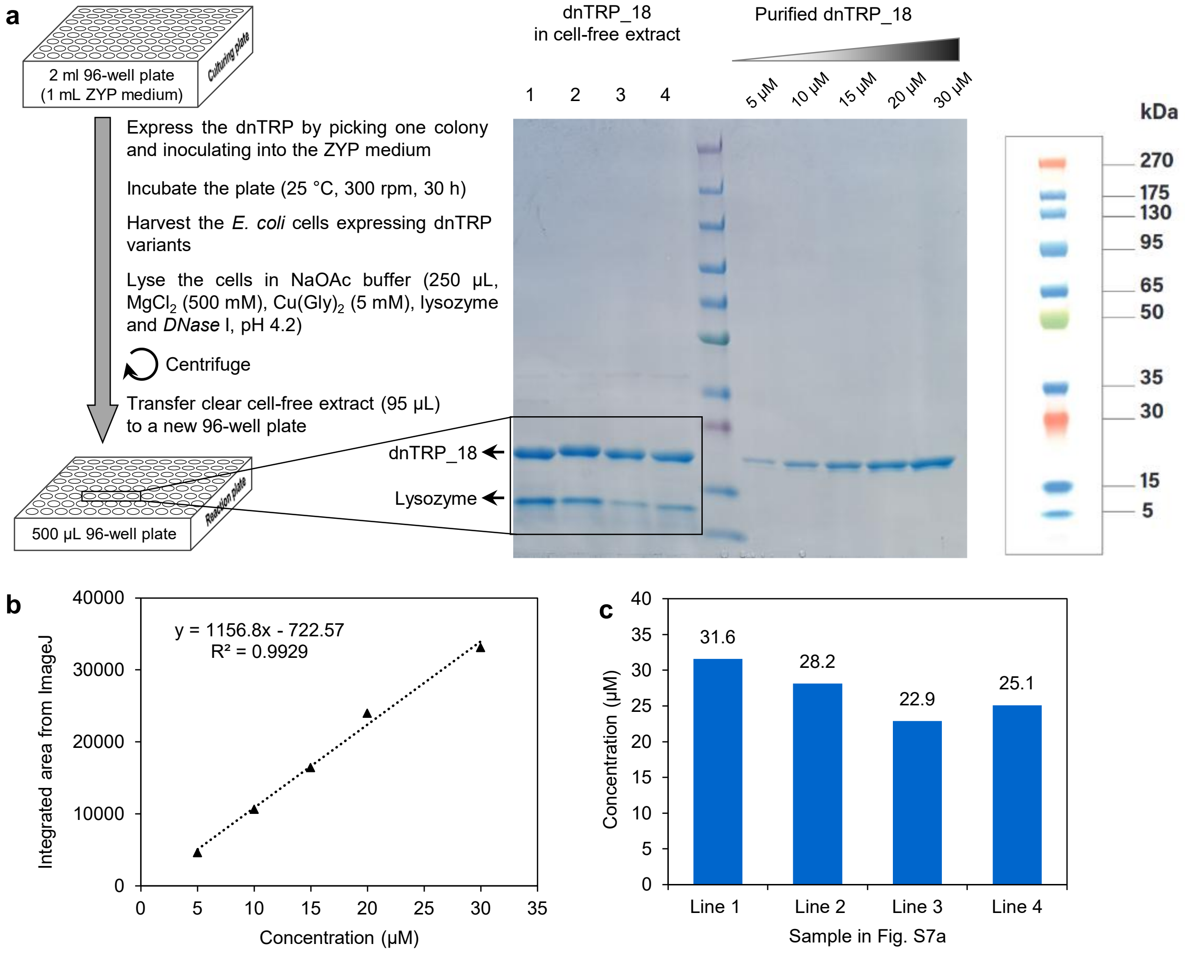

Supplementary Fig. 7. Expression and determination of the concentration of dnTRP_18 in the cell-free extract in a 96-well plate format.

a Left: Expression of dnTRP_18 in the 96-well plate (2 mL, round bottom). The clear supernatant of cell lysis (cell-free extract, CFE) was transferred into an assay plate and used for screening. For sodium dodecyl sulfate–polyacrylamide gel electrophoresis (SDS-PAGE), the cell-free extract was incubated (70 °C, 30 min) and centrifuged (4 °C, 21,000 g, 10 min). Middle: SDS-PAGE of the isolated CFE from four wells in the 96-well plate (Line 1 to 4, using the protocol from left) and different loading amounts of purified dnTRP_18. **b** Calibration curve generated from **a** by ImageJ 1.52a. **c.** Calculated concentration of dnTRP_18 in Line 1 to 4 in **a** using the calibration curve in **b**. The averaged concentration of the four wells was 26.9 ± 3.8 µM.

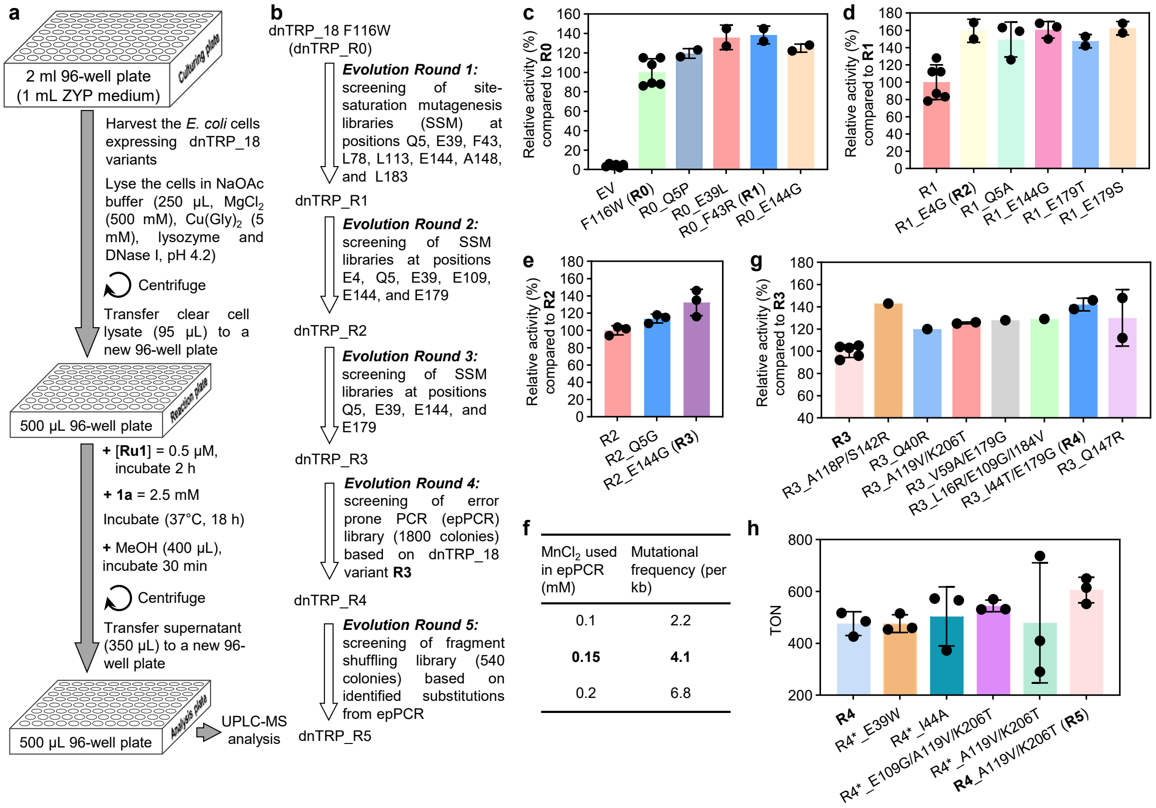

Supplementary Fig. 8. Directed evolution of Ru1·dnTRP using cell-free extracts at pH 4.2.

a Schematic representation of the high-throughput screening assay of **Ru1**·dnTRPs using cell-free extract (CFE) in a 96-well plate format. **b** Summary of the directed evolution campaign of **Ru1**·dnTRP using CFE. Rounds 1, 2 and 3 were performed by screening site-saturation mutagenesis libraries. Rounds 4 and 5 were performed by screening an error-prone PCR (epPCR) library and a gene shuffling library, respectively. The identified variants in round 1 (**c**), 2 (**d**) and 3 (**e**) SSM libraries screening. **f** Mutational frequency (mutations in per kilo base pair) of the transformed libraries using different concentrations of $MnCl_2$ (0.1, 0.15 and 0.2 mM) in the epPCR. **g** Relative activity of the variants compared to the parent (dnTRP_R3: dnTRP_18_E4G/F43R/F116W/E144G) identified from the epPCR library. **h.** Turnover number (TON) of variants (purified) identified from the fragment shuffling library in round 5. dnTRP_R4*: dnTRP_18_E4G/F43R/F116W/E144G/E179G. The identified variants in rounds 3 - 4 with the mutations lying in the corresponding range of the DNA sequence were used as the templates for each fragment PCR. The primers used for fragment and vector backbone PCRs are listed in Supplementary Table 4. In panels **c**, **d**, **e**, and **g**, the biological replicates are represented where two or more data points are shown within the bar chart. Data in panel **h** is displayed as mean values ± standard deviations from three replicates (n = 3). The replicates were independently performed using the same stock of purified dnTRP.

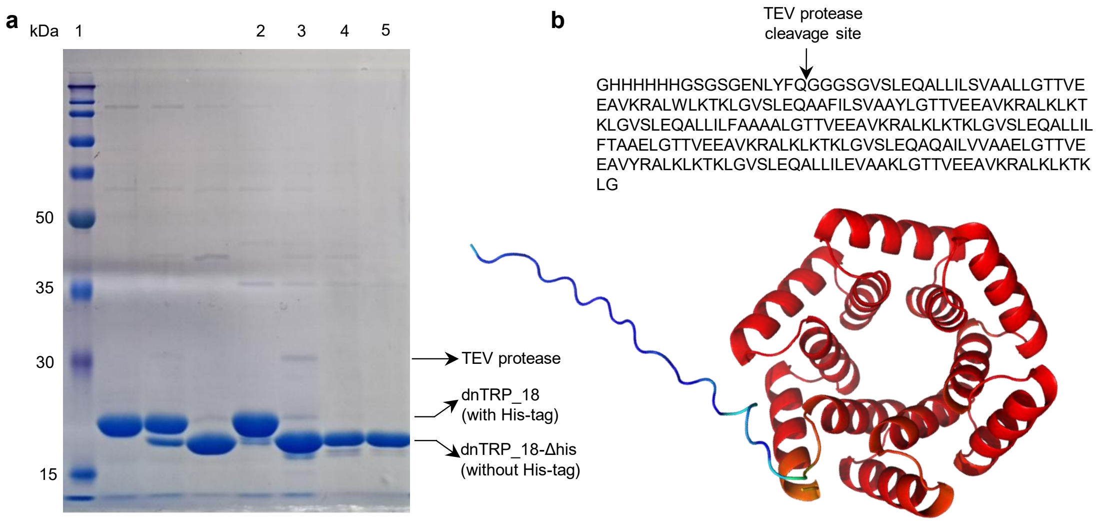

Supplementary Fig. 9. Removal of N-terminal His-tag from dnTRPs and determination of the corresponding K_D of various Ru1·dnTRPs.

a Sodium dodecyl sulfate–polyacrylamide gel electrophoresis (SDS-PAGE) of TEV-digested dnTRP_18 (as example). Line 1: protein standards; Line 2: dnTRP_18 (1 mg/mL) without TEV protease treatment; Line 3: TRP_18 (1 mg/mL) digested with (0.02 mg/mL) TEV protease (4 °C, 48 h); Line 4/5: Flow through and washed sample of line 3 after loading to Ni-IDA column; **b** AlphaFold2 predicted structure of dnTRP_R5 with N-terminal hexa-histidine and TEV protease cleavage sequences. The TEV protease cleavage site is highlighted with a red arrow.

a

pH	K_D for **Ru1**·dnTRP (μM)			
	dnTRP_R0-Δhis	dnTRP_R5	dnTRP_R5-Δhis	dnTRP_R5-Y50A/K190A-Δhis
4.2	0.35 ± 0.10	0.15 ± 0.03	0.24 ± 0.07	0.38 ± 0.07
5.2	1.01 ± 0.13	0.50 ± 0.13	0.33 ± 0.07	0.54 ± 0.10
6.0	0.55 ± 0.11	0.88 ± 0.15	0.20 ± 0.06	0.45 ± 0.05

d

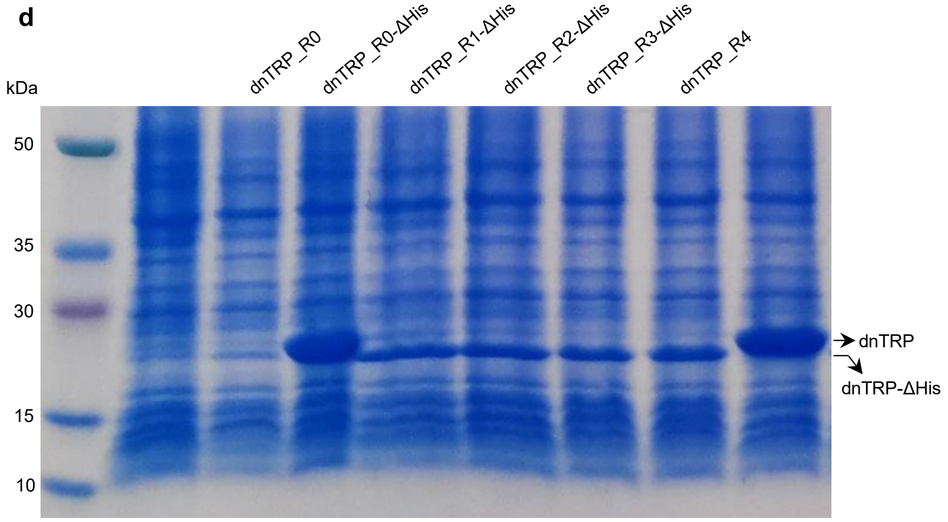

Supplementary Fig. 10. Binding affinity (to Ru1) and expression of dnTRP-Δhis in the cytoplasm of *E. coli*.

a. Binding affinity (K_D) of **Ru1** to purified dnTRP_R0-Δhis, dnTRP_R5, dnTRP_R5-Δhis and dnTRP_R5_Y50A/K190A-Δhis at different pHs. **b** Whole-cell SDS-PAGE of cytoplasmically expressed dnTRPs and their ΔHis variants. The data in **a** are displayed as mean values ± standard deviations of three replicates (n = 3). The replicates were independently performed using the same stock of each purified dnTRP.

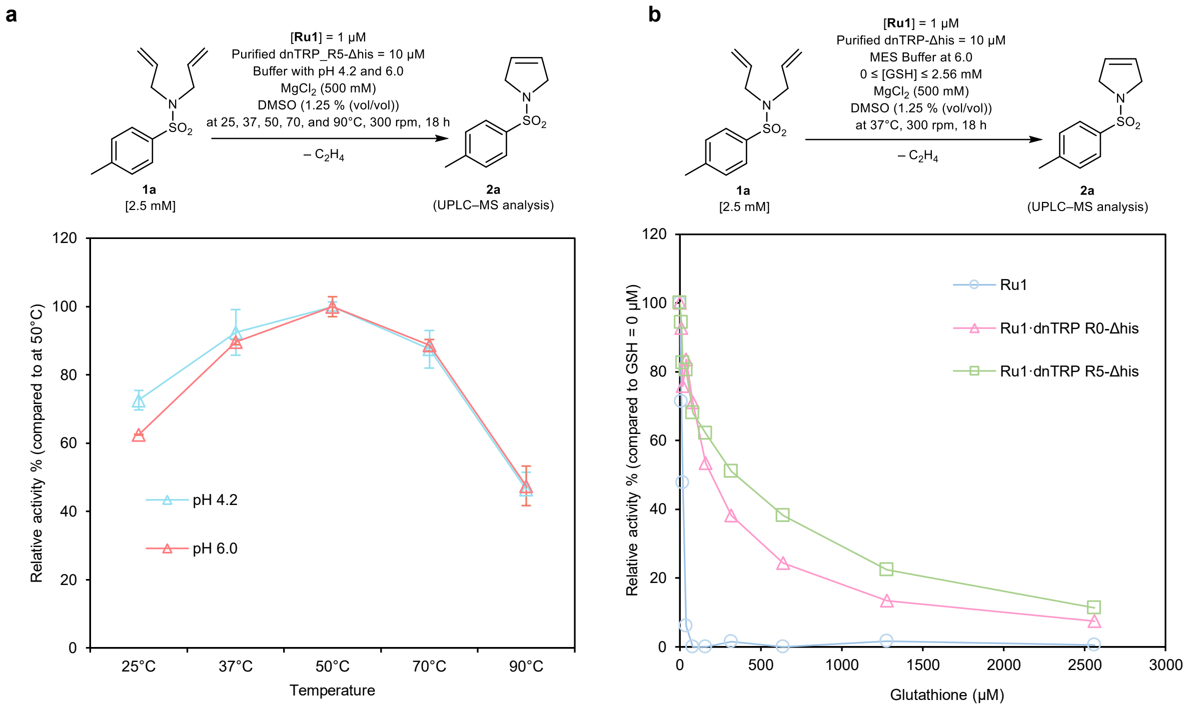

Supplementary Fig. 11. Activity of Ru1·dnTRPs at different temperatures and in presence of varying concentrations of glutathione (GSH)

a. Activity profiles of **Ru1**·R5-Δhis for ring-closing metathesis (RCM) with substrate **1a** at different temperatures. The RCM reactions were performed at pH 4.2 and 6.0. A 100% relative activity is defined as the activity at 50 °C. Data are displayed as mean values ± standard deviations of three replicates (n = 3). The replicates were independently performed using the same stock of the purified dnTRP_R5-ΔHis. **b** Activity profiles of **Ru1**, **Ru1**·R0-Δhis, and **Ru1**·R5-Δhis in the presence of various GSH concentrations. A 100% relative activity is defined as the activity in the absence of GSH. The RCM reactions were performed at pH=6.0. At pH=4.2, NaOAc buffer (100 mM, $MgCl_2$ (500 mM)) was used. At pH=6.0, MES buffer (100 mM, $MgCl_2$ (500 mM)) was used. Details regarding the reaction conditions, processing, and data acquisition are described in Supplementary Methods.

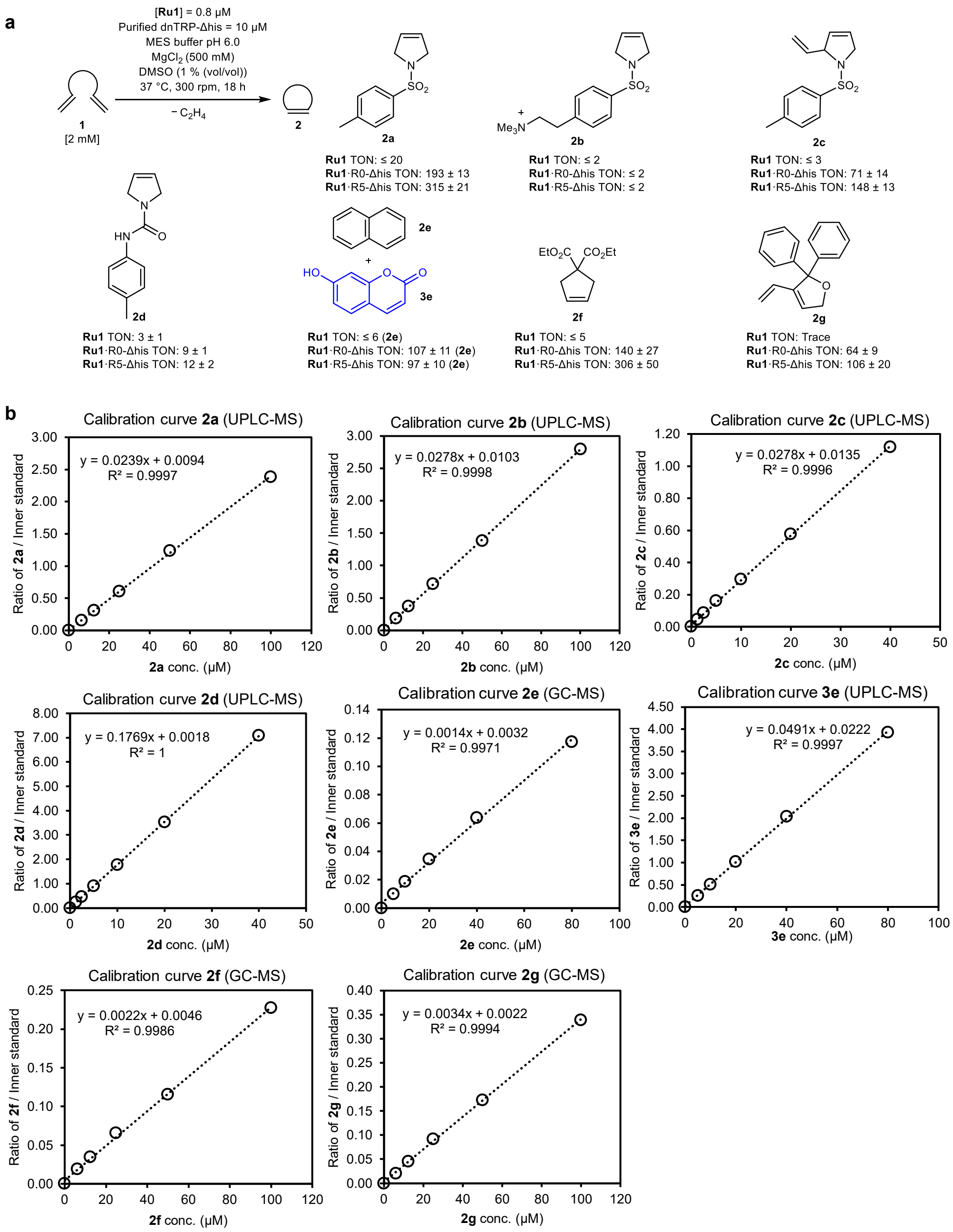

Supplementary Fig. 12. Comparison of RCM performance for different substrates catalyzed by Ru1 and Ru1·dnTRPs-ΔHis ArMs.

a RCM product structures **2a-g** and corresponding TONs for ring-closing metathesis catalyzed by **Ru1** and **Ru1**·dnTRPs. **b.** Calibration curves for cyclized products **2a**, **2b**, **2c**, **2d**, **2e**, **2f**, and **2g** for the TON quantification. Data in a is displayed as mean values ± standard deviations of three replicates (n = 3). The replicates were independently performed using the same stock of each purified dnTRP-ΔHis.

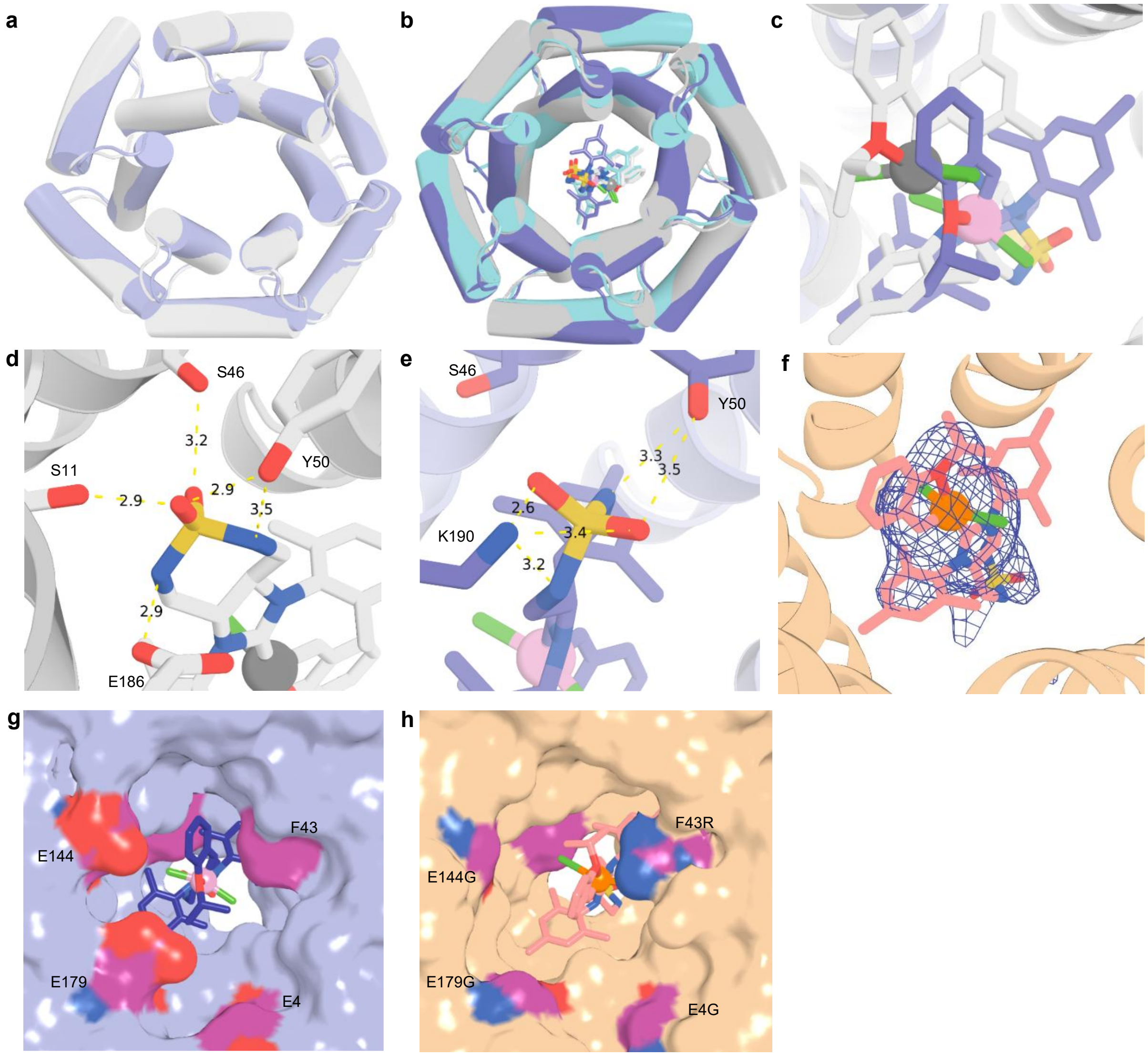

Supplementary Fig. 13. Structural characterization of Ru1·dnTRPs.

a Overlay of *apo* structures of dnTRP_R0 (design model: gray; X-ray structure: purple, PDB: 9GVF). The root mean square deviation (RMSD) of C_α atoms between these two structures was computed at 1.6 Å. **b** Back view (i.e. the side with the narrower cavity opening) of the overlay of the design model of **Ru1**·dnTRP_18 (gray, ruthenium: gray sphere), Rosetta-relaxed AF2 prediction of **Ru1**·R0-Δhis (cyan, ruthenium: blue sphere.), and the X-ray structure of **Ru1**·R0-Δhis (purple, ruthenium: pink sphere, PDB: 8S6P)**.** Helices in the protein scaffolds are displayed as cylinders. The C_α RMSD between these three structures ranges from 1.02 to 1.68 Å. **c** An expanded view highlights the positions of the **Ru1** cofactor within cavities of the **Ru1**·dnTRP_18 design model (gray) and **Ru1**·R0-Δhis X-ray (purple) structure. The ruthenium atoms in **Ru1**·dnTRP_18 (computed) and **Ru1**·R0-Δhis are presented as dark gray and pink spheres, respectively. A distance of 3.4 Å is determined between the computed and X-ray position of the

Ru ion. **d** Possible hydrogen bond contacts are predicted between the sulfamide moiety in **Ru1** and residues (S11, S46, Y50, and E186) in dnTRP_18 (computational model). **e** Tentative hydrogen bond contacts between the sulfamide moiety of **Ru1** and residues Y50 and K190 in **Ru1**·R0-Δhis (based on the 1.9 Å resolution X-ray structure). These hydrogen bonds are presented by yellow dashed lines. **f** Composite omit map (blue mesh) contoured at 1.0 σ of the cofactor region in the **Ru1** R5-dhis structure. **g, h** The surface view of inner cavities (the side with the wider cavity opening) in the X-ray structures of **Ru1**·R0-Δhis (purple) and **Ru1**·R5-Δhis (wheat). The amino acids (E4, F43, E144, and E179) mutated during directed evolution are highlighted in magenta.

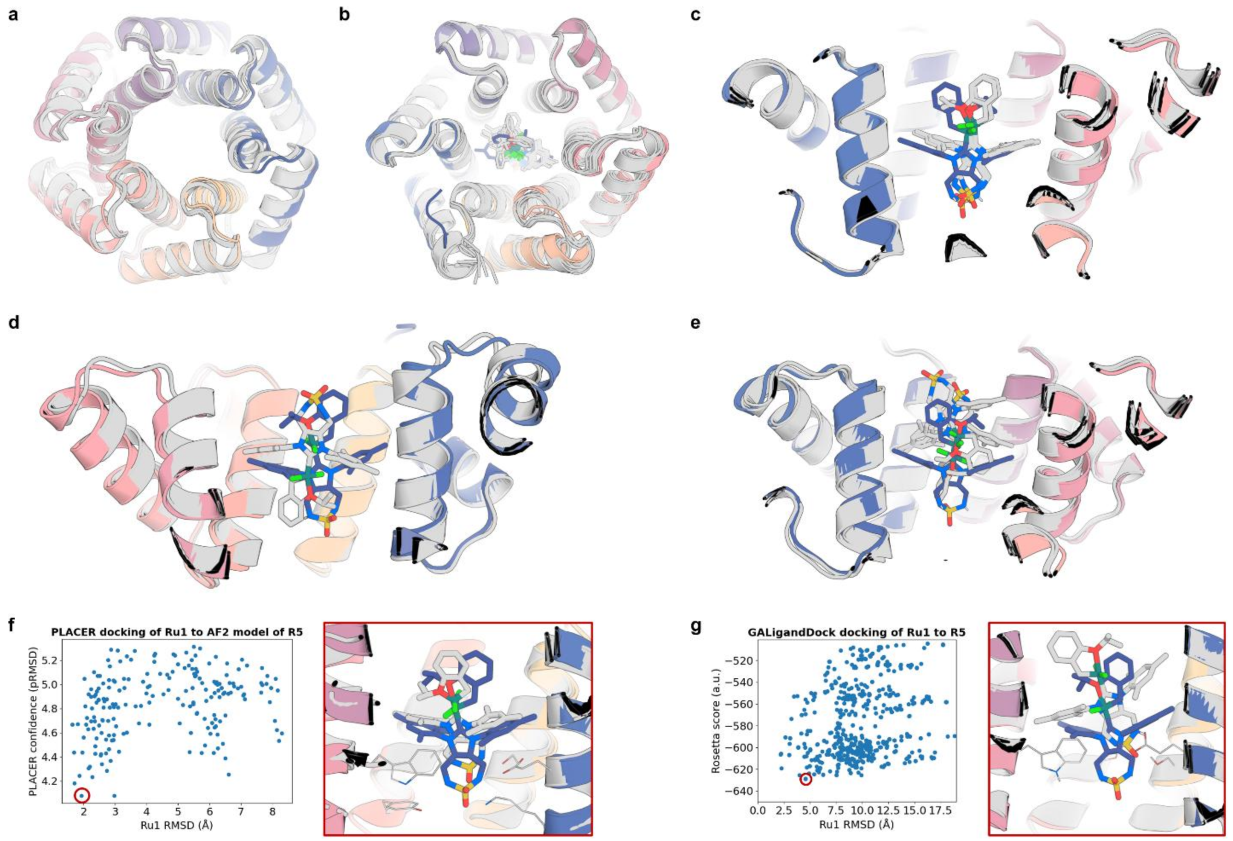

Supplementary Fig. 14. Structure prediction analysis of Ru1·dnTRPs.

a Overlay of the crystal structure of *apo* dnTRP_R0 (colored cartoon) with Chai-1[26] predicted structures (five models, gray cartoon). C_α RMSD values are within 1.0 and 1.1 Å. **b** Overlay of the crystal structure of *holo* **Ru1**·R5 with Chai-1-predicted structures (six models, gray cartoon and sticks). C_α RMSD values range from 1.5 to 1.8 Å. **c** Side-view of **Ru1**·R5 crystal structure (colored cartoon and sticks) and Chai-1 predictions (two models, gray cartoon and sticks), revealing the predicted orientation of the cofactor. Shown are models where Chai-1 correctly predicted the geometry around the ruthenium (Ru) atom in **Ru1**. **d** Side-view of **Ru1**·R5 crystal structure (colored cartoon and sticks) and AlphaFold3[27] predictions (gray cartoon and sticks), showing the cofactor having been predicted in upside-down orientation. **e** Side-view of **Ru1**·R5 crystal structure (colored cartoon and sticks) and Boltz-1[28] predictions (gray cartoon and sticks), showing the cofactor having been predicted in upside-down orientation. **f** Results of docking **Ru1** into AlphaFold2-predicted model of dnTRP_R5 with PLACER29. The plot correlates **Ru1** RMSD with the neural network confidence of the prediction (pRMSD score). Displayed in the red box is the highest confidence (lowest pRMSD) model (gray cartoon, sticks and lines) with **Ru1** RMSD of 1.92 Å against **Ru1**·R5 crystal structure (colored cartoon and sticks). **g** Results of docking **Ru1** into AlphaFold2-predicted model of dnTRP_R5 with Rosetta GALigandDock[30]. The plot correlates **Ru1** RMSD with the Rosetta energy of a given model. Displayed in the red box is the lowest energy model (gray cartoon, sticks and lines) with **Ru1** RMSD of 4.57 Å against **Ru1**·R5 crystal structure (colored cartoon and sticks).

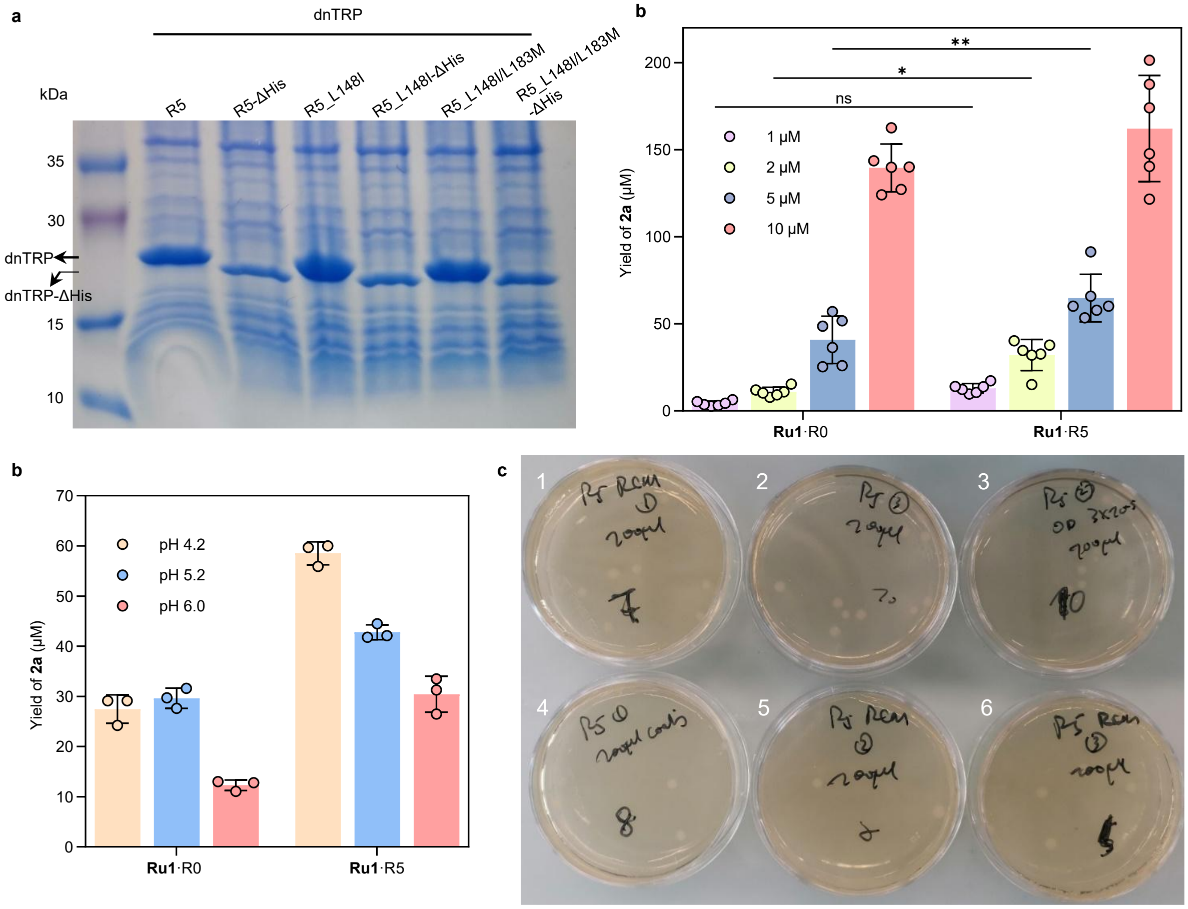

Supplementary Fig. 15. Expression of dnTRPs and optimization of [Ru1] concentration for the assembly of cytoplasmic Ru1·dnTRPs and corresponding cell viability determination.

a Whole-cell sodium dodecyl sulfate–polyacrylamide gel electrophoresis (SDS-PAGE) of cytoplasmically expressed dnTRP_R5, dnTRP_R5-ΔHis and their corresponding variants. **b** Ring-closing metathesis (RCM) of substrate **1a** using the *E. coli* whole cells harboring cytoplasmic **Ru1**·dnTRPs. Varying [**Ru1**] concentrations (1, 2, 5, and 10 μM) were incubated with *E. coli* cells to assemble **Ru1**·dnTRPs. The results represent the mean of six biological replicates with error bars indicating standard deviations (n = 6). **c** RCM of substrate **1a** using the *E. coli* whole cells harboring cytoplasmic **Ru1**·dnTRPs at different pHs. The results represent the mean of three replicates with error bars indicating standard deviations (n = 3). The replicates were independently performed using the same batch of cells that expressing dnTRP_R0 or dnTRP_R5 (incubated with [**Ru1**] = 2 μM). **d** Photo of colonies grown on agar plates used to evaluate cell viability of *E. coli* cell, following RCM. Plates 2, 3, and 4 were plated with *E. coli* cells which were harvested after expression. Plates 1, 5, and 6 were plated with *E. coli* cells after being used for whole-cell RCM. Two-way ANOVA for **b**. *$P < 0.05$, **$P < 0.01$; ns, not significant ($P > 0.05$).

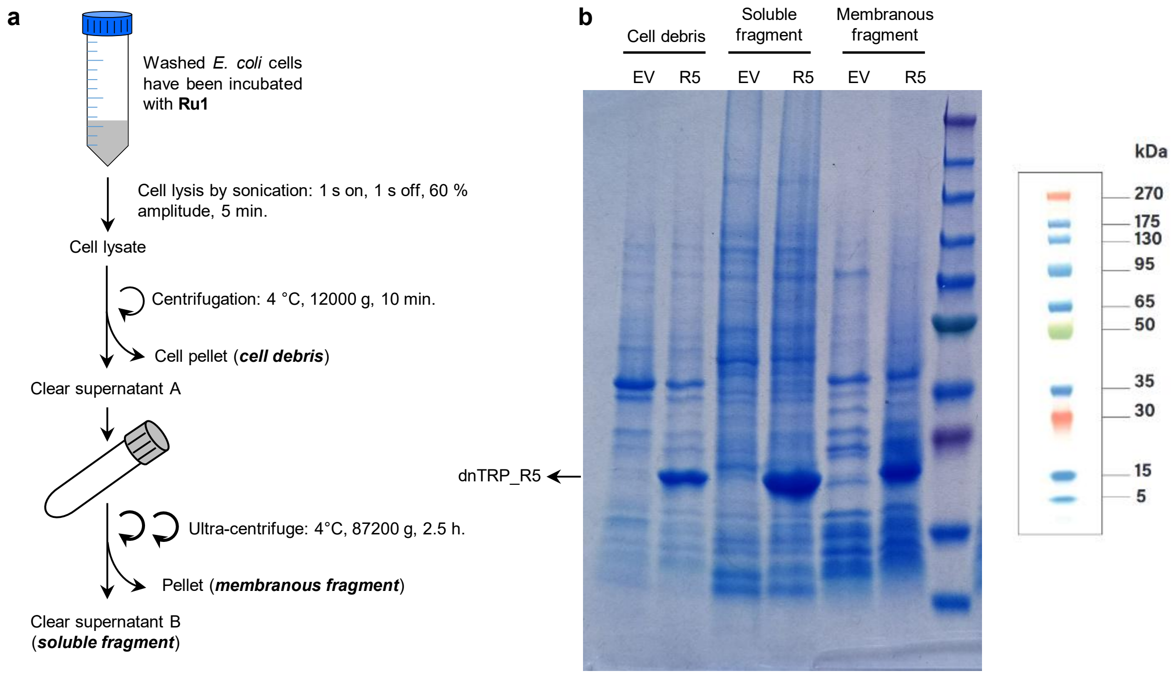

Supplementary Fig. 16. Fragmentation of *E. coli* cells for inductively coupled plasma mass spectrometry (ICP-MS) determination of [Ru] concentration.

a Schematic representation used for the preparation of cell fragments for ICP-MS analysis of [Ru]. **b** SDS-PAGE used to determine the content of dnTRP_R5 in the prepared cell fragments in **a**. EV: *E. coli* cells harbor the empty vector.

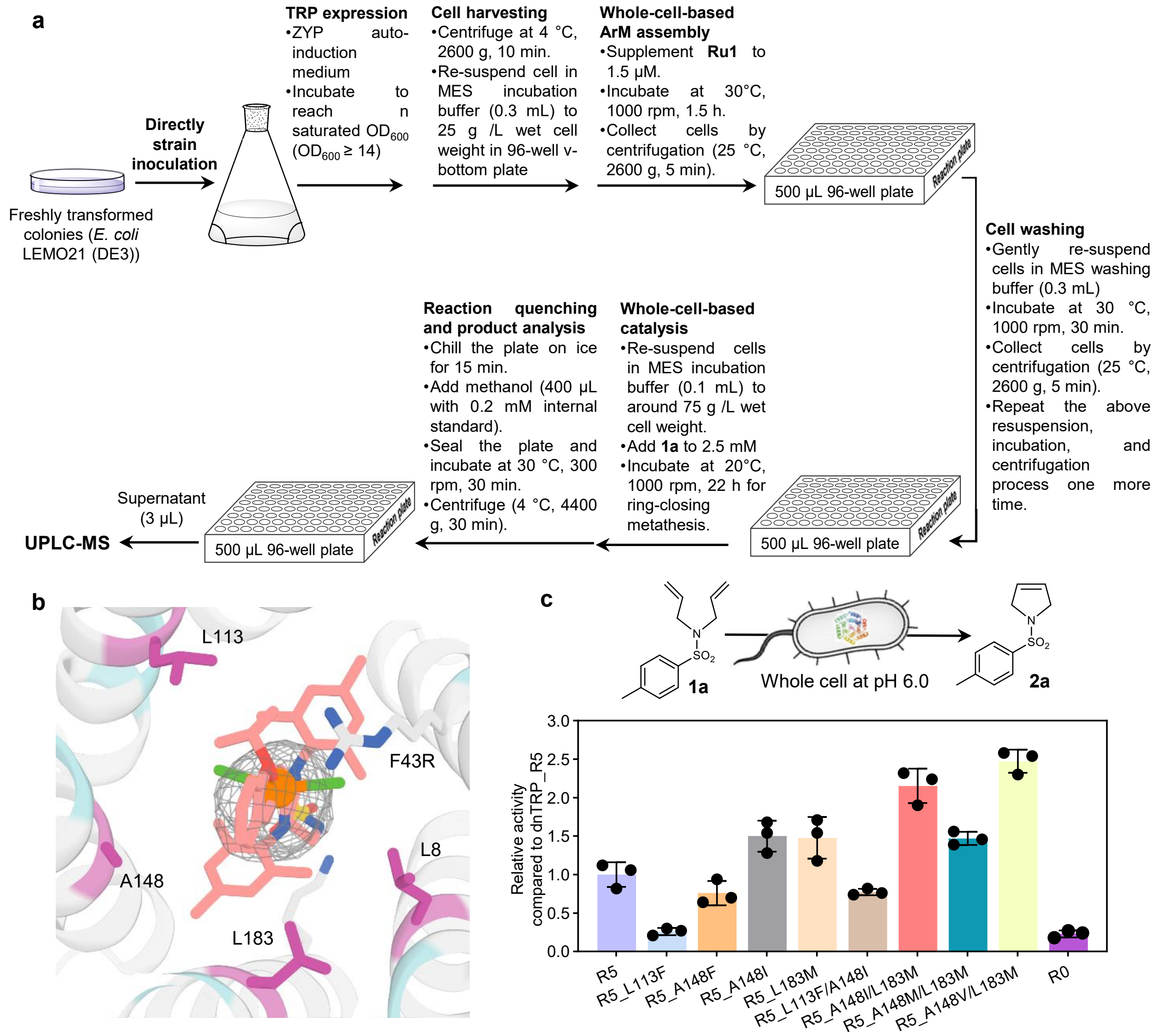

Supplementary Fig. 17. Screening dnTRP_R5 L8X, L113X, A148X and L183X variants at pH 6.0 for improved RCM activity in whole cells with the substrate 1a.

a Schematic representation of the assembly of the cytoplasmic artificial metathase and the subsequent activity screening in 96-well plate format. **b**. Close-up view of the X-ray of **Ru1**·R5-Δhis, highlighting (magenta) the four residues L8, L113, A148, and L183 selected for randomization using seventeen amino acids (except Cys and Pro). **c** Summary of the relative RCM activity of selected variants that exhibit superior cytoplasmic activity (in the whole-cell assay) compared to the parent dnTRP_R5. Relative activity is calculated as the yield for the cyclized product **2a** of the evolved variant divided by the yield obtained for the parent **Ru1**·R5. Data in panel **c** is displayed as mean values ± standard deviations from three biological replicates (n = 3).

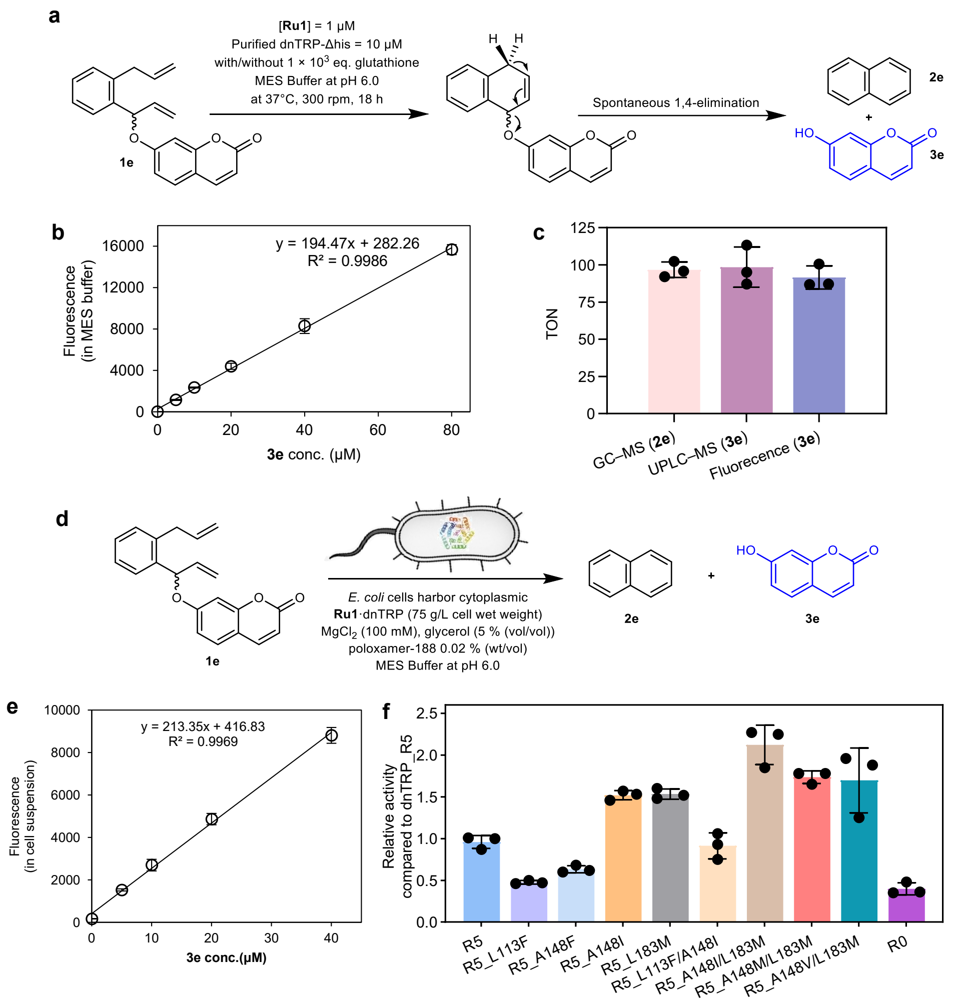

Supplementary Fig. 18. Ring-closing metathesis of diene 1e using Ru1·dnTRPs

a Reaction conditions for RCM using purified **Ru1**·dnTRPs using the profluorescent substrate **1e**. As a result of RCM followed by spontaneous 1,4-elimination, equimolar amounts of naphthalene **2e** and umbeliferone **3e** are produced and detected. **b** Calibration curve for **3e** using fluorescence detection using purified dnTRPs. **c** Comparison of the TONs for RCM of **1e** by quantifying naphthalene **2e** (via GC–MS) and umbelliferone **3e** (via UPLC–MS and fluorescence). **d** RCM of **1e** using *E. coli* cells harboring cytoplasmic **Ru1**·dnTRP. **e** Calibration curve for the fluorescence of umbelliferone **3e** in the presence of *E. coli* cells. **f** Summary of the relative RCM activity for selected metathase variants using substrate **1e**. Relative activity is calculated as the yield of the cyclized product **3e** divided by the yield obtained for the parent **Ru1**·R5. Data in panel **f** are displayed as mean values ± standard deviations from three biological replicates (n = 3).

a

Variant	pH	K_D of **Ru1**·dnTRP-Δhis (μM)
**Ru1**·R0-Δhis	3.6	0.64 ± 0.09
	6.0	0.55 ± 0.11
**Ru1**·R5-Δhis	3.6	1.15 ± 0.39
	6.0	0.20 ± 0.06
**Ru1**·R5_A148I/L183M-Δhis	3.6	0.72 ± 0.15
	6.0	0.18 ± 0.04
**Ru1**·R5_A148V/L183M-Δhis	3.6	0.95 ± 0.20
	6.0	0.17 ± 0.07

b

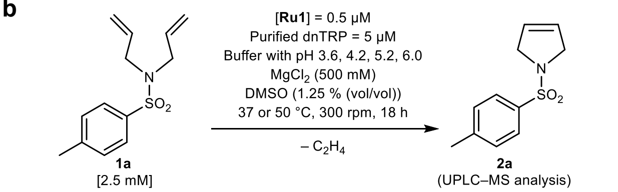

Variant (purified)	Temp (°C)	TON (pH 3.6)	TON (pH 6.0)
Ru1·R5-Δhis	37	810 ± 103	407 ± 87
	50	1028 ± 159	785 ± 138
**Ru1**·R5_A148I/L183M-Δhis	37	1160 ± 249	591 ± 94
	50	1122 ± 129	696 ± 45
**Ru1**·R5_A148V/L183M-Δhis	37	1198 ± 81	553 ± 58
	50	959 ± 86	559 ± 103

c

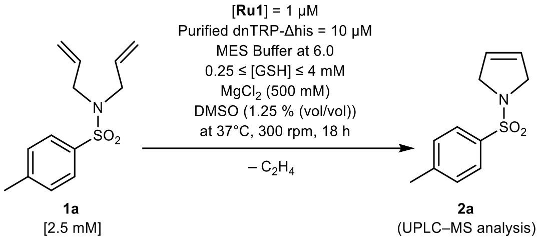

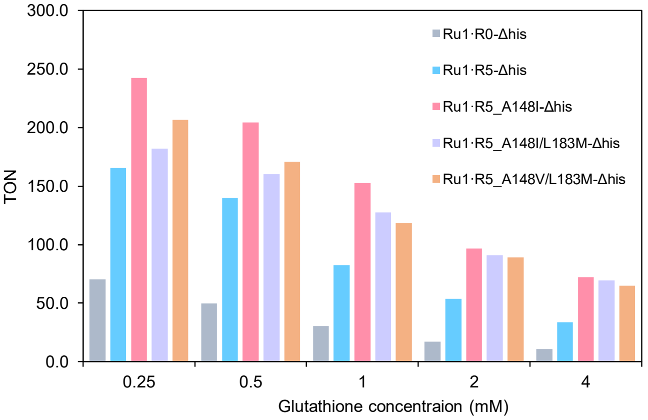

Supplementary Fig. 19. Binding affinity and turnover numbers of variants Ru1·R5_A148I/L183M-ΔHis and Ru1·R5_A148V/L183M-ΔHis.

a Binding Affinity (K_D) of **Ru1** to R5_A148I/L183M-ΔHis, and R5_A148V/L183M-ΔHis at pH 3.6 and 6.0. **b** Turnover number of **Ru1**·R5_A148I/L183M-Δhis and **Ru1**·R5_A148V/L183M-ΔHis at different pHs and temperatures (Temp). **c** Activity profile of variants using purified dnTRP-ΔHis proteins, spiked with increasing amounts of glutathione (GSH). The data in **a** and **b** are displayed as mean values ± standard deviations of three replicates (n = 3). The replicates were independently performed using the same stock of each purified dnTRP.

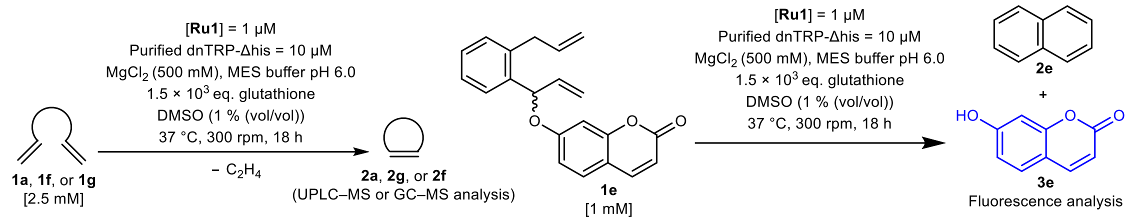

Variant	Product	TON (pH 6.0)	TON (with 1.5×10^3 eq. GSH, pH 6.0)
**Ru0**·R0-Δhis	**2a**	165 ± 25	24 ± 6
	**3e**	102 ± 13	6.0 ± 1.3
	**2f**	204 ± 40	Trace
	**2g**	66 ± 5	Trace
**Ru1**·R5-Δhis	**2a**	322 ± 15	78 ± 15
	**3e**	69 ± 11	13 ± 1
	**2f**	379 ± 12	49 ± 3
	**2g**	102 ± 20	39 ± 12
**Ru1**·R5_A148I/L183M-Δhis	**2a**	398 ± 29	116 ± 10
	**3e**	63 ± 21	21 ± 2
	**2f**	447 ± 80	96 ± 19
	**2g**	125 ± 33	69 ± 22
**Ru1**·R5_A148V/L183M-Δhis	**2a**	380 ± 53	117 ± 6
	**3e**	36 ± 5	18 ± 1
	**2f**	272 ± 22	79 ± 14
	**2g**	121 ± 44	77 ± 27

Supplementary Fig. 20. Turnover numbers of Ru1·dnTRP-Δhis (using purified proteins) in RCM of 1a, 1e, 1f, and 1g in absence or presence of thiols.

Supplementary Tables

Supplementary Table 1 Publications reporting *in (or on)-cellulo* new-to-nature reactions catalyzed by artificial metalloenzymes

Transformation	Scaffold_cofactor	Optimization strategy / throughput	catalytic performance achieved with evolved variants	Ref*
On cell surface: no glutathione (GSH) or thiols present				
Ring-closing metathesis (RCM)	hCA_sulfonamide-(NHC)Ru	Genetic /semi-rational design with low throughput (< 500 colonies)	Titre: 15 .5 mg·L^{-1} (equivalent to TON = 3.1 × 10^6 per cell)	47
Polymerization	NB4_maleimide-Cp*Rh	None	TON = 3.9 × 10^7 per cell	39
Deallylation	Sav_Biot-Cp*Ru	Genetic /semi-rational design with low throughput (< 500 colonies)	22-fold increase cell-specific activity vs. wild-type	38
Transfer Hydrogenation	hCA_sulfonamide-Cp*Ir	Genetic /semi-rational design with low throughput (< 1000 colonies)	2.8-fold increase cell-specific activity; up to 79 % ee	36
In *E.coli* periplasm: permeable outer-membrane, oxidizing environment, and minimal (reduced) GSH content				
Hydroamination	Sav_(Biot-NHC)Au	Genetic /semi-rational design by screening 400 mutants	> 7-fold increase vs. wild-type	20
Transfer hydrogenation	Chimeric Sav_Biot-Cp*Ir	Genetic / semi-rational design with medium throughput (> 1000 colonies)	TON = 137 (> 5-fold increase vs. wild-type)	35
RCM	Sav_(Biot-NHC)Ru	Genetic / directed evolution with medium throughput (> 3000 colonies)	TON = 5 × 10^5 per cell	37
RCM	Sav_(Biot-NHC)Ru	None	TON = 17	46
In cytoplasm: high GSH concentration				
Friedel–Crafts alkylation Diels–Alder reaction	LmrR_CuII-Phen	Genetic /semi-rational design with low throughput (< 300 colonies)	Friedel–Crafts alkylation: TON > 41; e.e. > 98% Diels–Alder reaction: TON > 5; e.e. > 84%	31
Cyclopropanation	CYP119_Ir(Me)MPIX	Genetic / semi-rational design with low throughput (< 300 colonies)	Titre: 265 µg·L^{-1} (TON < 35); Diastereoselectivity > 59%	32
Carbene insertion	CYP119_Ir(Me)MPIX	Genetic / semi-rational design with medium throughput (> 5000 colonies)	TON = 21600 (1.5-fold increase vs parent); e.e. < 43 %	33
Carbene insertion	CYP119_Ir(Me)MPIX	Genetic / semi-rational design with low throughput (< 1000 colonies)	TON > 1300; diastereoselectivity > 75% (1.5-fold increase vs parent)	34
RCM	HaloTag–SNAPTag_ Alkyl-chloride-(NHC)Ru	Genetic/ selection of two variants from previous study	TON_{DAPB} = 138 (1.2-fold increase vs parent);	45

			TON_{EnDA} = 293 (1.2-fold increase vs parent)	
RCM	dnTRP_(SulfaNHC)Ru	Genetic / directed evolution with medium throughput (> 5000 colonies)	TON up to 1.1 × 10^6 per cell (> 10-fold increase vs parent)	This work
In plasma or body fluids: moderate GSH content				
RCM	HSA_(Coumarin-NHC)Ru	Glycosylation of HSA scaffold	Decreased immunological effects	19 and 43

hCA: human carbonic anhydrase; NB4: Nitrobindin protein 4; Sav: streptavidin; CYP119: cytochrome P450 119; LmrR: *Lactococcal* multidrug resistance regulator; HSA: Human serum albumin; TON: turnover number; e.e.: enantiomeric excess; Subscript in TON: TON for specific for substrate. *References are numbered as shown in the main text

Supplementary Table 2 Primers used for site-directed (SDM) and site-saturation mutagenesis (SSM) PCRs.

The BsaI restriction sequence within the primers used for Goldengate assembly is highlighted in green.

Name of primer	Sequence (5'-3')
Fw SDM F43W	GAACAGGCGGCGTGGATTTTGTCGGTTG
Rev SDM F43W	ACGCCGCCTGTTCCAAGC
Fw SDM F81W	CTTGTTGATCCTGTGGGCGGCGGCCG
Rev SDM F81W	ACAGGATCAACAAGGCTTGCTCC
Fw SDM F116W	CTGTTAATCTTATGGACCGCGGCGGAATTG
Rev SDM F116W	ATAAGATTAACAGTGCTTGTTCTAAAC
Fw SSM E4	CGGGTCTCGCCTG**NNK**CAGGCGCTGCTGATTC
Rev SSM E4	CGGGTCTCCCAGGCTCACGCCAG
Fw SSM Q5	CGGGTCTCGCCTGGAA**NNK**GCGCTGCTGATTC
Rev SSM Q5	CGGGTCTCCCAGGCTCACGCCAG
Fw SSM E39	CGGGTCTCTCAGCTTG**NNK**CAGGCGGCGTTTAT
Fw SSM E39 (F43R)	CGGGTCTCACAGCTTG**NNK**CAGGCGGCGA
Rev SSM E39	CGGGTCTCAGCTGACGCCCAGTTTGG
Fw SSM F43	CGGGTCTCAGGCGGCG**NNK**ATTTTGTCGGTTG
Rev SSM F43	CGGGTCTCCCGCCTGTTCCAAGCTGAC
Fw SSM Q75	CGGGTCTCCGTTGGAG**NNK**GCCTTGTTGATCC
Rev SSM Q75	CGGGTCTCCCAACGAAACACCCAACTTC
Fw SSM L78	CGGGTCTCAAGCCTTG**NNK**ATCCTGTTTGCG
Rev SSM L78	CGGGTCTCAGGCTTGCTCCAACGAAAC
Fw SSM E109	GTGAGTTTA**NNK**CAAGCACTGTTAATCTTA
Rev SSM E109	GTGCTTG**MNN**TAAACTCACACCCAG
Fw SSM L113	CGGTCTCAAGCACTG**NNK**ATCTTATTTACC
Rev SSM L113	CGGTCTCGTGCTTGTTCTAAACTCACA
Fw SSM E144	CGGGTCTCTTAGTCTG**NNK**CAGGCGCAGG
Rev SSM E144	CGGGTCTCGACTAACGCCTAATTTCGTCTTC
Fw SSM A148	CGGTCTCGGCGCAG**NNK**ATTCTGGTGGT
Rev SSM A148	CGGTCTCGCGCCTGCTCCAGACTAACG
Fw SSM E179	GTCTCGTTA**NNK**CAAGCTTTGCTCATC
Rev SSM E179	CAAAGCTTG**MNN**TAACGAGACACCGAG
Fw SSM Q180	GGGTCTCCGTTAGAG**NNK**GCTTTGCTCATCC
Rev SSM Q180	CGGGTCTCCTAACGAGACACCGAGCTTCG

Fw SSM L183	CGGTCTCAAGCTTTG**NNK**ATCCTGGAAGTG
Rev SSM I183	CGGTCTCAAGCTTGCTCTAACGAGAC

Supplementary Table 3 Primers used for error-prone PCR and fragment shuffling. The *Bsa*I restriction sequence within the primers used for Goldengate assembly is highlighted in green.

Name of primer	Sequence (5'-3')
Fw epPCR insert	ATGGTCTCTAAGGTGGCGGGTCTG
Rev epPCR insert	TAGGTCTCTGTGGTGCTCGAGTTAGC
Fw epPCR vector backbone	TAGGTCTCTCCACCACCACCACCACT
Rev epPCR vector backbone	TAGGTCTCACCTTGAAAATACAGATTTTCGC
Fw Fra1	GAGATATACATATGGGCCATCATCACCAC
Fw Fra2	GTTAAAAACCAAACTGGGCGTCAGC
Fw Fra3	CTTGTTGATCCTGTTTGCGGCG
Fw Fra4	GTACTACGGTTGAGGAAGCAGTG
Fw Fra5	CTGAAATTAAAGACGAAGCTCGGTGTC
Fw Backbone	GAGATCCGGCTGCTAACAAAGC
Rev Fra1	CGCCCAGTTTGGTTTTTAACCATAACG
Rev Fra2	CGCCGCAAACAGGATCAACAAG
Rev Fra3	ACTGCTTCCTCAACCGTAGTAC
Rev Fra4	ACCGAGCTTCGTCTTTAATTTCAGG
Rev Fra5	TTGTTAGCAGCCGGATCTCAGTG
Rev Backbone	GATGGCCCATATGTATATCTCCTTCTTAAA

Supplementary Table 4 Primers used for generation of L8X, L113X, A148X, L183X and their corresponding recombined variants (X represents any amino acid residues except cysteine and proline).

Name of primer	Sequence (5'-3')
Fw L8A (E4G)	GGCAGGCGCTGGCGATTCTGAGCGTGGC
Fw L8D (E4G)	GGCAGGCGCTGGATATTCTGAGCGTGGCC
Fw L8E (E4G)	GGCAGGCGCTGGAAATTCTGAGCGTGGCC
Fw L8F (E4G)	GGCAGGCGCTGTTTATTCTGAGCGTGGCC
Fw L8G (E4G)	GGCAGGCGCTGGGGATTCTGAGCGTGGCC
Fw L8H (E4G)	GGCAGGCGCTGCATATTCTGAGCGTGGCC
Fw L8I (E4G)	GGCAGGCGCTGATTATTCTGAGCGTGGCC
Fw L8K (E4G)	GGCAGGCGCTGAAAATTCTGAGCGTGGCC
Fw L8M (E4G)	GGCAGGCGCTGATGATTCTGAGCGTG
Fw L8N (E4G)	GGCAGGCGCTGAACATTCTGAGCGTGGCC
Fw L8Q (E4G)	GGCAGGCGCTGCAGATTCTGAGCGTG
Fw L8R (E4G)	GGCAGGCGCTGCGTATTCTGAGCGTGGCC
Fw L8S (E4G)	GGCAGGCGCTGAGCATTCTGAGCGTGGCC
Fw L8T (E4G)	GGCAGGCGCTGACCATTCTGAGCGTGGCC
Fw L8V (E4G)	GGCAGGCGCTGGTGATTCTGAGCGTG
Fw L8W (E4G)	GGCAGGCGCTGTGGATTCTGAGCGTGGCC
Fw L8Y (E4G)	GGCAGGCGCTGTATATTCTGAGCGTGGCC
Rev L8X general	CAGCGCCTGCCCCAGGCTCAC
Fw L113A (F116W)	GAACAAGCACTGGCAATCTTATGGACCGCG

Fw L113D (F116W)	GAACAAGCACTGGATATCTTATGGACCGCG
Fw L113E (F116W)	GAACAAGCACTGGAAATCTTATGGACCGCG
Fw L113F (F116W)	GAACAAGCACTGTTTATCTTATGGACCGCG
Fw L113G (F116W)	GAACAAGCACTGGGAATCTTATGGACCGCG
Fw L113H (F116W)	GAACAAGCACTGCATATCTTATGGACCGCG
Fw L113I (F116W)	GAACAAGCACTGATTATCTTATGGACCGCG
Fw L113K (F116W)	GAACAAGCACTGAAAATCTTATGGACCGCG
Fw L113M (F116W)	GAACAAGCACTGATGATCTTATGGACCGCG
Fw L113N (F116W)	GAACAAGCACTGAACATCTTATGGACCGCG
Fw L113Q (F116W)	GAACAAGCACTGCAGATCTTATGGACCGCG
Fw L113R (F116W)	GAACAAGCACTGCGTATCTTATGGACCGCG
Fw L113S (F116W)	GAACAAGCACTGAGCATCTTATGGACCGCG
Fw L113T (F116W)	GAACAAGCACTGACCATCTTATGGACCGCG
Fw L113V (F116W)	GAACAAGCACTGGTGATCTTATGGACCGCG
Fw L113W (F116W)	GAACAAGCACTGTGGATCTTATGGACCGCG
Fw L113Y (F116W)	GAACAAGCACTGTATATCTTATGGACCGCG
Rev L113X general	CAGTGCTTGTTCTAAACTCACACC
Fw A148D	GGCAGGCGCAGGATATTCTGGTGGTTGCC
Fw A148E	GGCAGGCGCAGGAAATTCTGGTGGTTGCC
Fw A148F	GGCAGGCGCAGTTTATTCTGGTGGTTGCC
Fw A148G	GGCAGGCGCAGGGGATTCTGGTGGTTGCC
Fw A148H	GGCAGGCGCAGCATATTCTGGTGGTTGCC
Fw A148I	GGCAGGCGCAGATTATTCTGGTGGTTGCC
Fw A148K	GGCAGGCGCAGAAGATTCTGGTGGTTGCC
Fw A148L	GGCAGGCGCAGCTGATTCTGGTGGTTGCC
Fw A148M	GGCAGGCGCAGATGATTCTGGTGGTTGCC
Fw A148N	GGCAGGCGCAGAACATTCTGGTGGTTGCC
Fw A148Q	GGCAGGCGCAGCAGATTCTGGTGGTTGCC
Fw A148R	GGCAGGCGCAGCGTATTCTGGTGGTTGCCG
Fw A148S	GGCAGGCGCAGAGCATTCTGGTGGTTGCCG
Fw A148T	GGCAGGCGCAGACCATTCTGGTGGTTGCCG
Fw A148V	GGCAGGCGCAGGTTATTCTGGTGGTTGCCG
Fw A148W	GGCAGGCGCAGTGGATTCTGGTGGTTGCCG
Fw A148Y	GGCAGGCGCAGTATATTCTGGTGGTTGCCG
Rev A148X (E144G) general	CTGCGCCTGCCCCAGACTAACG
Fw L183A	GGACAAGCTTTGGCCATCCTGGAAGTGGCGG
Fw L183D	GGACAAGCTTTGGATATCCTGGAAGTGGCGG
Fw L183E	GGACAAGCTTTGGAAATCCTGGAAGTGGCGG
Fw L183F	GGACAAGCTTTGTTCATCCTGGAAGTGGCG
Fw L183G	GGACAAGCTTTGGGCATCCTGGAAGTGGCGG
Fw L183H	GGACAAGCTTTGCACATCCTGGAAGTGGC
Fw L183I	GGACAAGCTTTGATCATCCTGGAAGTGGC
Fw L183K	GGACAAGCTTTGAAAATCCTGGAAGTGGCGGC
Fw L183M	GGACAAGCTTTGATGATCCTGGAAGTGGCGG
Fw L183N	GGACAAGCTTTGAACATCCTGGAAGTGGCGG
Fw L183Q	GGACAAGCTTTGCAGATCCTGGAAGTGGCGG
Fw L183R	GGACAAGCTTTGCGTATCCTGGAAGTGGCGG
Fw L183S	GGACAAGCTTTGAGCATCCTGGAAGTGGCGG
Fw L183T	GGACAAGCTTTGACCATCCTGGAAGTGGCGG

Fw L183V	GGACAAGCTTTGGTCATCCTGGAAGTGGCG
Fw L183W	GGACAAGCTTTGTGGATCCTGGAAGTGGCGGC
Fw L183Y	GGACAAGCTTTGTATATCCTGGAAGTGGCGGC
Rev L183X (E179G) general	CAAAGCTTGTCCTAACGAGACAC

Supplementary Table 5. Statistical data for the X-ray crystal structures of *apo* dnTRP_R0-Δhis, Ru1·R0-Δhis and Ru1·R5-Δhis. The data have been deposited under PDB: 9GVF, 8S6P, and 9H3C, respectively.

PDB	9GVF	8S6P	9H3C
**Data Processing Statistics**			
Resolution Range (Å)	40.42 -1.60 (1.65-1.60)	45.10-2.90 (45.10-2.90)	73.50-2.90 (3.00-2.90)
Cell Parameters<br>- a, b, c (Å)<br>- α, β, γ (°)	 36.28, 60.38, 54.52<br>90.00, 93.42, 90.00	 53.17, 85.14, 90.24<br>90.00, 90.00, 90.00	 91.28, 91.28, 123.95<br>90.00, 90.00, 90.00
Space group	P 1 21 1	I 2 2 2	I 4_1 2 2
Total reflections	104850 (5258)	127544 (127544)	147491 (15393)
Unique reflections	30949 (1519)	4786 (4786)	5750 (598)
Rmerge (%)	0.032 (0.359)	0.1293 (0.1293)	14.7 (44.4)
Multiplicity	3.4 (3.5)	26.6 (26.6)	25.7 (25.7)
Mean I/Sig(I)	12.9 (2.4)	10.79 (10.79)	15.1 (0.63)
Completeness (%)	99.6 (99.8)	99.64 (99.64)	93.8 (99.0)
CC (1/2)	0.998 (0.875)	1.00 (1.00)	1.00 (0.543)
**Structure Refinement Statistics**			
R_{work}/R_{free}	0.20/0.25	0.26/0.32	0.25/0.32
RMS deviation<br>-Bond length (Å)<br>-Bond angles (°)<br>-Ramachandran favored (%)	 0.011<br>1.81<br>99.52	 0.008<br>1.75<br>98.57	 0.081<br>1.82<br>97.7
Average B-factors (Å^2)<br>-Protein<br>-Ligands<br>-solvent	25.37<br>25<br>-<br>33.70	143.30<br>141.57<br>205.33 	123<br>123<br>125<br>-

Supplementary Appendix

DNA and corresponding protein sequence of the dnTRP designs.

The DNA sequences (including 5'-terminal hexa-histidine, TEV protease cleavage site, and linkers) are labeled in light red. The exact DNA sequences of the dnTRP designs are highlighted in bold. The protein sequences (including N-terminal hexa-histidine and TEV protease cleavage sequence) are labeled in green. The exact protein sequences of the dnTRP designs are highlighted in bold.

**Name of dnTRP**	DNA/protein **sequence**
**dnTRP_01**	ATGGGCCATCATCACCACCATCATGGCAGCGGCAGTGGCGAAAATCTGTATTTTCAAGGTGGCGGCTCGGGC**GTGAGCCTG**<br>**ATTCAGGCGGCGTTTGCGCTGCTGACCGCGGCCAATCTGGGCACCACCGTGGAAGAAGCGGTGAAACGCGCGTTATGGTT**<br>**AAAAACCAAACTGGGTGTCAGCTTGTATCAGGCGTTTACCATTCTGAATGTGGCTGCGTATCTGGGTACCACGGTAGAGGA**<br>**GGCCGTTAAGCGCGCGCTGAAACTGAAAACGAAGTTGGGCGTTAGCCTGTTTCAGGCATTTCAGATTCTGCAGGTGGCCG**<br>**CCTTTTTGGGTACGACGGTTGAGGAAGCTGTAAAGCGTGCCTTGAAGTTGAAGACGAAATTGGGTGTGAGTCTGGAACAG**<br>**GCCTTAAAAATTCTGCTGGCAGCCGCGTTCTTGGGGACGACCGTTGAGGAGGCCGTTAAGCGTGCACTGAAATTAAAGAC**<br>**TAAGCTCGGTGTTTCGTTGGAGCAAGCATTGCTGATCTTGCTGGTGGCGTGGCAGCTGGGGACCACTGTCGAAGAGGCAG**<br>**TGTATCGCGCCCTCAAGCTCAAGACCAAGTTAGGGGTGAGTTTAGAACAGGCCCTGATTATTTTAGCGGCTGCGGCGCGC**<br>**CTGGGGACTACGGTTGAGGAGGCCGTTAAACGCGCCTTGAAGTTGAAGACGAAGCTGGGCTAA**<br>MGHHHHHHGSGSGENLYFQGGGSG**VSLIQAAFALLTAANLGTTVEEAVKRALWLKTKLGVSLYQAFTILNVAAYLGTTVEEAVK**<br>**RALKLKTKLGVSLFQAFQILQVAAFLGTTVEEAVKRALKLKTKLGVSLEQALKILLAAAFLGTTVEEAVKRALKLKTKLGVSLEQ**<br>**ALLILLVAWQLGTTVEEAVYRALKLKTKLGVSLEQALIILAAAARLGTTVEEAVKRALKLKTKLG**
**dnTRP_02**	ATGGGCCATCATCACCACCATCATGGCAGCGGCAGTGGCGAAAATCTGTATTTTCAAGGTGGTGGTTCGGGC**GTGGATCTGT**<br>**GGCAGGCGCTGGCGATTGCGGCCTTAGCGGCGGAACTGGGCACCACCGTGGAAGAAGCGGTGAAACGCGCGCTGTGGC**<br>**TGAAAACCAAATTGGGCGTGAGCTTTGATATTGCGATCGCAATTCTGTGGGTTGCAGCAAAATTAGGTACGACCGTTGAGG**<br>**AGGCCGTTAAGCGTGCGCTGAAACTGAAGACGAAGTTGGGTGTGAGCCTGTTTACCGCGCTGCTGATTGCGCTGGTTGCC**<br>**GCGATGCTGGGTACTACGGTTGAAGAGGCGGTAAAGCGCGCCTTGAAGCTGAAGACGAAGCTTGGTGTTAGCCTGGAAC**<br>**AGGCATTGTTAATTCTGCTGCAGGCGGCGCAGTTGGGCACGACTGTGGAGGAAGCTGTTAAACGTGCATTGAAACTCAAG**<br>**ACTAAACTGGGGGTTTCGCTTGAGCAAGCTCTGGAAATTCTGAGCGTGGCCGCCGAGCTGGGGACGACGGTCGAAGAGG**<br>**CAGTGTATCGCGCCCTCAAGCTTAAAACTAAGCTCGGCGTGTCTTTGGAGCAGGCCTTGTGGATCCTGTTTGTTGCTGCCA**<br>**AGCTTGGTACCACTGTTGAGGAGGCCGTCAAACGTGCACTGAAATTAAAAACGAAACTGGGCTAA**<br>MGHHHHHHGSGSGENLYFQGGGSG**VDLWQALAIAALAAELGTTVEEAVKRALWLKTKLGVSFDIAIAILWVAAKLGTTVEEAVK**<br>**RALKLKTKLGVSLFTALLIALVAAMLGTTVEEAVKRALKLKTKLGVSLEQALLILLQAAQLGTTVEEAVKRALKLKTKLGVSLEQA**<br>**LEILSVAAELGTTVEEAVYRALKLKTKLGVSLEQALWILFVAAKLGTTVEEAVKRALKLKTKLG**
**dnTRP_03**	ATGGGCCATCATCACCACCATCATGGCAGCGGCAGTGGCGAAAATCTGTATTTTCAAGGTGGCGGGTCGGGC**GTGAGCCTG**<br>**CAGCAGGCGGCGTTTATTCTGATTCTGGCCGCGTTTCTGGGCACCACCGTGGAAGAAGCGGTGAAACGCGCGTTGTGGTT**<br>**GAAAACCAAACTGGGCGTAAGCCTGACCATTGCGGCGCTGATTCTGAATGCGGCGGTGGAATTGGGTACGACGGTAGAG**<br>**GAGGCCGTTAAGCGCGCGCTGAAACTGAAAACGAAGTTGGGTGTTAGTCTGGAACAGGCCTTAAAAATTTTGTTAGTTGCC**<br>**GCAGCACTGGGTACCACGGTTGAGGAAGCTGTTAAACGTGCCCTCAAGTTGAAGACGAAATTGGGGGTTTCGTTGGAGCA**<br>**AGCCCTGAAAATTCTGCTGGCGGCTGCCTTCTTGGGGACCACTGTTGAGGAGGCAGTGAAGCGTGCTTTGAAGCTGAAGA**<br>**CCAAATTAGGCGTATCGTTAGAACAAGCACTGCATATTCTGTTTGTGGCCTTTCTGTTGGGCACTACGGTCGAGGAGGCAG**<br>**TGTACCGCGCCTTGAAATTGAAGACTAAACTTGGTGTGTCTCTGGAAGTTGCGACCAGCATTCTGACCACCGCGGCATTTT**<br>**TGGGTACTACCGTCGAAGAGGCCGTAAAGCGTGCCTTGAAGTTAAAAACGAAGCTGGGCTAA**<br>MGHHHHHHGSGSGENLYFQGGGSG**VSLQQAAFILILAAFLGTTVEEAVKRALWLKTKLGVSLTIAALILNAAVELGTTVEEAVKR**<br>**ALKLKTKLGVSLEQALKILLVAAALGTTVEEAVKRALKLKTKLGVSLEQALKILLAAAFLGTTVEEAVKRALKLKTKLGVSLEQAL**<br>**HILFVAFLLGTTVEEAVYRALKLKTKLGVSLEVATSILTTAAFLGTTVEEAVKRALKLKTKLG**
**dnTRP_04**	ATGGGCCATCATCACCACCATCATGGCAGCGGCAGTGGCGAAAATCTGTATTTTCAAG**GTGGCGGGTCGGGCGTGAGCCTG**<br>**TGGCAGGCGCTGGCGATTTTAGCGGTTGCCGCGCTGCTGGGTACCACCGTTGAAGAAGCGGTTAAACGTGCGTTATGGTT**<br>**AAAAACCAAACTGGGTGTTAGCTTGGAACAGGCGCAGAGCATTCTGGCCGCCGCAGCGTTTCTGGGCACCACGGTCGAG**<br>**GAGGCTGTCAAGCGCGCGCTGAAACTGAAAACGAAGTTGGGCGTTAGCCTGGAACAAGCATTGAATATTCTGAATGTGGC**<br>**CGCGCAGTTGGGGACGACGGTGGAAGAGGCCGTGAAACGCGCTTTGAAGTTAAAGACGAAATTAGGCGTTTCGCTGTATC**<br>**AGGCCTTGGCAATTCTGCAGGTGGCTGCGGTTTTAGGCACTACCGTAGAGGAAGCAGTTAAGCGTGCCTTGAAGCTCAAG**<br>**ACCAAGCTCGGTGTGTCTTTGGAGCAAGCTCTGCTGATTCTGTTTGTTGCGGCCGCCTTGGGTACGACTGTTGAGGAGGCC**<br>**GTGTATCGCGCCCTCAAGCTGAAGACTAAATTGGGTGTCTCGTTGGAGCAAGCCCTGGTGATCCTCGCCGTTGCAGCTTTG**<br>**TTAGGGACTACGGTTGAAGAGGCCGTAAAGCGTGCACTGAAATTAAAGACTAAACTTGGCTAA**<br>MGHHHHHHGSGSGENLYFQGGGSG**VSLWQALAILAVAALLGTTVEEAVKRALWLKTKLGVSLEQAQSILAAAAFLGTTVEEAV**<br>**KRALKLKTKLGVSLEQALNILNVAAQLGTTVEEAVKRALKLKTKLGVSLYQALAILQVAAVLGTTVEEAVKRALKLKTKLGVSLE**<br>**QALLILFVAAALGTTVEEAVYRALKLKTKLGVSLEQALVILAVAALLGTTVEEAVKRALKLKTKLG**
**dnTRP_06**	ATGGGCCATCATCACCACCATCATGGCAGCGGCAGTGGCGAAAATCTGTATTTTCAAGGTGGCGGCTCGGGC**GTGAGCCTG**<br>**GAACAGGCGCTGCTGATTCTGCTGGTTGCCGCGCATCTGGGCACCACCGTGGAAGAAGCGGTGAAACGCGCGTTATGGTT**<br>**AAAAACCAAACTGGGCGTTTCGTTGGAGCAGGCCGCCCAAATTCTGGCGATTGCGGCCCACTTGGGTACGACGGTTGAGG**<br>**AGGCCGTTAAACGCGCGCTGAAACTGAAAACGAAGTTGGGTGTTTCGCTTGAGCAAGCGTTGAATATTTTAGCGGTGGCG**<br>**CGCCTGCTGGGTACTACTGTGGAGGAAGCAGTGAAGCGTGCTCTCAAGTTGAAGACGAAATTAGGTGTTAGCCTGCATCA**<br>**GGCACTGGAAATTTTAGCGCTGGCGGCCCTGTTGGGGACGACCGTCGAAGAGGCTGTTAAGCGTGCCTTGAAGCTGAAGA**<br>**CCAAATTGGGCGTCTCCCTTGAACAAGCCGTGCAGATTTTGGCCGTCGCGTATGCGTTAGGGACCACGGTCGAGGAGGCA**<br>**GTGTATCGCGCTTTGAAATTGAAAACTAAGTTAGGGGTGTCTTTGGAACAAGCACTGTATATTCTCGCTGTTGCGGCGCAG**<br>**TTAGGCACGACTGTAGAGGAGGCCGTCAAACGTGCACTGAAGCTTAAAACCAAGCTGGGCTAA**

	GHHHHHHGSGSGENLYFQGGGSG**VSLEQALLILLVAAHLGTTVEEAVKRALWLKTKLGVSLEQAAQILAIAAHLGTTVEEAVKR**<br>**ALKLKTKLGVSLEQALNILAVARLLGTTVEEAVKRALKLKTKLGVSLHQALEILALAALLGTTVEEAVKRALKLKTKLGVSLEQAV**<br>**QILAVAYALGTTVEEAVYRALKLKTKLGVSLEQALYILAVAAQLGTTVEEAVKRALKLKTKLG**
**dnTRP_07**	ATGGGCCATCATCACCACCATCATGGCAGCGGCAGTGGCGAAAATCTGTATTTTCAAGGTGGCGGCTCGGGC**GTGAGCCTG**<br>**GAACAGGCGGCCTGGATTCTGAGCGTGGCCGCGGAACTGGGTACCACCGTTGAAGAAGCGGTGAAACGTGCGTTATGGT**<br>**TAAAAACCAAACTGGGCGTCAGCTTGACCCAGGCGATCCTGATTCTGCTGATTGCGGCGTATCTGGGCACCACGGTGGAA**<br>**GAGGCGGTGTATCGCGCGCTGAAACTGAAAACGAAGCTCGGTGTGTCGCTGGTGCAGGCGCTGATTATTTTGCTGACCGC**<br>**GGCGCTGCTGGGGACGACGGTTGAGGAGGCCGTTAAGCGCGCCTTGAAGTTAAAGACGAAGTTGGGTGTTTCGTTGGATC**<br>**AGGCCCTGTTAATTTTAGCGGCCGCTGCATGGTTGGGCACGACTGTCGAAGAGGCAGTAAAGCGTGCCCTCAAGCTTAAG**<br>**ACCAAATTAGGCGTTAGTCTGGAGCAGGCTCTGAAAATTCTGCATGTGGCTGCCGAGTTAGGCACTACCGTCGAGGAAGC**<br>**TGTATACCGTGCCTTAAAATTAAAGACTAAACTTGGCGTTTCGCTGGAGCAAGCTCTGCAGATCCTGCGCCTGGCCGCCAA**<br>**TTTAGGTACGACTGTAGAAGAGGCCGTGAAGCGCGCACTGTATCTGAAGACTAAACTCGGCTAA**<br>MGHHHHHHGSGSGENLYFQGGGSG**VSLEQAAWILSVAAELGTTVEEAVKRALWLKTKLGVSLTQAILILLIAAYLGTTVEEAVYR**<br>**ALKLKTKLGVSLVQALIILLTAALLGTTVEEAVKRALKLKTKLGVSLDQALLILAAAAWLGTTVEEAVKRALKLKTKLGVSLEQAL**<br>**KILHVAAELGTTVEEAVYRALKLKTKLGVSLEQALQILRLAANLGTTVEEAVKRALYLKTKLG**
**dnTRP_08**	ATGGGCCATCATCACCACCATCATGGCAGCGGCAGTGGCGAAAATCTGTATTTTCAAGGTGGCGGCTCGGGC**GTGAGCCTG**<br>**TATCAGGCGCTGGAAATTCTGCTGGTTGCCGCGGAACTGGGCACCACCGTGGAAGAAGCGGTGAAACGCGCGTTATGGTT**<br>**AAAAACCAAATTGGGCGTTAGCCTGCATCAAGCGATTCTGATTTTACTGTATGCGGCGATTGTGGGCACGACGGTTGAGGA**<br>**GGCAGTGTATCGCGCGCTGAAACTGAAAACGAAGCTCGGTGTTAGTCTGGAACAGGCGATTTATATTCTGCAGGTTGCGCT**<br>**GATTCTGGGTACTACCGTAGAGGAAGCCGTAAAGCGCGCCTTGAAGTTGAAGACCAAGTTGGGTGTGTCGTTGGAGCAGG**<br>**CCTTAAAAATCTTAATTACCGCGGCGGCCCTGGGGACGACTGTGGAGGAGGCTGTTAAGCGTGCCCTGAAATTAAAGACG**<br>**AAACTGGGGGTTTCGCTTGAGCAAGCCGTGTTGATCCTGGCGGCAGCCTGGTTTTTAGGCACCACGGTCGAAGAGGCCGT**<br>**TTACCGTGCGTTAAAGCTCAAGACGAAATTAGGCGTCAGCTTGGAACAAGCCCTGATTATTTTGGCGGTGGCCGCGAAATT**<br>**AGGTACCACTGTAGAAGAGGCCGTCAAACGTGCACTGTATCTGAAGACTAAACTTGGCTAA**<br>MGHHHHHHGSGSGENLYFQGGGSG**VSLYQALEILLVAAELGTTVEEAVKRALWLKTKLGVSLHQAILILLYAAIVGTTVEEAVYR**<br>**ALKLKTKLGVSLEQAIYILQVALILGTTVEEAVKRALKLKTKLGVSLEQALKILITAAALGTTVEEAVKRALKLKTKLGVSLEQAVLI**<br>**LAAAWFLGTTVEEAVYRALKLKTKLGVSLEQALIILAVAAKLGTTVEEAVKRALYLKTKLG**
**dnTRP_09**	ATGGGCCATCATCACCACCATCATGGCAGCGGCAGTGGCGAAAATCTGTATTTTCAAGGTGGCGGGTCTGGC**GTGAGCCTG**<br>**TATCAGGCGGCGGCGATTTTAGCGGTTGCGGCAGCGCTGGGTACCACCGTTGAAGAAGCGGTGAAACGTGCGTTATGGTT**<br>**AAAAACCAAACTGGGCGTCAGCTTGGAACAGGCGATTCAGATTCTGGCGACCGCGGCGTTTCTGGGCACCACGGTGGAA**<br>**GAGGCAGTGTATCGCGCGCTGAAACTGAAAACGAAGTTGGGCGTTAGTCTGGAACAGGCGCTGAGCATTCTGGTGGTGGC**<br>**GTGGCTGTTGGGCACGACGGTTGAGGAGGCCGTTAAGCGCGCCTTGAAGTTAAAGACGAAATTGGGTGTTTCGTTGGAGC**<br>**AAGCACTGCAGATTCTGCAGGTGGCCGCTTTCTTGGGTACGACCGTGGAGGAAGCAGTAAAGCGTGCCCTGAAATTAAAG**<br>**ACTAAACTTGGCGTGTCCTTAGAACAAGCCCTGCTGATTCTGTTTGTGGCGGCCAATTTGGGGACGACTGTCGAAGAGGCT**<br>**GTTTACCGTGCACTGAAGTTGAAAACCAAGCTCGGTGTGAGTTTGGAGCAAGCGCTGTATATTTTGGCGATTGCGCGCGAA**<br>**GCGGGGACCACTGTAGAGGAGGCCGTCAAACGCGCTTTATATCTGAAGACTAAGTTAGGCTAA**<br>MGHHHHHHGSGSGENLYFQGGGSG**VSLYQAAAILAVAAALGTTVEEAVKRALWLKTKLGVSLEQAIQILATAAFLGTTVEEAVY**<br>**RALKLKTKLGVSLEQALSILVVAWLLGTTVEEAVKRALKLKTKLGVSLEQALQILQVAAFLGTTVEEAVKRALKLKTKLGVSLEQ**<br>**ALLILFVAANLGTTVEEAVYRALKLKTKLGVSLEQALYILAIAREAGTTVEEAVKRALYLKTKLG**
**dnTRP_10**	ATGGGCCATCATCACCACCATCATGGCAGCGGCAGTGGCGAAAATCTGTATTTTCAAGGTGGCGGGTCGGGC**GTGAGCCTG**<br>**GAACAGGCGCTGTATGTTTTAGCGCTGGCGGCGCTGCTGGGCACCACCGTGGAAGAAGCGGTGAAACGCGCGTTATGGTT**<br>**AAAAACCAAACTGGGCGTTAGCCTGAAACAGGCCCTGTTGATTGCGGCGACCGCGGCGGAATTGGGCACGACGGTTGAA**<br>**GAGGCCGTTAAACGCGCGCTGAAACTGAAAACGAAGTTGGGTGTTAGTCTGTTTCAGGCGGCGTTGATTCTGCTGGTTGC**<br>**GGCAAAATTGGGTACGACTGTCGAGGAGGCAGTGAAGCGTGCCCTCAAGCTCAAGACGAAATTAGGTGTTTCGTTGGAGC**<br>**AAGCCCTGGCGATTCTGTATATTGCAGCGCAGTTAGGCACTACCGTTGAGGAAGCCGTCAAACGTGCTCTCAAGTTAAAGA**<br>**CTAAACTCGGCGTCAGTTTAGAACAGGCGTTTGTGATTTTGGCGGTGGCTGCGCGCCTGGGTACCACGGTAGAGGAGGCA**<br>**GTGTATCGCGCTTTGAAGCTGAAGACCAAGCTCGGTGTGAGTCTTGAGCAGGCTCTGGTGATTCTCTTGGTTGCCGCCCAT**<br>**CTGGGGACTACGGTGGAGGAGGCCGTTAAGCGTGCACTGAAATTAAAGACCAAGCTCGGCTAA**<br>MGHHHHHHGSGSGENLYFQGGGSG**VSLEQALYVLALAALLGTTVEEAVKRALWLKTKLGVSLKQALLIAATAAELGTTVEEAV**<br>**KRALKLKTKLGVSLFQAALILLVAAKLGTTVEEAVKRALKLKTKLGVSLEQALAILYIAAQLGTTVEEAVKRALKLKTKLGVSLEQ**<br>**AFVILAVAARLGTTVEEAVYRALKLKTKLGVSLEQALVILLVAAHLGTTVEEAVKRALKLKTKLG**
**dnTRP_11**	ATGGGCCATCATCACCACCATCATGGCAGCGGCAGTGGCGAAAATCTGTATTTTCAAGGTGGCGGCTCGGGC**GTGAGCCTG**<br>**GAACAGGCGCTGAATACCTTAGCGGCGGCCGCGGAACTGGGCACCACCGTGGAAGAAGCGGTGAAACGCGCGTTATGGT**<br>**TAAAAACCAAATTGGGCGTTTCGCTTGAGCAGGCGTTGGCGATTTTGGTTGCGGCTGCGCAGCTGGGTACGACGGTTGAA**<br>**GAGGCCGTTAAACGTGCCTTGAAACTGAAAACGAAGTTGGGTGTTTCGTTGGATGTGGCGGTGGCCATTCTGATTGCAGCC**<br>**GCCCATCTGGGGACTACCGTTGAGGAGGCTGTAAAGCGTGCTTTGAAGTTAAAGACGAAATTAGGCGTTAGTCTGCTGCAG**<br>**GCACTGCTGATTATTATTCTGGCGGCCCAATTGGGTACCACTGTCGAAGAGGCAGTGAAGCGCGCCCTCAAATTAAAGACT**<br>**AAGCTGGGCGTGTCTTTAGATCAGGCTCTGAAAATTCTGAGCGTGGCCGCCAATCTGGGTACGACCGTGGAGGAAGCAGT**<br>**GTATCGCGCACTTAAATTGAAGACCAAGTTAGGTGTTAGCCTCGAACAAGCGTTGATTATCCTGGAAGTGGCAGCAAAACT**<br>**GGGGACGACGGTAGAGGAGGCCGTCAAGCGTGCATTAAAACTTAAAACGAAGCTTGGCTAA**<br>MGHHHHHHGSGSGENLYFQGGGSG**VSLEQALNTLAAAAELGTTVEEAVKRALWLKTKLGVSLEQALAILVAAAQLGTTVEEAV**<br>**KRALKLKTKLGVSLDVAVAILIAAAHLGTTVEEAVKRALKLKTKLGVSLLQALLIIILAAQLGTTVEEAVKRALKLKTKLGVSLDQA**<br>**LKILSVAANLGTTVEEAVYRALKLKTKLGVSLEQALIILEVAAKLGTTVEEAVKRALKLKTKLG**
**dnTRP_12**	ATGGGCCATCATCACCACCATCATGGCAGCGGCAGTGGCGAAAATCTGTATTTTCAAGGTGGCGGCTCGGGC**GTGAGCCTG**<br>**GAACAGGCGTTGCGTACCCTGCTGGCGGCCGCGGAACTGGGTACCACCGTTGAAGAAGCGGTTAAACGTGCGTTATGGTT**<br>**AAAAACCAAATTGGGTGTTAGCTTGGCGCAAGCGTTGGCGATTTTGGTTACCGCGGCGCAACTGGGGACGACCGTGGAGG**<br>**AGGCTGTGAAGCGCGCCTTGAAATTGAAAACGAAGCTCGGCGTTTCGTTGGATGTGGCGGTGGCGATCTTAGCGGTTGCT**<br>**GCGGTGCTGGGCACCACGGTGGAAGAGGCCGTGAAACGCGCGCTGAAACTGAAAACTAAGTTGGGCGTCTCCCTGGAGC**<br>**AGGCGCTGGCCATTCTGGTTCTGGCCGCGCTGTTAGGTACGACTGTTGAGGAAGCTGTCAAGCGTGCATTAAAGCTGAAG**<br>**ACCAAGTTAGGTGTCAGTTTAGAACAAGCCTTTAAAATTCTGAGCGTGGCAGCCAATCTGGGCACCACTGTTGAGGAGGCC**<br>**GTGTATCGCGCCCTCAAGTTAAAGACGAAACTTGGTGTGTCTTTGGAACAGGCATTGCTGATCCTGGAAGTGGCGGCGAAA**<br>**TTAGGGACTACGGTCGAAGAGGCCGTCAAACGTGCGTTAAAACTTAAAACGAAGTTGGGCTAA**<br>MGHHHHHHGSGSGENLYFQGGGSG**VSLEQALRTLLAAAELGTTVEEAVKRALWLKTKLGVSLAQALAILVTAAQLGTTVEEAV**<br>**KRALKLKTKLGVSLDVAVAILAVAAVLGTTVEEAVKRALKLKTKLGVSLEQALAILVLAALLGTTVEEAVKRALKLKTKLGVSLEQ**<br>**AFKILSVAANLGTTVEEAVYRALKLKTKLGVSLEQALLILEVAAKLGTTVEEAVKRALKLKTKLG**

**dnTRP_13**	ATGGGCCATCATCACCACCATCATGGCAGCGGCAGTGGCGAAAATCTGTATTTTCAAGGTGGCGGCTCGGGC**GTGAGCCTG**<br>**GAACAGGCGCTGTGGATTTTAGCGGCGGCCGCGGAACTGGGCACCACCGTGGAAGAAGCGGTGAAACGCGCGTTATGGT**<br>**TAAAAACCAAATTGGGCGTTTCTTTGGAGCAGGCGGTGGCGATTTTGATTATTGCGGCGCAGCTGGGTACGACCGTTGAGG**<br>**AGGCCGTTAAGCGCGCGCTGAAACTGAAAACGAAGTTGGGTGTTTCGTTGGATACCGCGTTTGCGATTCTCGCGGTTGCCG**<br>**CGGCGTTGGGCACTACGGTCGAAGAGGCTGTTAAACGTGCCTTGAAGTTGAAGACGAAATTAGGTGTCAGCCTTGAGCAA**<br>**GCGCTGACCATTCTGATTCTGGCCGCGCTGTTAGGCACGACGGTTGAGGAGGCAGTGAAGCGTGCTTTGAAATTGAAAAC**<br>**CAAGCTCGGTGTATCGCTGGAGCAGGCGTTTAAAATTCTGAGCGTGGCAGCCAATCTGGGGACCACTGTTGAGGAGGCCG**<br>**TCTACCGCGCCCTTAAATTAAAGACCAAACTGGGTGTGAGTCTGGAGCAAGCATTGACGATCCTGGAAGTGGCCGCAAAA**<br>**CTCGGGACTACTGTGGAGGAAGCCGTAAAGCGCGCATTAAAGCTCAAGACTAAGTTGGGCTAA**<br>MGHHHHHHGSGSGENLYFQGGGSG**VSLEQALWILAAAAELGTTVEEAVKRALWLKTKLGVSLEQAVAILIIAAQLGTTVEEAVK**<br>**RALKLKTKLGVSLDTAFAILAVAAALGTTVEEAVKRALKLKTKLGVSLEQALTILILAALLGTTVEEAVKRALKLKTKLGVSLEQAF**<br>**KILSVAANLGTTVEEAVYRALKLKTKLGVSLEQALTILEVAAKLGTTVEEAVKRALKLKTKLG**
**dnTRP_14**	ATGGGCCATCATCACCACCATCATGGCAGCGGCAGTGGCGAAAATCTGTATTTTCAAGGTGGCGGCTCGGGC**GTGAGCCTG**<br>**GAACAGGCGCTGTTTATTCTGCTGGTTGCCGCGGAACTGGGCACCACCGTGGAAGAAGCGGTGAAACGCGCGTTATGGTT**<br>**AAAAACCAAATTGGGCGTTTCTTTAGAAGTGGCGCAGGCGATTCTGATTATTGCGGCGCAGCTGGGTACGACGGTTGAGG**<br>**AGGCCGTAAAGCGCGCGCTGAAACTGAAAACGAAGTTGGGTGTGTCGTTAGAAACCGCGAATGCGATTTTAGCGGTCGCG**<br>**GCGGCGTTGGGCACGACCGTTGAAGAGGCAGTTAAGCGTGCCCTCAAGCTGAAGACGAAACTGGGTGTAAGTCTGCTGCA**<br>**GGCCCTGGCCATTCTGCATGCCGCCGCACTGTTGGGGACTACCGTCGAGGAAGCAGTGAAGCGTGCCTTGAAATTGAAAA**<br>**CTAAATTAGGGGTTAGCTTGGAGCAGGCGTTTAAAATTCTGTTTGTGGCAGCCAATCTGGGCACGACTGTGGAGGAGGCTG**<br>**TGTATCGCGCCCTGAAGTTAAAGACTAAGCTCGGCGTCAGTCTTGAACAAGCACTGATCATCCTGGAGGTGGCTGCGAAAC**<br>**TCGGTACCACTGTCGAAGAGGCCGTGAAACGCGCATTGAAGCTCAAAACTAAGCTGGGCTAA**<br>MGHHHHHHGSGSGENLYFQGGGSG**VSLEQALFILLVAAELGTTVEEAVKRALWLKTKLGVSLEVAQAILIIAAQLGTTVEEAVKR**<br>**ALKLKTKLGVSLETANAILAVAAALGTTVEEAVKRALKLKTKLGVSLLQALAILHAAALLGTTVEEAVKRALKLKTKLGVSLEQAF**<br>**KILFVAANLGTTVEEAVYRALKLKTKLGVSLEQALIILEVAAKLGTTVEEAVKRALKLKTKLG**
**dnTRP_15**	ATGGGCCATCATCACCACCATCATGGCAGCGGCAGTGGCGAAAATCTGTATTTTCAAGGTGGCGGCTCGGGCGTGAGCCT**GT**<br>**GGCAGGCGCTGGCGATTCTGAGCGCGGCGGCGCATTTGGGTACCACCGTTGAAGAAGCGGTTAAACGTGCGTTATGGTTA**<br>**AAAACCAAATTGGGTGTTAGCTTGACCGAAGCGTTGACCATTCTGTTTGTGGCCGCGTATCTGGGCACCACGGTGGAAGAG**<br>**GCCGTGAAACGCGCGCTGAAACTGAAAACTAAACTGGGCGTTAGCCTGCTGCAAGCACTGCTGATTCTGCTGGTTGCAGC**<br>**GAAATTAGGCACGACGGTTGAGGAGGCGGTGAAGCGTGCCTTGAAGCTGAAGACGAAGTTGGGCGTCAGTCTGGATCAG**<br>**GCCCTCTTGATTTTGTTCGTTGCGGCGGAACTGGGTACTACTGTCGAAGAGGCAGTAAAGCGCGCCCTCAAGTTAAAGACC**<br>**AAACTCGGCGTTTCGTTGGAACAGGCACTGGTGATTGCGACCGTGGCAGCCCAGCTGGGGACTACCGTGGAGGAAGCTGT**<br>**TTATCGCGCGTTGAAATTGAAAACGAAGCTCGGTGTGTCTTTGGAGCAAGCGCTGTTGATCCTGGCAGCTGCGAGCGAATT**<br>**GGGCACTACGGTCGAGGAGGCAGTCAAACGTGCTCTGAAATTAAAAACTAAACTTGGCTAA**<br>MGHHHHHHGSGSGENLYFQGGGSG**VSLWQALAILSAAAHLGTTVEEAVKRALWLKTKLGVSLTEALTILFVAAYLGTTVEEAVK**<br>**RALKLKTKLGVSLLQALLILLVAAKLGTTVEEAVKRALKLKTKLGVSLDQALLILFVAAELGTTVEEAVKRALKLKTKLGVSLEQA**<br>**LVIATVAAQLGTTVEEAVYRALKLKTKLGVSLEQALLILAAASELGTTVEEAVKRALKLKTKLG**
**dnTRP_16**	ATGGGCCATCATCACCACCATCATGGCAGCGGCAGTGGCGAAAATCTGTATTTTCAAGGTGGCGGGTCGGGC**GTGAGCCTG**<br>**GAACAGGCGCTGCAGATTCTGAGCGTGGCCGCGCAGCTGGGTACCACCGTTGAAGAAGCGGTGAAACGTGCGTTATGGTT**<br>**AAAAACCAAACTGGGCGTCAGCTTGGAACAGGCGTTGTTGATCTTGTCGGTTGCAGCGGAATTGGGCACCACGGTGGAAG**<br>**AGGCCGTTAAACGCGCGCTGAAACTGAAAACGAAGTTGGGCGTTTCGCTTGAGCAAGCCCTGCTGATCCTGTTTGCGGCG**<br>**GCAAAATTGGGTACGACGGTTGAGGAGGCAGTCAAGCGTGCCTTGAAGCTGAAGACGAAATTAGGTGTTTCGTTGGAGCA**<br>**AGCCTTGCTCATCCTGTATGTGGCGGCCGAGCTGGGGACGACCGTGGAGGAAGCTGTTAAGCGCGCTTTGAAACTCAAGA**<br>**CTAAACTCGGTGTTAGCCTGCTGCAGGCACTGATTATTCTGGTGATTGCGGCAGAATTAGGCACTACCGTAGAGGAGGCAG**<br>**TGTATCGCGCCTTAAAATTGAAGACCAAGCTCGGCGTATCGCTGGAAGCCGCGTTTCTGATTCTGGAAGTGGCTGCCAAGT**<br>**TAGGTACTACTGTGGAGGAGGCCGTAAAACGTGCACTGAAGTTGAAGACGAAACTGGGCTAA**<br>MGHHHHHHGSGSGENLYFQGGGSG**VSLWQALAILSAAAHLGTTVEEAVKRALWLKTKLGVSLTEALTILFVAAYLGTTVEEAVK**<br>**RALKLKTKLGVSLLQALLILLVAAKLGTTVEEAVKRALKLKTKLGVSLDQALLILFVAAELGTTVEEAVKRALKLKTKLGVSLEQA**<br>**LVIATVAAQLGTTVEEAVYRALKLKTKLGVSLEQALLILAAASELGTTVEEAVKRALKLKTKLG**
**dnTRP_17**	ATGGGCCATCATCACCACCATCATGGCAGCGGCAGTGGCGAAAATCTGTATTTTCAAGGTGGCGGGTCGGGC**GTGAGCCTG**<br>**GAACAGGCGCTGCAGATTCTGAGCGTGGCCGCGCAGCTGGGTACCACCGTTGAAGAAGCGGTGAAACGTGCGTTATGGTT**<br>**AAAAACCAAACTGGGCGTCAGCTTGGAACAGGCGTTGTTGATCTTGTCGGTTGCAGCGGAATTGGGCACCACGGTGGAAG**<br>**AGGCCGTTAAACGCGCGCTGAAACTGAAAACGAAGTTGGGCGTTTCGCTTGAGCAAGCCCTGCTGATCCTGTTTGCGGCG**<br>**GCAAAATTGGGTACGACGGTTGAGGAGGCAGTCAAGCGTGCCTTGAAGCTGAAGACGAAATTAGGTGTTTCGTTGGAGCA**<br>**AGCCTTGCTCATCCTGTATGTGGCGGCCGAGCTGGGGACGACCGTGGAGGAAGCTGTTAAGCGCGCTTTGAAACTCAAGA**<br>**CTAAACTCGGTGTTAGCCTGCTGCAGGCACTGATTATTCTGGTGATTGCGGCAGAATTAGGCACTACCGTAGAGGAGGCAG**<br>**TGTATCGCGCCTTAAAATTGAAGACCAAGCTCGGCGTATCGCTGGAAGCCGCGTTTCTGATTCTGGAAGTGGCTGCCAAGT**<br>**TAGGTACTACTGTGGAGGAGGCCGTAAAACGTGCACTGAAGTTGAAGACGAAACTGGGCTAA**<br>MGHHHHHHGSGSGENLYFQGGGSG**VSLEQALQILSVAAQLGTTVEEAVKRALWLKTKLGVSLEQALLILSVAAELGTTVEEAVK**<br>**RALKLKTKLGVSLEQALLILFAAAKLGTTVEEAVKRALKLKTKLGVSLEQALLILYVAAELGTTVEEAVKRALKLKTKLGVSLLQA**<br>**LIILVIAAELGTTVEEAVYRALKLKTKLGVSLEAAFLILEVAAKLGTTVEEAVKRALKLKTKLG**
**dnTRP_18**	ATGGGCCATCATCACCACCATCATGGCAGCGGCAGTGGCGAAAATCTGTATTTTCAAGGTGGCGGGTCTGGC**GTGAGCCTG**<br>**GAACAGGCGCTGCTGATTCTGAGCGTGGCCGCCCTGCTGGGTACCACCGTTGAAGAAGCGGTGAAACGTGCGTTATGGTT**<br>**AAAAACCAAACTGGGCGTCAGCTTGGAACAGGCGGCGTTTATTTTGTCGGTTGCTGCGTATCTGGGCACCACGGTGGAAG**<br>**AGGCTGTAAAGCGCGCGCTGAAACTGAAAACGAAGTTGGGTGTTTCGTTGGAGCAAGCCTTGTTGATCCTGTTTGCGGCGG**<br>**CCGCGTTGGGCACGACCGTCGAGGAGGCCGTTAAGCGTGCCTTGAAGCTCAAGACCAAGCTGGGTGTGAGTTTAGAACAA**<br>**GCACTGTTAATCTTATTTACCGCGGCGGAATTGGGTACTACGGTTGAGGAAGCAGTGAAGCGCGCATTGAAATTGAAGACG**<br>**AAATTAGGCGTTAGTCTGGAGCAGGCGCAGGCGATTCTGGTGGTTGCCGCCGAGTTAGGTACGACGGTAGAGGAGGCGGT**<br>**GTATCGCGCCCTGAAATTAAAGACGAAGCTCGGTGTCTCGTTAGAGCAAGCTTTGCTCATCCTGGAAGTGGCGGCGAAATT**<br>**GGGTACCACTGTGGAGGAGGCTGTCAAACGCGCGTTAAAACTTAAGACTAAATTAGGCTAA**<br>MGHHHHHHGSGSGENLYFQGGGSG**VSLEQALLILSVAALLGTTVEEAVKRALWLKTKLGVSLEQAAFILSVAAYLGTTVEEAVK**<br>**RALKLKTKLGVSLEQALLILFAAAALGTTVEEAVKRALKLKTKLGVSLEQALLILFTAAELGTTVEEAVKRALKLKTKLGVSLEQA**<br>**QAILVVAAELGTTVEEAVYRALKLKTKLGVSLEQALLILEVAAKLGTTVEEAVKRALKLKTKLG**

Supplementary Information

**dnTRP_19**	ATGGGCCATCATCACCACCATCATGGCAGCGGCAGTGGCGAAAATCTGTATTTTCAAGGTGGCGGCTCGGGC**GTGAGCCTG<br>GAACAGGCGCTGCTGATTCTGAGCGTGGCCGCCCTGCTGGGTACCACCGTTGAAGAAGCGGTGAAACGTGCGTTATGGTT<br>AAAAACCAAACTGGGTGTCAGCTTGGAACAGGCGTTGTATATTTTGTCTGTTGCGGCGTATCTGGGCACCACGGTGGAAGA<br>GGCCGTTAAGCGCGCGCTGAAACTGAAAACGAAGTTGGGTGTTAGCCTGCTGCAGGCACTGTGGATTCTGTTTGCCGCGG<br>CGAAATTAGGTACGACGGTAGAGGAGGCAGTCAAACGCGCCTTGAAGCTGAAGACGAAGCTGGGTGTTTCGTTGGATCAG<br>GCCTTGTTGATCCTCTTCGCCGCAGCGGAATTGGGGACTACCGTAGAAGAGGCTGTTAAACGCGCTCTCAAGTTGAAAACT<br>AAATTGGGCGTGTCGTTAGAATTTGCGAATCAGATTCTGGTGGTTGCCGCCGAGTTAGGGACCACTGTCGAGGAAGCCGTT<br>TACCGCGCCCTGAAATTAAAGACTAAGTTAGGCGTCTCGCTGGAGCAGGCGAATCTGATCCTGGAAGTGGCTGCCAAGTT<br>GGGTACCACGGTTGAGGAGGCAGTGAAGCGTGCCCTCAAATTGAAGACCAAGTTAGGCTAA**<br>MGHHHHHHGSGSGENLYFQGGGSG**VSLEQALLILSVAALLGTTVEEAVKRALWLKTKLGVSLEQALYILSVAAYLGTTVEEAVK<br>RALKLKTKLGVSLLQALWILFAAAKLGTTVEEAVKRALKLKTKLGVSLDQALLILFAAAELGTTVEEAVKRALKLKTKLGVSLEFA<br>NQILVVAAELGTTVEEAVYRALKLKTKLGVSLEQANLILEVAAKLGTTVEEAVKRALKLKTKLG**
**dnTRP_20**	ATGGGCCATCATCACCACCATCATGGCAGCGGCAGTGGCGAAAATCTGTATTTTCAAGGTGGCGGCTCGGGC**GTGAGCCTG<br>TATCAGGCGCTGGCGATTCTGTATGTTGCGGCGGCCCTGGGCACCACCGTGGAAGAAGCGGTGAAACGCGCGTTATGGTT<br>AAAAACCAAACTGGGCGTAAGCCTGCAGCAGGCGAGCCAAATTTTAGCGTTAGCGGCGGTTGCGGGTACGACGGTTGAG<br>GAGGCCGTTAAACGCGCGCTGAAACTGAAAACGAAGTTGGGTGTTTCGCTGTGGCAAGCGATTTATATTTGGCTGGTGGCG<br>CTGCTGTTGGGCACGACTGTAGAGGAAGCCGTCAAGCGTGCCTTGAAGTTGAAGACGAAATTGGGCGTTAGTCTGGAACA<br>GGCCCTCAAAATTCTGATTGCCGCGCAGGCCTTGGGTACTACCGTTGAGGAGGCAGTAAAACGTGCTTTGAAACTCAAGAC<br>CAAATTAGGCGTTTCGTTGGAGCAAGCCCTGATTATTCTGTTTGCTGCAGCGGAACTGGGGACGACCGTAGAAGAAGCAGT<br>GTATCGCGCCTTAAAGCTGAAGACTAAGCTCGGTGTATCTCTCTACCAGGCGGCGATTATTAATGCGGTAGCGGCAAAACT<br>CGGGACCACTGTGGAGGAGGCCGTCAAACGCGCCTTGAAATTAAAGACTAAACTTGGCTAA**<br>MGHHHHHHGSGSGENLYFQGGGSG**VSLYQALAILYVAAALGTTVEEAVKRALWLKTKLGVSLQQASQILALAAVAGTTVEEAV<br>KRALKLKTKLGVSLWQAIYIWLVALLLGTTVEEAVKRALKLKTKLGVSLEQALKILIAAQALGTTVEEAVKRALKLKTKLGVSLEQ<br>ALIILFAAAELGTTVEEAVYRALKLKTKLGVSLYQAAIINAVAAKLGTTVEEAVKRALKLKTKLG**
**dnTRP_21**	ATGGGCCATCATCACCACCATCATGGCAGCGGCAGTGGCGAAAATCTGTATTTTCAAGGTGGCGGGTCGGGC**GTGAGCCTG<br>TATCAGGCGCTGGAAATTCTGTTTGTTGCGGCGGCCCTGGGCACCACCGTGGAAGAAGCGGTGAAACGCGCGTTGTGGTT<br>GAAAACCAAACTGGGCGTTAGCCTGCAGCAGGCCGCGCAAATTTTGAATTTTGCAGCGGTTGCGGGTACCACGGTTGAGG<br>AGGCCGTTAAACGCGCGCTGAAACTGAAAACGAAGCTCGGCGTCAGTCTGAGCCAGGCGATTTATATTTGGCTGGTGGCG<br>TTTCTGTTGGGGACGACGGTGGAGGAAGCTGTTAAGCGTGCCTTGAAGTTGAAGACGAAGTTGGGTGTAAGTCTGGAACA<br>GGCATTAAAAATCCTGGTGATTGCGGCTGCCTTGGGTACTACCGTCGAGGAGGCAGTAAAGCGCGCATTGAAGCTCAAGA<br>CCAAGCTGGGGGTTTCGCTTGAGCAAGCACTGCTGATTCTGGCACTGGCGTGGGTTTTAGGCACGACTGTTGAGGAGGCA<br>GTGTATCGCGCCCTGAAGCTGAAGACCAAATTGGGCGTGTCGTTGTACCAGGCTGCCCTGATTAATGCGGTGGCCGCGAA<br>ATTAGGTACGACCGTAGAAGAGGCGGTCAAACGTGCGTTAAAATTGAAGACTAAACTCGGCTAA**<br>MGHHHHHHGSGSGENLYFQGGGSG**VSLYQALEILFVAAALGTTVEEAVKRALWLKTKLGVSLQQAAQILNFAAVAGTTVEEAV<br>KRALKLKTKLGVSLSQAIYIWLVAFLLGTTVEEAVKRALKLKTKLGVSLEQALKILVIAAALGTTVEEAVKRALKLKTKLGVSLEQA<br>LLILALAWVLGTTVEEAVYRALKLKTKLGVSLYQAALINAVAAKLGTTVEEAVKRALKLKTKLG**
**dnTRP_22**	ATGGGCCATCATCACCACCATCATGGCAGCGGCAGTGGCGAAAATCTGTATTTTCAAGGTGGCGGGTCGGGC**GTGAGCCT<br>GCGCCAGGCGGCCAATATTTTAGCGGTTGCGGCGGCGCTGGGTACCACCGTTGAAGAAGCGGTGAAACGTGCGTTATGG<br>TTAAAAACCAAACTGGGCGTTAGTCTGTGGCAGGCGTTTGCGATTTTATTATTAGCGGCCGCCGCGGGCACCACGGTGGA<br>AGAGGCTGTTAAGCGCGCGCTGAAACTGAAAACGAAGCTCGGTGTTAGCCTGGAACAGGCGATTGCCATTCTGTGGGTG<br>GCGCGCATTCTGGGGACGACGGTAGAGGAGGCCGTTAAACGCGCCTTGAAGCTGAAGACGAAGTTGGGTGTTTCGTTGG<br>AGCAGGCGCTGTATATTCTTGCGACCGCGCGCCAGTTGGGCACGACCGTCGAGGAAGCAGTGAAGCGTGCCCTCAAGTT<br>AAAGACCAAATTAGGTGTCTCTTTGGAACAAGCCCTGTTTATTTTGCAAGCGGCGCGTTGGTTGGGTACTACTGTGGAGGA<br>GGCGGTGTATCGCGCTTTGAAATTGAAAACTAAATTGGGCGTATCGCTGCATCAGGCACTGAGCATTCTGGTGGTTGCAG<br>CGAAATTGGGCACTACCGTGGAGGAGGCCGTCAAACGCGCATTAAAACTCAAGACTAAATTAGGCTAA**<br>MGHHHHHHGSGSGENLYFQGGGSG**VSLRQAANILAVAAALGTTVEEAVKRALWLKTKLGVSLWQAFAILLLAAAAGTTVEEA<br>VKRALKLKTKLGVSLEQAIAILWVARILGTTVEEAVKRALKLKTKLGVSLEQALYILATARQLGTTVEEAVKRALKLKTKLGVSLE<br>QALFILQAARWLGTTVEEAVYRALKLKTKLGVSLHQALSILVVAAKLGTTVEEAVKRALKLKTKLG**

Uncropped scans of all blots and gels

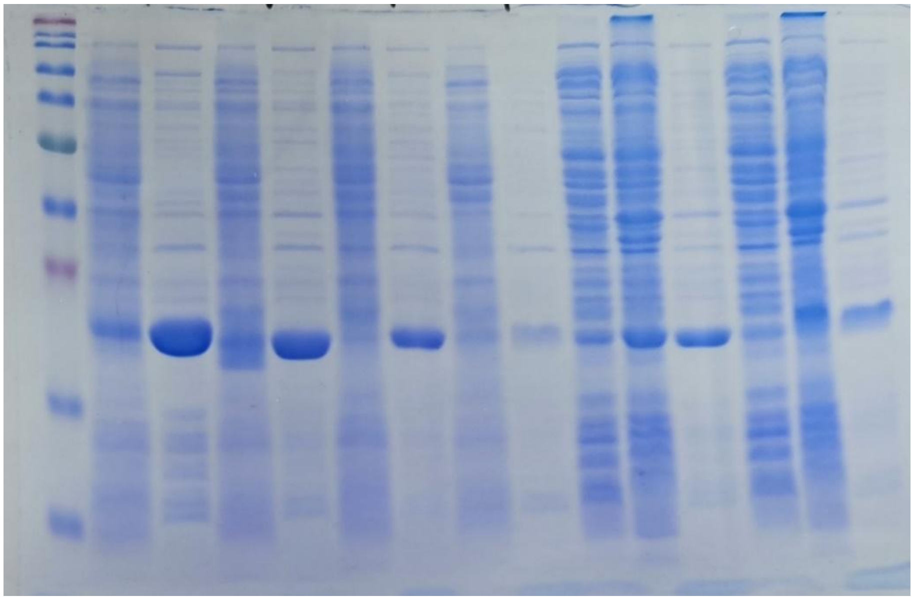

Gel 1: Uncropped sodium dodecyl sulfate–polyacrylamide gel electrophoresis (SDS-PAGE) gel of Supplementary Fig.2 (top, left).

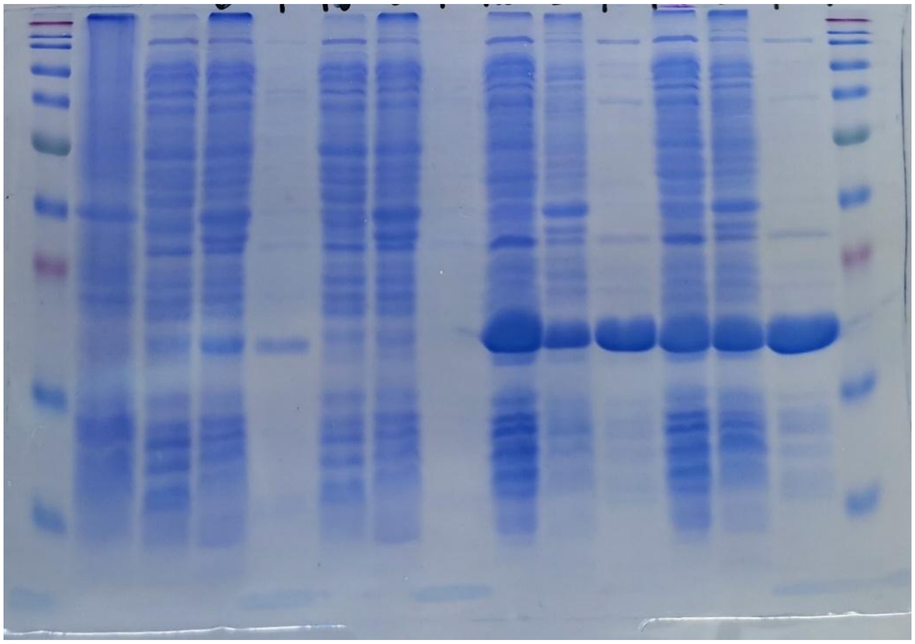

Gel 2: Uncropped SDS-PAGE gel of Supplementary Fig.2 (top, middle).

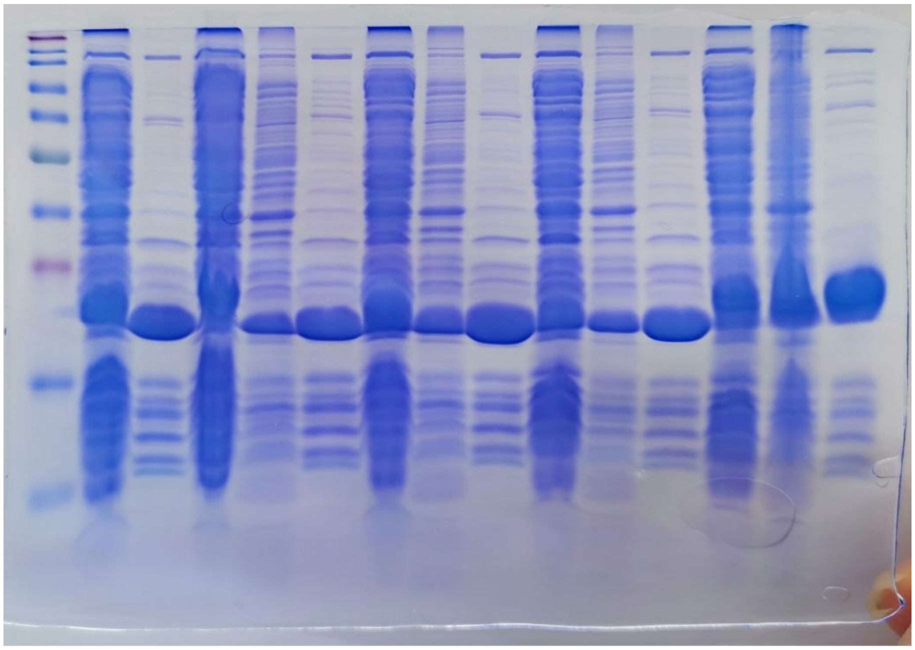

Gel 3: Uncropped SDS-PAGE gel of Supplementary Fig.2 (bottom, left).

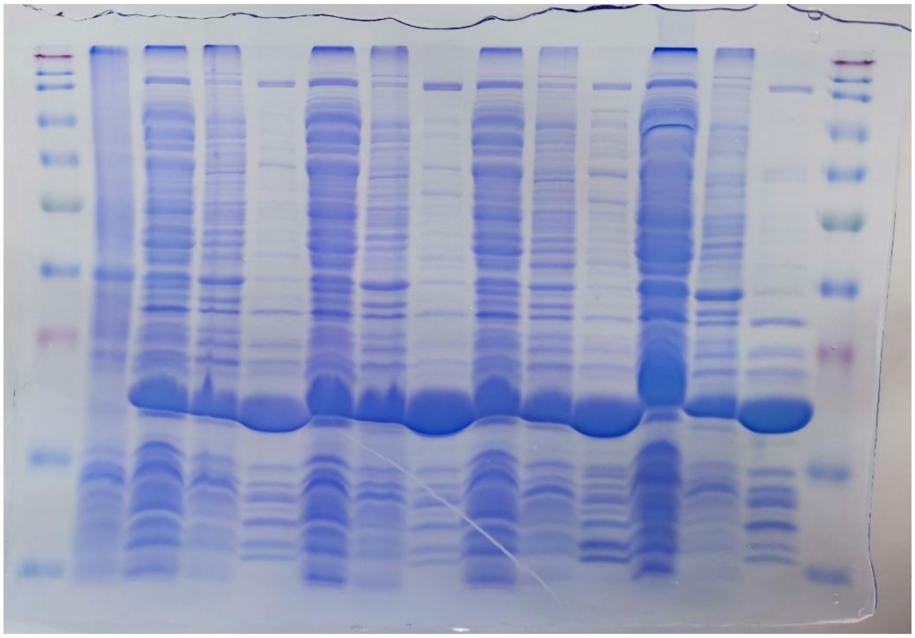

Gel 4: Uncropped SDS-PAGE gel of Supplementary Fig.2 (bottom, middle).

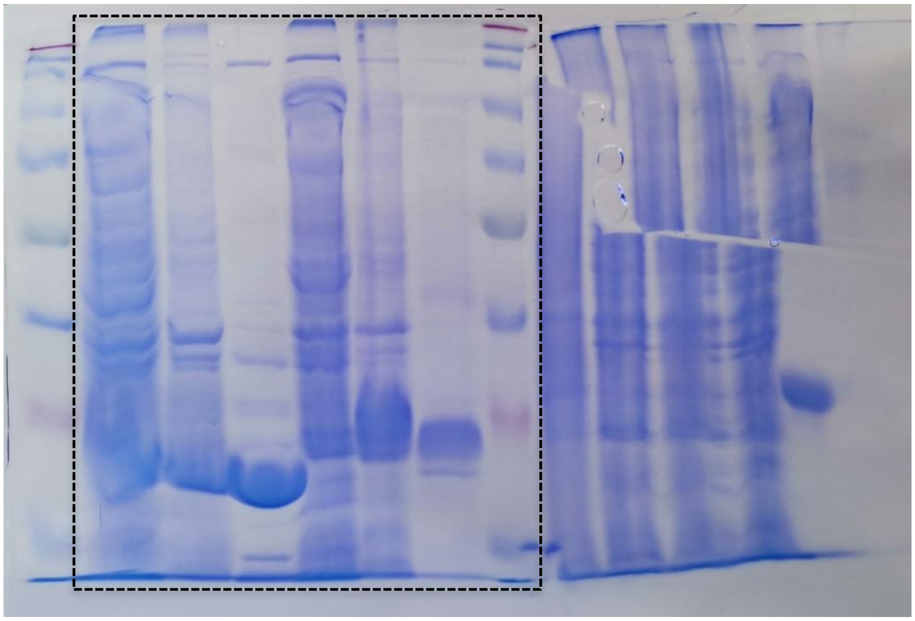

Gel 5: Uncropped SDS-PAGE gel of Supplementary Fig.2 (bottom, right). The parts displayed in Fig. 2 are highlighted in a dashed rectangle.

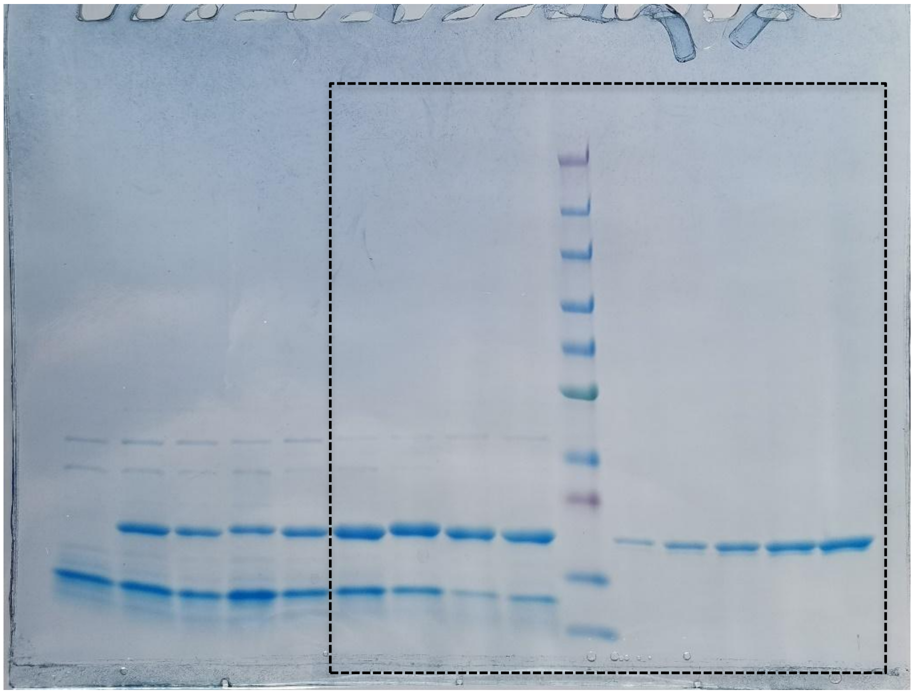

Gel 6: Uncropped SDS-PAGE gel of Supplementary Fig.7a. The parts displayed in Fig. 7a are highlighted in a dashed rectangle.

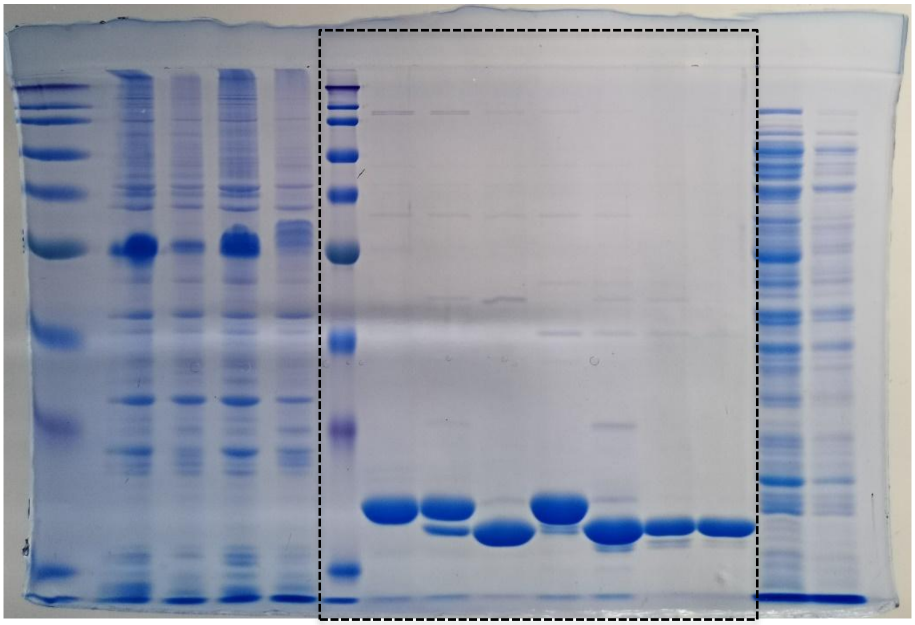

Gel 7: Uncropped SDS-PAGE gel of Supplementary Fig. 9a. The parts displayed in Fig. 9a are highlighted a dashed rectangle.

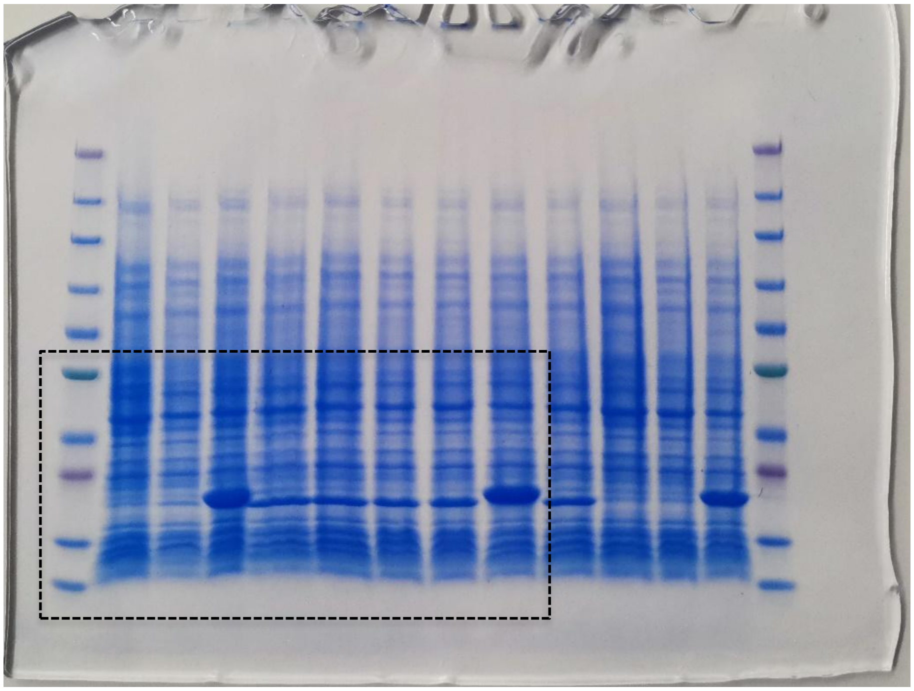

Gel 8: Uncropped SDS-PAGE gel of Supplementary Fig. 9d. The parts displayed in Fig. 9d are highlighted in a dashed rectangle.

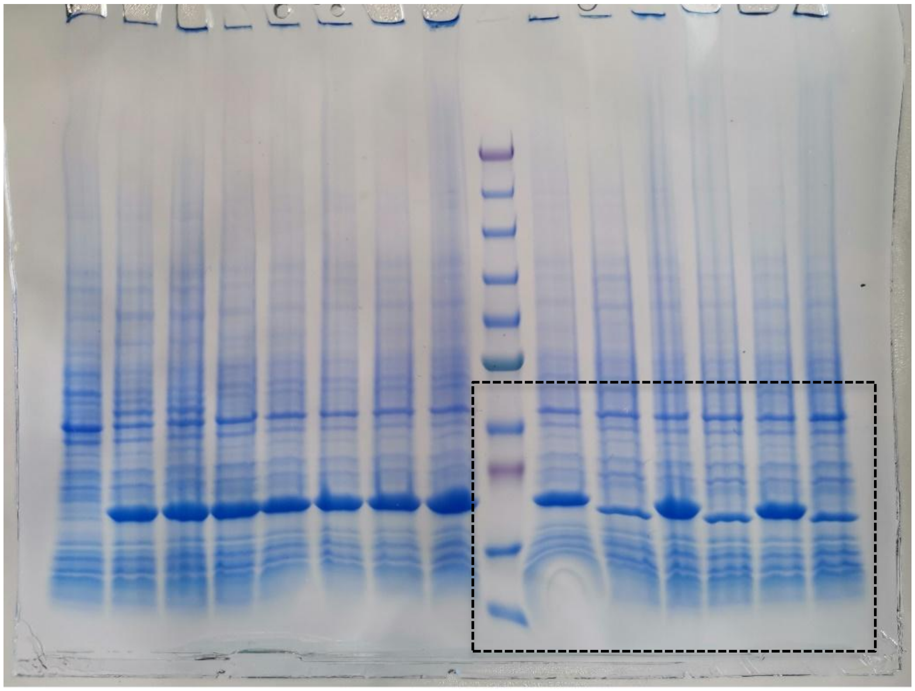

Gel 9: Uncropped SDS-PAGE gel of Supplementary Fig. 15a. The parts displayed in Fig. 15a are highlighted in a dashed rectangle.

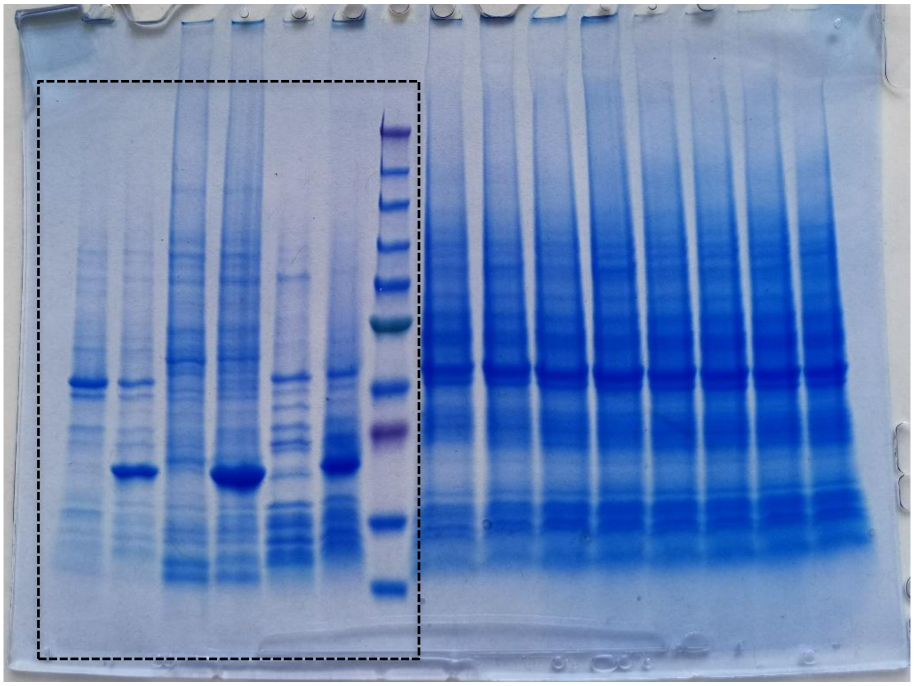

Gel 10: Uncropped SDS-PAGE gel of Supplementary Fig. 16b. The parts displayed in Fig. 16b are highlighted in a dashed rectangle.

Supplementary References

1. Jeschek, M. et al. Directed evolution of artificial metalloenzymes for in vivo metathesis. *Nature* **537**, 661-665 (2016).
2. Fischer, S., Ward, T.R. & Liang, A.D. Engineering a Metathesis-Catalyzing Artificial Metalloenzyme Based on HaloTag. *ACS Catal.* **11**, 6343-6347 (2021).
3. Sabatino, V., Rebelein, J.G. & Ward, T.R. "Close-to-Release": Spontaneous Bioorthogonal Uncaging Resulting from Ring-Closing Metathesis. *J. Am. Chem. Soc.* **141**, 17048-17052 (2019).
4. Ax, A. et al. Cyclic sulfamide HIV-1 protease inhibitors, with sidechains spanning from P2/P2′ to P1/P1′. *Bioorgan. Med. Chem.* **13**, 755-764 (2005).
5. Schmidt, B., Krehl, S. & Jablowski, E. Assisted tandem catalytic RCM-aromatization in the synthesis of pyrroles and furans. *Org. Biomol. Chem.* **10**, 5119-5130 (2012).
6. Anglada, L., Marquez, M., Sacristan, A. & Ortiz, J.A. Inhibitors of gastric acid secretion: N-sulphonyl formamidines in a series of new histamine Hz-receptor antagonists. *Eur. J. Med. Chem.* **23**, 97-100 (1988).
7. Jullien, H. et al. Cyclometalated N-Heterocyclic Carbene-Platinum Catalysts for the Enantioselective Cycloisomerization of Nitrogen-Tethered 1,6-Enynes. *Adv. Synth. Catal.* **353**, 1109-1124 (2011).
8. Lübbe, C., Dumrath, A., Neumann, H., Beller, M. & Kadyrov, R. Lewis Acid Assisted Ruthenium-Catalyzed Metathesis Reactions. *ChemCatChem* **6**, 105-108 (2014).
9. Kawauchi, D. et al. Aerobic Dehydrogenation of N-Heterocycles with Grubbs Catalyst: Its Application to Assisted-Tandem Catalysis to Construct N-Containing Fused Heteroarenes. *Chem. Eur. J.* **26**, 15793-15798 (2020).
10. Frisch, M.J. et al. (Wallingford, CT; 2016).
11. Grimme, S., Ehrlich, S. & Goerigk, L. Effect of the Damping Function in Dispersion Corrected Density Functional Theory. *J. Comput. Chem.* **32**, 1456-1465 (2011).
12. O'Boyle, N.M. et al. Open Babel: An open chemical toolbox. *J. Cheminf.* **3** (2011).
13. Dou, J.Y. et al. De novo design of a fluorescence-activating β-barrel. *Nature* **561**, 485-491 (2018).
14. Jumper, J. et al. Highly accurate protein structure prediction with AlphaFold. *Nature* **596**, 583-589 (2021).
15. Kalvet, I. Sep, 2025 (Github, Github; 2025). https://github.com/ikalvet/denovo_metathase_design.
16. Doyle, L. et al. Rational design of alpha-helical tandem repeat proteins with closed architectures. *Nature* **528**, 585-588 (2015).
17. Maguire, J.B. et al. Perturbing the energy landscape for improved packing during computational protein design. *Proteins: Struct., Funct., Bioinf.* **89**, 436-449 (2021).
18. Kabsch, W. XDS. *Acta Crystallogr. D Biol. Crystallogr.* **66**, 125-132 (2010).
19. Evans, P.R. & Murshudov, G.N. How good are my data and what is the resolution? *Acta Crystallogr. D Biol. Crystallogr.* **69**, 1204-1214 (2013).
20. Potterton, L. et al. CCP4i2: the new graphical user interface to the CCP4 program suite. *Acta Crystallogr. D. Struct. Biol.* **74**, 68-84 (2018).

21. Vagin, A. & Teplyakov, A. Molecular replacement with MOLREP. *Acta Crystallogr. D Biol. Crystallogr.* **66**, 22-25 (2010).
22. Murshudov, G.N. et al. REFMAC5 for the refinement of macromolecular crystal structures *Acta Crystallogr. D Biol. Crystallogr.* **67**, 355-367 (2011).
23. Emsley, P., Lohkamp, B., Scott, W.G. & Cowtan, K. Features and development of Coot. *Acta Crystallogr. D Biol. Crystallogr.* **66**, 486-501 (2010).
24. Moriarty, N.W., Grosse-Kunstleve, R.W. & Adams, P.D. electronic Ligand Builder and Optimization Workbench (eLBOW): a tool for ligand coordinate and restraint generation. *Acta Crystallogr. D Biol. Crystallogr.* **65**, 1074-1080 (2009).
25. Moriarty, N.W., Draizen, E.J. & Adams, P.D. An editor for the generation and customization of geometry restraints. *Acta Crystallogr. D Struct. Biol.* **73**, 123-130 (2017).
26. Discovery, C. et al. Chai-1: Decoding the molecular interactions of life. *bioRxiv*, 2024.2010.2010.615955 (2024).
27. Abramson, J. et al. Accurate structure prediction of biomolecular interactions with AlphaFold 3. *Nature* **630**, 493-500 (2024).
28. Wohlwend, J. et al. Boltz-1 Democratizing Biomolecular Interaction Modeling. *bioRxiv*, 2024.2011.2019.624167 (2024).
29. Anishchenko, I. et al. Modeling protein-small molecule conformational ensembles with ChemNet. *bioRxiv*, 2024.2009.2025.614868 (2024).
30. Park, H., Zhou, G.F., Baek, M., Baker, D. & DiMaio, F. Force Field Optimization Guided by Small Molecule Crystal Lattice Data Enables Consistent Sub-Angstrom Protein-Ligand Docking. *J. Chem. Theory Comput.* **17**, 2000-2010 (2021).
