# Supplementary material for: De novo design and evolution of an artificial metathase for cytoplasmic olefin metathesis
Source: Nat Catal. 2025 Nov 3;8(11):1208–19. doi: 10.1038/s41929-025-01436-0 (PMC12638248; doi:10.1038/s41929-025-01436-0)
Supplement: Supplementary file 2 — Reporting Summary [file 41929_2025_1436_MOESM2_ESM.pdf]

nature portfolio

Corresponding author(s): Thomas R. Ward

Last updated by author(s): Sep 2, 2025

Reporting Summary

Nature Portfolio wishes to improve the reproducibility of the work that we publish. This form provides structure for consistency and transparency in reporting. For further information on Nature Portfolio policies, see our Editorial Policies and the Editorial Policy Checklist.

Statistics

For all statistical analyses, confirm that the following items are present in the figure legend, table legend, main text, or Methods section.

n/a	Confirmed	
☐	☒	The exact sample size (*n*) for each experimental group/condition, given as a discrete number and unit of measurement
☐	☒	A statement on whether measurements were taken from distinct samples or whether the same sample was measured repeatedly
☒	☐	The statistical test(s) used AND whether they are one- or two-sided<br>*Only common tests should be described solely by name; describe more complex techniques in the Methods section.*
☒	☐	A description of all covariates tested
☒	☐	A description of any assumptions or corrections, such as tests of normality and adjustment for multiple comparisons
☐	☒	A full description of the statistical parameters including central tendency (e.g. means) or other basic estimates (e.g. regression coefficient) AND variation (e.g. standard deviation) or associated estimates of uncertainty (e.g. confidence intervals)
☒	☐	For null hypothesis testing, the test statistic (e.g. *F*, *t*, *r*) with confidence intervals, effect sizes, degrees of freedom and *P* value noted<br>*Give P values as exact values whenever suitable.*
☒	☐	For Bayesian analysis, information on the choice of priors and Markov chain Monte Carlo settings
☒	☐	For hierarchical and complex designs, identification of the appropriate level for tests and full reporting of outcomes
☒	☐	Estimates of effect sizes (e.g. Cohen's *d*, Pearson's *r*), indicating how they were calculated

Our web collection on statistics for biologists contains articles on many of the points above.

Software and code

Policy information about availability of computer code

Data collection	Experimental data are collected in various softwares of different instruments, including Waters Acquity UPLC system, SHIMADZU GCMS-QP2020, TECAN i-control 2.0, AlphaFold2&3, Gaussian 16, RifGen, and RifDock softwares. The computed atomic coordinates of the ligand used in this study are available in multiple formats on GitHub using this link: https://github.com/ikalvet/denovo_metathase_design.
Data analysis	Experimental data present in the manuscript and supporting information are processed by different softwares, including Microsoft Excel software (2016), OriginLab Origin2019b, GraphPad Prism, and PyMol 2.3.0.

For manuscripts utilizing custom algorithms or software that are central to the research but not yet described in published literature, software must be made available to editors and reviewers. We strongly encourage code deposition in a community repository (e.g. GitHub). See the Nature Portfolio guidelines for submitting code & software for further information.

Data

Policy information about availability of data

All manuscripts must include a data availability statement. This statement should provide the following information, where applicable:

- Accession codes, unique identifiers, or web links for publicly available datasets
- A description of any restrictions on data availability
- For clinical datasets or third party data, please ensure that the statement adheres to our policy

The data that support the findings in this study are available within this article and the Supplementary Information. The PDB accession codes of apo dnTRP_R0-Δhis, Ru1·R0-Δhis, and Ru1·R5-Δhis are 9GVF, 8S6P, and 9H3C, respectively. All other data is available from the authors upon request.

Human research participants

Policy information about studies involving human research participants and Sex and Gender in Research.

Reporting on sex and gender	n/a
Population characteristics	n/a
Recruitment	n/a
Ethics oversight	n/a

Note that full information on the approval of the study protocol must also be provided in the manuscript.

Field-specific reporting

Please select the one below that is the best fit for your research. If you are not sure, read the appropriate sections before making your selection.

☒ Life sciences ☐ Behavioural & social sciences ☐ Ecological, evolutionary & environmental sciences

For a reference copy of the document with all sections, see nature.com/documents/nr-reporting-summary-flat.pdf

Life sciences study design

All studies must disclose on these points even when the disclosure is negative.

Sample size	In this study, no specific sample size was determined for performing the ring-closing metathesis, either for in vitro or in vivo experiments. Screening of the artificial metathase at influential amino acid residues, as well as random residues (generated by error-prone PCR) was conducted. This process identified the artificial metathase variants that exhibited enhanced binding affinity to the designed cofactor and improved catalytic turnover numbers in the ring-closing metathesis.
Data exclusions	No data were excluded.
Replication	Data presented in the manuscript were performed in triplicates or more. Data collected in the Supplementary Information were performed in one or more replicates. All replication experiments were successful.
Randomization	Site-directed and site-saturation mutagenesis libraries of artificial metathase were generated by targeting the amino acid residues that are in proximity to the metal cofactor. Random mutagenesis libraries were generated by error-prone PCR and DNA fragment shuffling. For the site-directed mutagenesis clones, colonies with the correct mutational sequences were processed for protein expression and subsequent catalytic experiments. For the site-saturation and random mutagenesis libraries, mixed plasmids were used. During the screening process, colonies were randomly selected for enzyme expression. Only those variants with improved catalytic turnover numbers were sequenced, and the corresponding variants were selected for further characterization.
Blinding	No blind experiments were performed in this work as this is not a general procedure in the field.

Reporting for specific materials, systems and methods

We require information from authors about some types of materials, experimental systems and methods used in many studies. Here, indicate whether each material, system or method listed is relevant to your study. If you are not sure if a list item applies to your research, read the appropriate section before selecting a response.

Materials & experimental systems

n/a	Involved in the study
☒	☐ Antibodies
☒	☐ Eukaryotic cell lines
☒	☐ Palaeontology and archaeology
☒	☐ Animals and other organisms
☒	☐ Clinical data
☒	☐ Dual use research of concern

Methods

n/a	Involved in the study
☒	☐ ChIP-seq
☒	☐ Flow cytometry
☒	☐ MRI-based neuroimaging
